# Design and application of a fluorescent probe for imaging of endogenous Bruton's tyrosine kinase with preserved enzymatic activity†

**DOI:** 10.1039/d4cb00313f

**Published:** 2025-02-20

**Authors:** Anna P. Valaka, Hampus Nyström, Liliana Håversen, Carlos Benitez-Martin, Clara Schäfer, Woo Suk Jang, Alessandro Camponeschi, Joakim Andréasson, Jan Borén, Morten Grøtli

**Affiliations:** a Department of Chemistry and Molecular Biology, University of Gothenburg 405 30 Gothenburg Sweden grotli@chem.gu.se; b Department of Molecular and Clinical Medicine, University of Gothenburg and Sahlgrenska University Hospital 413 45 Gothenburg Sweden; c Department of Rheumatology and Inflammation Research, Institute of Medicine, Sahlgrenska Academy, University of Gothenburg Gothenburg 413 46 Sweden; d Department of Chemistry and Chemical Engineering, Chalmers University of Technology 412 96 Gothenburg Sweden

## Abstract

Fluorophore integration into proteins within living cells is essential for exploring proteins in their natural environment. Bruton's tyrosine kinase (BTK), is a validated oncology target and is crucial for B cell proliferation and activation. Developing BTK-labelling probes is key to understand BTK's dynamic signalling pathway. In this work, we aimed to develop a novel fluorescent labelling probe for endogenous BTK imaging while preserving its enzymatic activity. Evobrutinib, a second-generation BTK inhibitor with high selectivity, was chosen as the scaffold. We designed two probes, Evo-1 and Evo-2, with a BODIPY fluorescent group, guided by molecular modelling. The synthesis was achieved using optimised Suzuki–Miyaura cross-coupling and amide coupling reactions. Biochemical assays confirmed covalent binding to Cys481 of BTK while preserving its enzymatic activity. Labelling of endogenous BTK with Evo-2 with reduced off-target effects in Ramos cells was validated in cellular assays. The dynamic signalling pathway of BTK in its native environment was investigated by confocal microscopy with Evo-2. This methodology is a valuable asset in the chemical biology toolbox for studying protein dynamics and interactions in real time without interfering with the protein activity.

## Abstract 2

Development of a novel fluorescent probe for endogenous BTK imaging using evobrutinib as a scaffold. Evo-2 enabled real-time visualisation of BTK dynamics in living cells while preserving its enzymatic activity.

## Graphical abstract

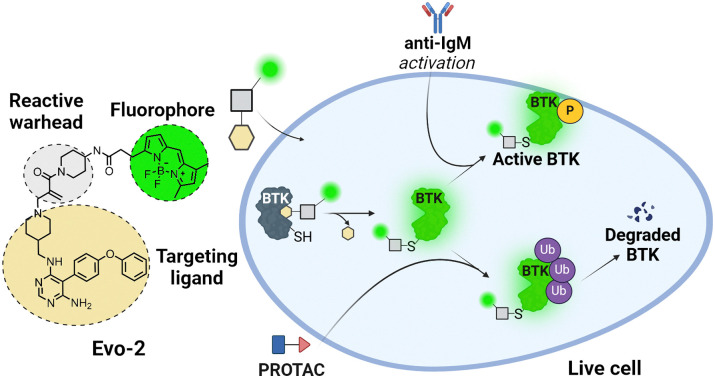

## Introduction

Chemical modification of proteins in their natural environment, (*i.e.* living cells), has emerged as a powerful tool for elucidating the structure, function, localisation, and dynamics of a specific protein of interest (POI). Genetic engineering has been extensively employed to modify and label proteins.^1^ Fusion proteins can be created by joining the POI with a fluorescent domain (*e.g.* the green fluorescent protein, GFP), or a domain with a chemically reactive handle such as SNAP-tag^2^ or HaloTag.^3^ However, the relatively big size of these reporter tags often perturbs the native structure and function of the studied protein. These issues can be overcome by incorporating unnatural amino acids into the POI followed by a bio-orthogonal reaction with a reactive handle.^4,5^ Bio-orthogonal chemistry has expanded the number of proteins that can be modified in cellular environments. Nonetheless, these methodologies require engineered cells, hence limiting their scope. Non-invasive protein labelling enables the study of function, conformation, and cellular signalling pathways, avoiding the need for genetic engineering methods. In this approach, small-molecule probes are used to selectively modify naturally occurring amino acids in the POI. Affinity-labelling probes are comprised of a target recognition group, a reactive functionality, and a tag.^6^ Although non-invasive, these probes often suppress the protein's native activity, impeding the study of the POI's involvement in relevant cellular processes.

**Fig. 1:**
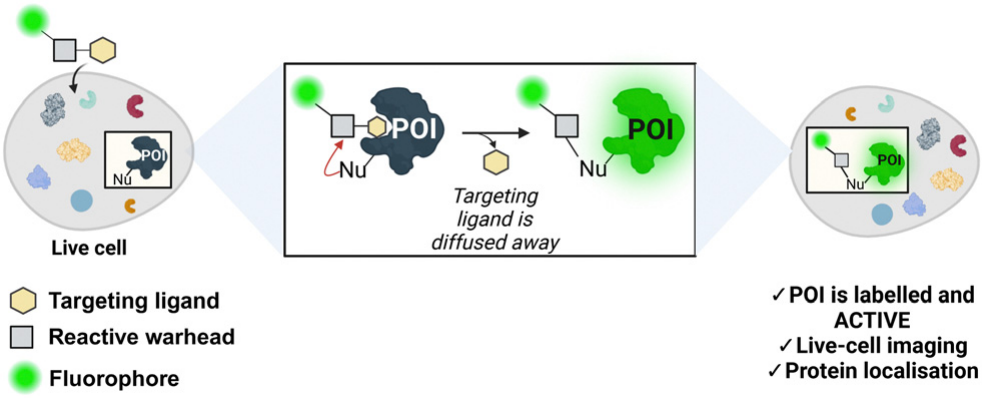
Traceless affinity labelling in live cells. Ligand-directed chemistry probes allow for the selective modification of a protein of interest (POI) in its native cellular environment. The targeting ligand diffuses away, allowing the protein to remain active post-modification. Nu = nucleophile. The figure was created with https://Biorender.com.

Ligand-directed chemistry techniques can solve this problem by incorporating a cleavable electrophile into the labelling probe. This method involves coupling a targeting ligand that recognises the protein's active site with a tag (*e.g.*, a fluorophore), using a reactive electrophilic group known as a warhead (Fig. 1). The warhead enables the covalent attachment of the tag to the protein by reacting with a nucleophilic non-catalytic residue outside of the binding pocket. As a result, the targeting ligand is released and diffuses away from the enzyme, maintaining enzymatic activity post-labelling. Incorporating a fluorescent probe enables the imaging and localisation of the target protein.^7,8^ Hamachi and co-workers first described this principle in the endogenous labelling of FKBP12,^9,10^ and since then, the field has grown immensely with the development and incorporation of novel warheads for protein labelling.^11^ Bruton's tyrosine kinase (BTK) is a non-receptor tyrosine kinase and a member of the Tec family. BTK is mainly expressed in hematopoietic cells including myeloid and lymphoid cells, such as macrophages, monocytes and B-cells, but not in T-cells.^12^

BTK is a key factor in B-cell development, differentiation, survival, and signal transduction, and has been used as a target for treating B-cell malignancies.^13^ Furthermore, BTK has also been validated as a therapeutic target for potential treatment of multiple sclerosis^14^ and several other autoimmune diseases.^15^ To date, six small-molecule BTK inhibitors have been approved for treating various haematological cancers; where five of them are covalent inhibitors targeting the non-catalytic Cys481 residue at the vicinity of its ATP-binding pocket.^16^

BTK is mainly cytosolic, but upon B-cell receptor (BCR) stimulation, it translocates to the plasma membrane to be activated.^17^ BTK exhibits diverse functions contingent upon its cellular location. It plays a pivotal role in cytoplasmic signal transduction, and membrane-associated receptor signalling, and potentially nuclear gene regulation.^18^ Understanding BTK's activity and localisation in live cells is crucial for unravelling its complex signalling pathways. Recent advances in BTK-labelling probes have aimed to enable real-time imaging of endogenous BTK.^19–22^ These probes bind irreversibly to Cys481, inhibiting the activity of endogenous BTK. This limitation has hindered their use in studying BTK's functional role in live-cell environments. Moreover, all reported studies on the enzyme's signalling pathway have involved molecular genetics.^23^ Recently, a BTK-labelling probe was developed that preserved enzymatic activity, highlighting cellular degradation studies ^8^.

In this study, we focused on the development of a probe designed for co-localisation and live-cell fluorescence imaging, showcasing its potential for visualising BTK in its native environment with intact enzymatic activity. Our probe's efficacy in labelling BTK was validated through biochemical and cellular assays, including immunoblotting and immunoprecipitation. Furthermore, co-localisation and live-cell imaging experiments demonstrated the probe's potential as a tool for studying BTK's localisation, expression, degradation, and signalling pathways in live cells. This approach offers a valuable tool to gain insights into BTK's role in cellular processes, providing a foundation for future studies on its therapeutic and diagnostic applications.

## Results and discussion

### Design, synthesis, and characterisation of probes

**Fig. 2:**
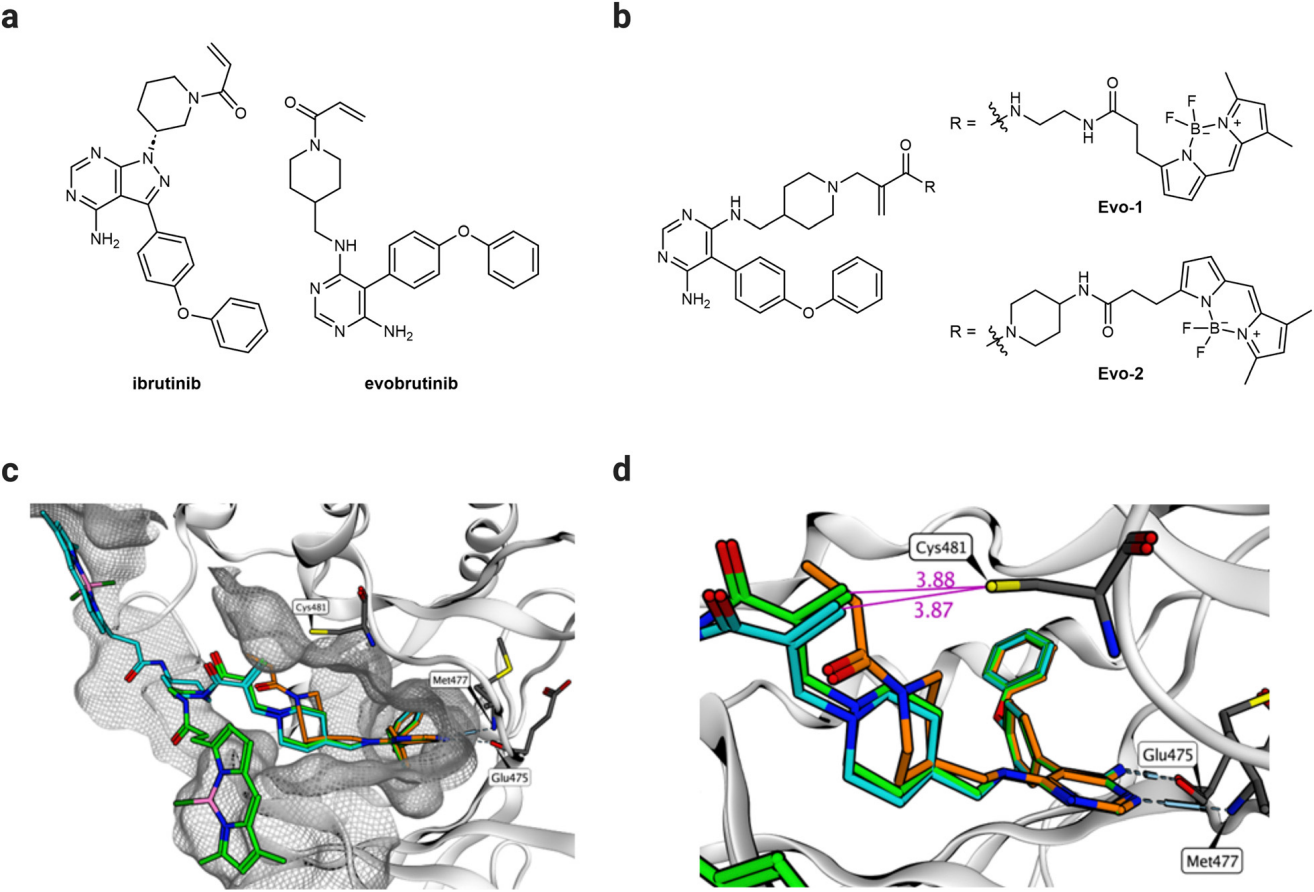
Design of probes. (a) Chemical structures of ibrutinib and evobrutinib. (b) Chemical structures of the probes designed, synthesised, and evaluated in the study (Evo-1, Evo-2). (c) Molecular docking structures of Evo-1 (light green carbons) and Evo-2 (cyan carbons) bound to the kinase domain of BTK superimposed on the crystal structure of evobrutinib (orange carbons, PBD ID: 6OMU). The image shows a 4.5 Å molecular surface of all BTK residues near the ligands. (d) A close-up of (c) but with highlighted distances (Å, purple) between the warhead and Cys481. Hydrogen bonds to the hinge region are illustrated.

We aimed to develop a novel fluorescent labelling probe that enables imaging of endogenous BTK without inhibiting the enzymatic activity. The reported probes employ the FDA-approved BTK inhibitor ibrutinib^24^ as a targeting ligand equipped with a Michael acceptor as a warhead.^8,19–22,25,26^ Despite its potency, ibrutinib exhibits numerous side effects resulting from its off-target activity against the epidermal growth factor receptor (EGFR) and other Tec family kinases.^27^ Interest in developing BTK inhibitors for treating autoimmune diseases has grown in recent years.^16^ Evobrutinib is a second-generation BTK inhibitor, currently being investigated in a Phase 3 clinical trial for treating relapsing multiple sclerosis.^16^ Although it incorporates the same acrylamide warhead, evobrutinib differs from ibrutinib in the hinge-binding aromatic heterocycle and the substitution pattern of the piperidine ring (Fig. 2(a)). Despite their structural similarities, evobrutinib has higher kinome selectivity than ibrutinib. Out of 236 kinases tested, ibrutinib targeted seven Cys481-like kinases, including EGFR, whereas evobrutinib inhibited only BMX and TEC.^28^ BTK, BMX, and TEC are all members of the same family, and their high degree of homology poses a challenge in achieving selectivity.^29^ The 1,4-substitution pattern of the piperidine ring on evobrutinib leads to a more rigid warhead compared to that of the 1,3-substitution in ibrutinib. This constraint is thought to increase the selectivity for BTK over EGFR. Moreover, covalent binding is required for potency. Removing the acrylamide warhead from the evobrutinib scaffold suppresses its inhibitory effect. By contrast, in the case of ibrutinib, potency is still achieved regardless of the warhead.^28^ Therefore, we envisaged that the evobrutinib scaffold would be suitable for developing BTK-imaging probes. We incorporated a BODIPY-fluorescent group as the imaging fluorophore because of its high fluorescent quantum yield, stability, and synthetic feasibility.^30^ We then used molecular modelling of the published crystal structure of evobrutinib bound to the kinase domain of BTK (PDB ID: 6OMU) to design the probes. The structure of the hinge-binding motif was used as a template for designing two BTK-labelling probes (Evo-1, Evo-2), differing in the acrylamide–amine linker (Fig. 2(b)). Evo-2 has a more sterically hindered tertiary acrylamide warhead, which was hypothesised to decrease the reactivity and increase the selectivity compared to Evo-1. The molecular docking results suggest that the binding mode overlapped with that of the parent ligand. Both probes’ phenoxyphenyl and aminopyrimidine moieties superimposed well on evobrutinib (Fig. 2(c) and Fig. S1, ESI†), while the piperidine ring and the warhead had different orientation. The acrylamide warhead was pointed directly toward Cys481, with its reactive warhead positioned within 4 Å from the cysteine residue (Fig. 2(d)), suggesting optimal proximity for covalent bond formation.^31,32^ Finally, the BODIPY group was positioned on the surface of the protein exposed to the solvent.

**Scheme 1:**
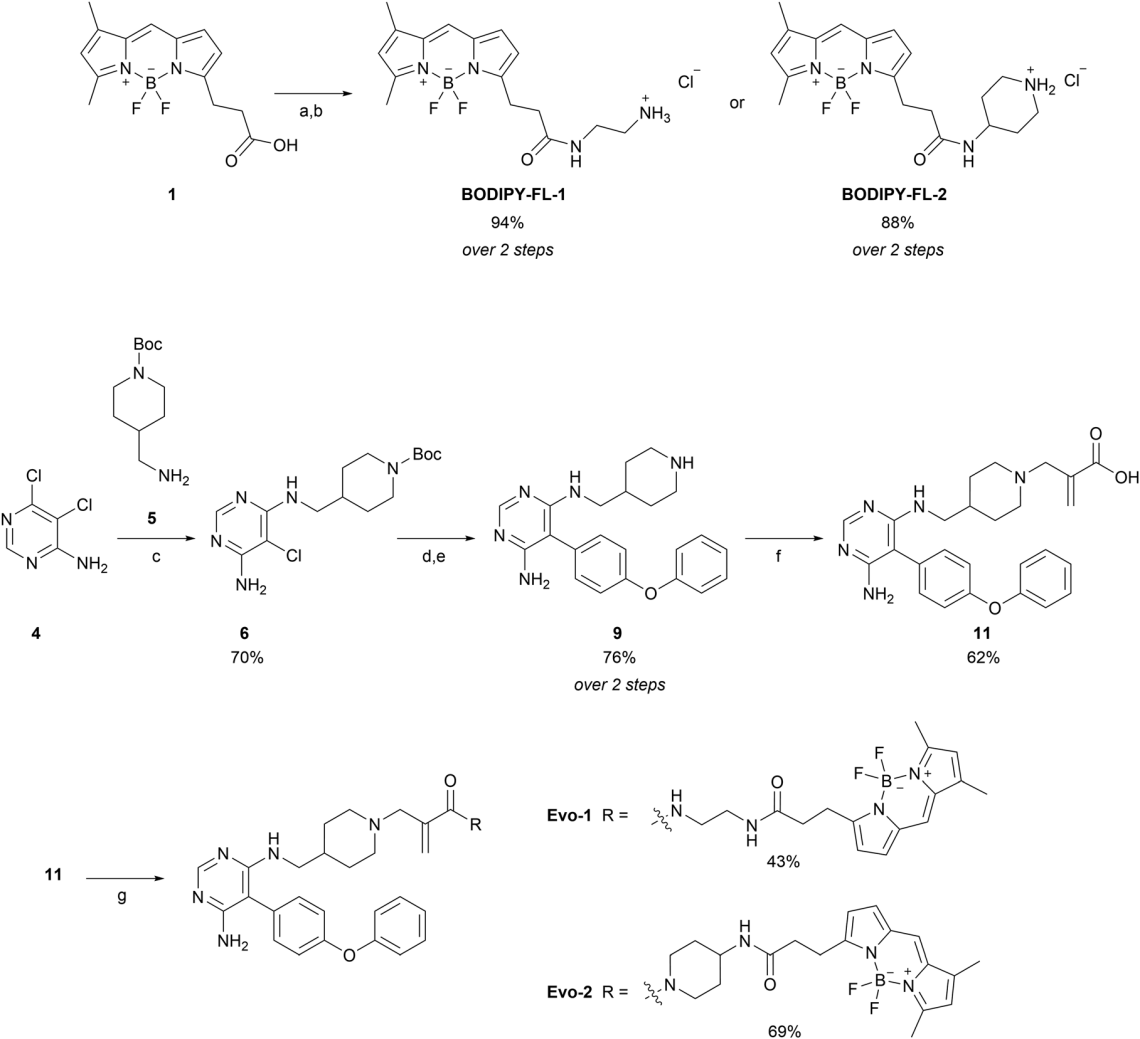
Synthesis of BODIPY-FL linkers and BTK probes. Reagents and conditions: (a) *N*-Boc-ethylenediamine or 4-amino-1-Boc-piperidine (1.2 eq.), HATU (1.2 eq.), DIPEA (1.2 eq.), DCM, room temperature (rt), 30–40 min; (b) HCl in 1,4-dioxane (10.0 eq.), MeOH, 0 °C to rt, 24 h; (c) 5 (1.1 eq.), DBU (2.0 eq.), THF, microwave, 140 °C, 4 h; (d) 4-phenoxyphenylboronic acid (1.5 eq.), SPhos Pd G4 (2.5 mol%), K_2_CO_3_ (2.0 eq.) 1,4-dioxane : H_2_O (4 : 1), 90 °C, 20 h; (e) HCl 4.0 M in 1,4-dioxane (10.0 eq.), MeOH, 0 °C to rt, 18 h; (f) 2-(bromomethyl) acrylic acid (0.9 eq.), DIPEA (1.0 eq.), THF, microwave, 40 °C, 15 min; (g) BODIPY-FL-1 or BODIPY-FL-2 (1.2 eq.), HATU (1.2 eq.), DIPEA (2.4 eq.), DCM, rt, 45–60 min.

The synthetic route of the two probes is depicted in Scheme 1 and all synthetic protocols and characterisation data can be found in the ESI.† We began by synthesising the BODIPY–amine linkers (BODIPY-FL-1 and BODIPY-FL-2). First, BODIPY-acid 1 was prepared following a procedure outlined in the literature.^33^ Subsequently, HATU-mediated coupling reactions were performed, followed by Boc deprotections, yielding the amino linkers as their HCl salts. Synthesis of the evobrutinib precursor 9 was based on a reported procedure,^28^ and started with a nucleophilic aromatic substitution between dichloropyrimidine analogue 4 and amine 5. Next, we focused on the optimisation of the Suzuki–Miyaura cross-coupling reaction between the chloroaminopyrimidine 6 and 4-phenoxyphenylboronic acid. The reported conditions described the formation of the desired product with a yield of 48%. However, we could not reproduce these results (Table S1, ESI,† entry 1). Although XPhos has been reported to be an optimal ligand for the Suzuki coupling reaction of structurally challenging heterocyclic aryl halides,^34^ we observed no improvement in our substrate (entry 2). Thus, we screened different pre-catalyst systems (entries 3–7). The third-generation Buchwald pre-catalyst SPhos Pd G3 demonstrated a substantial increase in the reaction yield, generating the product more efficiently than under the original conditions (2 h and 67% yield *versus* 16 h and 28% yield). Nonetheless, optimal conditions were achieved using SPhos Pd G4 (2.5 mol%) as a catalyst, K_2_CO_3_ (2.0 equivalents) as a base, and 1,4-dioxane : H_2_O in a 4 : 1 ratio as a solvent, at 90 °C for 20 h (entry 7). These conditions furnished the desired product with 76% yield and without the need for column purification. Subsequently, Boc deprotection with HCl in dioxane and basification with NaHCO_3_, afforded the evobrutinib precursor 9 with a yield of 76% over two steps. *N*-Alkylation of 9 with 2-(bromomethyl)acrylic acid yielded compound 11, which was then used in HATU-mediated amide-coupling reactions with the two BODIPY-FL amine linkers to obtain the corresponding probes Evo-1 and Evo-2 with yields of 43% and 69%, respectively.

### Evaluation of the probes in biochemical assays

**Fig. 3:**
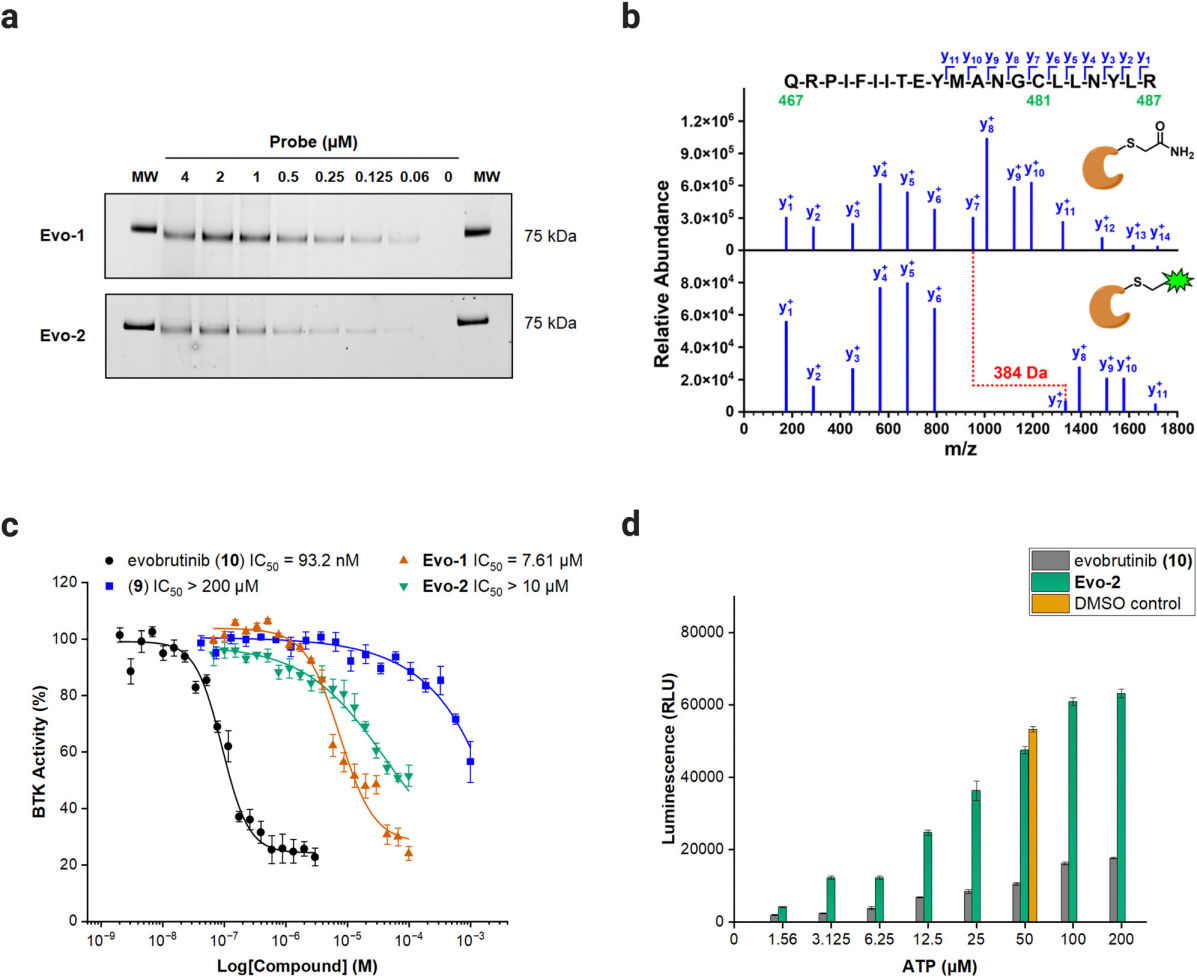
Biochemical evaluation of probes against full-length BTK. (a) Dose-dependent labelling of BTK by Evo-1 and Evo-2. In-gel fluorescence scanning. Recombinant BTK (0.1 μg) was incubated with decreasing concentrations of Evo-1 or Evo-2 for 1 h and then imaged with ChemiDoc apparatus (Alexa488 filter). The molecular weight (*M*_W_) of the fluorescent marker 75 kDa are shown on the far left and right of the gel. (b) MS/MS spectra of the BTK (467–487) tryptic peptide, showing the-single charged y-ion series (the complete raw spectra are available in the ESI†). A 5 μM solution of recombinant full-length BTK was incubated with either DMSO or 50 μM Evo-2. Subsequently, BTK was reduced with DTT, alkylated with iodoacetamide, digested with trypsin, and analysed by LC–MS/MS. (Top) MS/MS spectrum of the +3 charged precursor ion (*m*/*z* = 862.450) of the carbamidomethyl-modified peptide, resulting from BTK incubation with DMSO. Alkylation of Cys481 by iodoacetamide increased its mass by 57 Da. (Bottom) MS/MS spectrum of the +4 charged precursor ion (*m*/*z* = 743.139) of the Evo-2-modified peptide, resulting from BTK incubation with Evo-2. Labelling of Cys481 by Evo-2 increased its mass by 441 Da, or 384 Da compared to the alkylated peptide, as indicated in red. (c) IC_50_ values of evobrutinib (black), compound 9 (blue), Evo-1 (orange) and Evo-2 (green) probes against BTK. An ADP-Glo Assay was performed, as described in the ESI.† Data are presented as the mean ± SD (*n* = 4). (d) ATP-competition assay results with Evo-2. An ADP-Glo Assay was performed, where BTK was treated with 500 nM Evo-2 (green) or evobrutinib (grey) and increasing concentrations of ATP, as described in the ESI.† Control experiments with DMSO under normal assay conditions (50 μM ATP, orange) were also carried out. Luminescence is presented as relative luminescence units (RLU). Data are presented as the mean ± SD (*n* = 3).

With the probes ready, we examined the labelling capability against full-length recombinant BTK. BTK was incubated with increasing concentrations of Evo-1 and Evo-2 for 1 h at room temperature. The samples were then denatured under reducing conditions at 70 °C for 10 min and ran on SDS-PAGE gel. The fluorescence intensity of the anticipated 75 kDa band increased in a dose-dependent manner (Fig. 3(a) and Fig. S2, ESI†), confirming that the probes bind covalently to BTK. Furthermore, proteomics analysis was performed to identify the biding site and validate our anticipated mechanism of action. Recombinant BTK was treated with either Evo-2 or DMSO, followed by first reduction with dithiothreitol (DTT), then alkylation with iodoacetamide, and finally digestion with trypsin. The resulting peptides were analysed by LC–MS/MS, resulting in the identification of the tryptic peptide (467–487) containing Cys481. Manual inspection of the fragment spectra confirmed that the probe bound to Cys481 and no other cysteines. The raw MS/MS spectra (Fig. S3–S7, ESI†) with lists of fragment ions that matched in the database search (Tables S2–S11, ESI†) for multiple precursor ions, as well as the list of all identified peptides are available in the ESI.† The single-charged y-ion series for labelled and unlabelled peptide are illustrated in Fig. 3(b), where the gap between y_6_ and y_7_ corresponds to Cys481. The BTK treated with DMSO has undergone a carbamidomethylation at Cys481, increasing its mass by 57 Da. Meanwhile, Cys481 in BTK treated with Evo-2 exhibited a relative mass shift of 384 Da compared to the DMSO sample, confirming site-specific labelling by the probe.

To verify that the enzymatic activity was retained post-modification, the probes were evaluated for their effect on BTK activity, using the ADP-Glo Kinase Assay.^35^ The half-maximal inhibitory concentrations (IC_50_) of the probes and the parent inhibitor evobrutinib were determined against full-length BTK. Evobrutinib had an IC_50_ of 93.2 nM, whereas high concentration levels of the probes (7.61 μM for Evo-1 and IC_50_ > 10 μM for Evo-2) were required to inhibit the enzyme. The substantial difference between the IC_50_ obtained here and the reported IC_50_ of evobrutinib (8.9 nM)^28^ can be attributed to the different assay conditions used in this study. Removal of the acrylamide warhead from evobrutinib (compound 9), eliminated its potency (IC_50_ > 100 μM), demonstrating that the covalent acrylamide warhead in the evobrutinib scaffold is required for its activity (Fig. 3(c) and Fig. S8, ESI†). Moreover, the modest inhibitory effect observed when using the probes is likely due to the steric hindrance caused by the covalently bound probe in the substrate-binding pocket, hampering the enzymatic activity. To assess whether the probes interfered with ATP-binding, BTK was incubated with 500 nM of either Evo-1, Evo-2, or evobrutinib as a control, followed by a treatment with increasing concentrations of ATP. While evobrutinib suppressed the activity, the luminescence signal readout increased exponentially in the case of probes (Fig. 3(d) and Fig. S9, ESI†).

### Labelling of endogenous BTK in Ramos cells

**Fig. 4:**
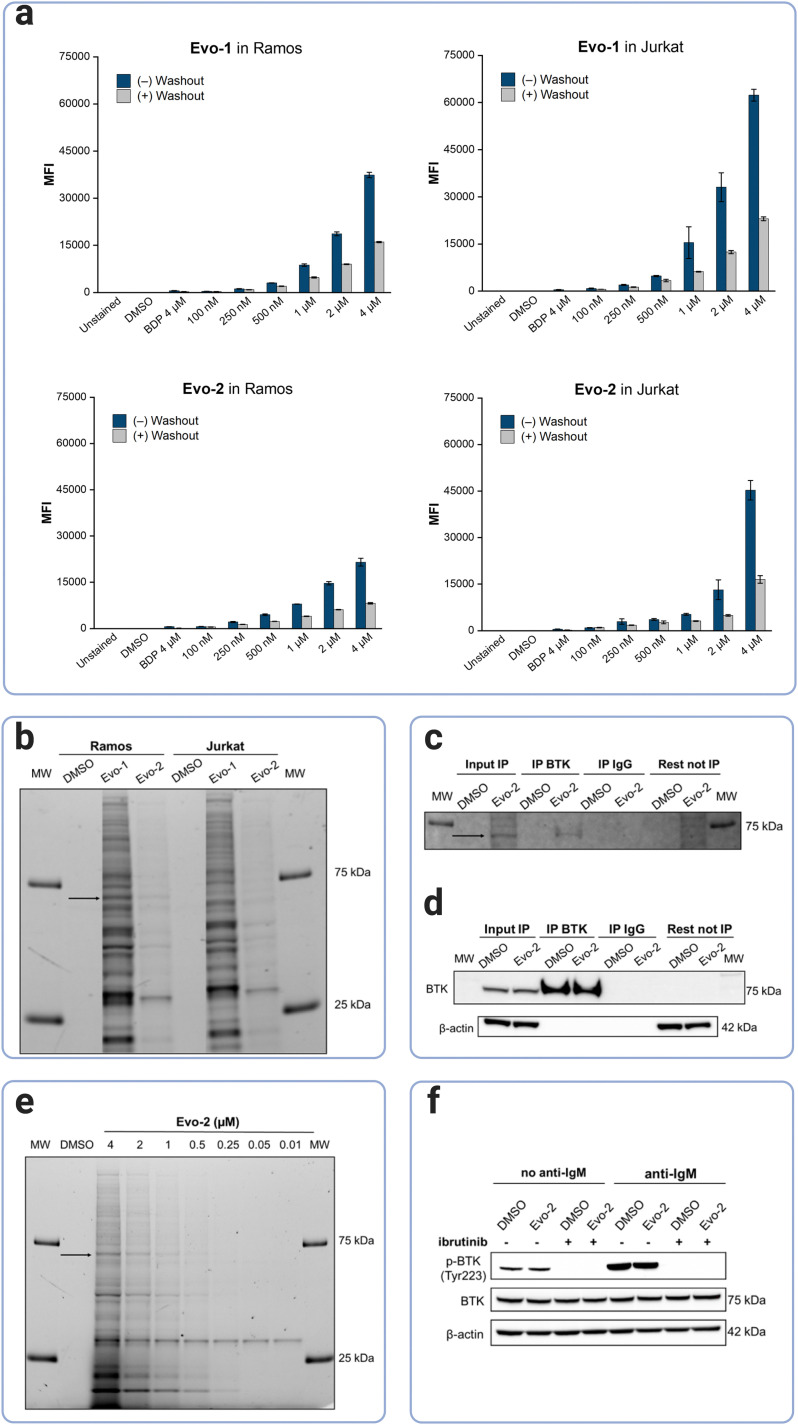
Labelling of endogenous BTK in live cells. (a) Cellular uptake and labelling of living cells by the probes in Ramos and Jurkat cells as analysed by flow cytometry. Cells were incubated with either 0.1% DMSO, 4 μM compound 2 as a BODIPY-control (BDP), or different concentrations of Evo-1 or Evo-2 for 2 h. Fluorescence intensity was measured either directly after the incubation time (blue bars) or after 16 h of washout in probe free media (grey bars). Mean fluorescence intensities (MFIs) were measured on the FITC channel (*λ*_exc_ = 488 nm *λ*_em_ = 527 nm). Data are presented as the mean ± SD (*n* = 3). (b) Cellular protein labelling profile with Evo-1 and Evo-2 in Ramos and Jurkat cells. Cells were incubated with 500 nM of the compound or 0.1% DMSO for 2 h. The cells were washed, lysed, and cellular proteins were separated on SDS-PAGE gel under denatured and reduced conditions. The fluorescence signals of the cellular proteins labelled by the probes were detected using a ChemiDoc apparatus (Alexa488 filter). The black arrow indicates the labelled BTK. MW fluorescent markers of 75 and 25 kDa are shown on the far left and right of the gel. (c) Immunoprecipitation of Evo-2-bound BTK in Ramos cells after treatment with 4 μM Evo-2 for 4 h. Fluorescence detection of the immunoprecipitated BTK bound to Evo-2 (ChemiDoc apparatus, Cy2 filter). The black arrow indicates the labelled BTK. MW fluorescent markers of 75 and 25 kDa are shown on the far left and right of the gel. (d) Chemiluminescence detection of immunoprecipitated BTK after immunoblotting using anti-BTK antibodies. (e) In-gel detection of fluorescent labelled cellular proteins after incubation of Ramos cells with different concentrations of Evo-2 for 2 h. The black arrow indicates the labelled BTK. MW fluorescent markers of 75 and 25 kDa are shown on the far left and right of the gel. (f) Effect of Evo-2 on BTK activity in Ramos cells, assessed by immunoblotting using antibodies against phospho-BTK (Tyr 233) and total BTK. The cells were pre-incubated with either 0.1% DMSO or 5 μM ibrutinib for 30 min, and without washing, the cells were incubated with 500 nM Evo-2 for 2 h. Thereafter, the cells were washed before BCR-stimulation with anti-human IgM (10 μg mL^−1^) for 10 min.

Next, BTK's labelling efficacy was examined at the cellular level. The cell permeability of the probes was investigated by flow cytometry. BTK is highly expressed in B-cells but not in T-cells,^12^ thus, Ramos, a B-lymphocyte cell line (BTK+) and Jurkat, a T-lymphoblast cell line (BTK−), were chosen for our cellular experiments. Cells were incubated with increasing concentrations of Evo-1 and Evo-2, at 37 °C for 2 h, followed by washing in probe-free media for 16 h. Flow cytometry analysis was carried out before and after the washout. To confirm that the fluorescence signal originated from the probes bound inside the cell and not the fluorophore unit alone, a control set of cells was incubated with the Boc-protected BODIPY-FL-1 (compound 2). As expected, the fluorescence signal from the BODIPY control was only slightly higher than that of the DMSO, because the fluorophore unit, although it cannot bind covalently, is not totally removed during the washout. However, both cell lines were labelled in a dose-dependent manner even after the washout step (Fig. 4(a) and Fig. S10–S13, ESI†). The mean fluorescence intensities (MFIs) were ranked as Evo-1 > Evo-2, correlating with the lower reactivity of the tertiary acrylamide warhead of Evo-2. After the washout process, the MFIs decreased, and this trend was notably more pronounced at higher concentrations. This decrease is likely due to the removal of non-covalently bound probes. However, potential reductions due to cell division or degradation of BTK and the probe by cellular metabolism, cannot be excluded over the 16 h period. Western blot analysis of Ramos and Jurkat cell lysates revealed that no BTK was expressed in the Jurkat cells (Fig. S14, ESI†) and that the fluorescence detection from the flow cytometry experiments resulted from off-target labelling. The selectivity profiles of the two probes at 500 nM were investigated by in-gel fluorescence (Fig. 4(b)). Although both probes exhibited off-target labelling in both lysates, this effect was significantly more evident for Evo-1. This is likely due to the reduced reactivity of the tertiary acrylamide warhead in Evo-2. Western blot experiments with fluorescent antibodies confirmed that the band at ∼75 kDa corresponded to BTK (Fig. S14, ESI†).

Immunoprecipitation demonstrated that the probe was bound to cellular BTK (Fig. 4(c) and (d), S15, ESI†). Fluorescence detection further confirmed that BTK remained fluorescent after immunoprecipitation (Fig. 4(c)); the latter process was verified by chemiluminescence detection (Fig. 4(d)). The toxicity of the probes was also assessed in Ramos cells, using the trypan blue exclusion test. In both cases, the probes had no significant effect on cellular proliferation at concentrations of up to 10 μM (Fig. S16, ESI†), indicating no cytotoxicity to Ramos cells. Based on the results of the cellular labelling experiments, we chose Evo-2 for further studies. Labelling of endogenous BTK in Ramos cells using different concentrations of Evo-2 (Fig. 4(e)), indicated that 1 μM of the probe yielded an adequate signal-to-noise ratio, without excessive off-target labelling. Nonetheless, the compound exhibited high selectivity for another protein of lower MW even at low concentrations. Immunoblotting experiments suggested that this band corresponded to SLC25A20, a mitochondrial fatty-acid-transporting protein (Fig. S17, ESI†). Furthermore, this band did not disappear when Ramos cells were pretreated with ibrutinib before probe incubation (Fig. S18, ESI†), compared to the BTK band.

To address whether the BTK cellular activity is affected after probe binding, we investigated BTK's autophosphorylation in Ramos cells. BTK has two distinct phosphorylation sites, a transphosphorylation site (Tyr551) in the activation loop and an autophosphorylation site (Tyr223) located in the SH3 domain of BTK. Phosphorylation at both sites regulates BTK's catalytic activity and its endogenous localisation.^36^ B-cell activation triggers the phosphorylation of Tyr551 by Src family kinases such as Lyn and Syk,^17,37,38^ whereas subsequent phosphorylation of Tyr223 stabilises the active conformation, activating BTK.^39^ Phosphorylation of BTK at Tyr223 increased substantially upon BCR-stimulation with anti-human IgM, while pretreatment with ibrutinib completely abolished the autophosphorylation of BTK (Fig. 4(f)). Thus, in agreement with the findings of our biochemical assay, Evo-2 does not interfere with the enzyme's cellular activity.

### Fluorescence microscopy

**Fig. 5:**
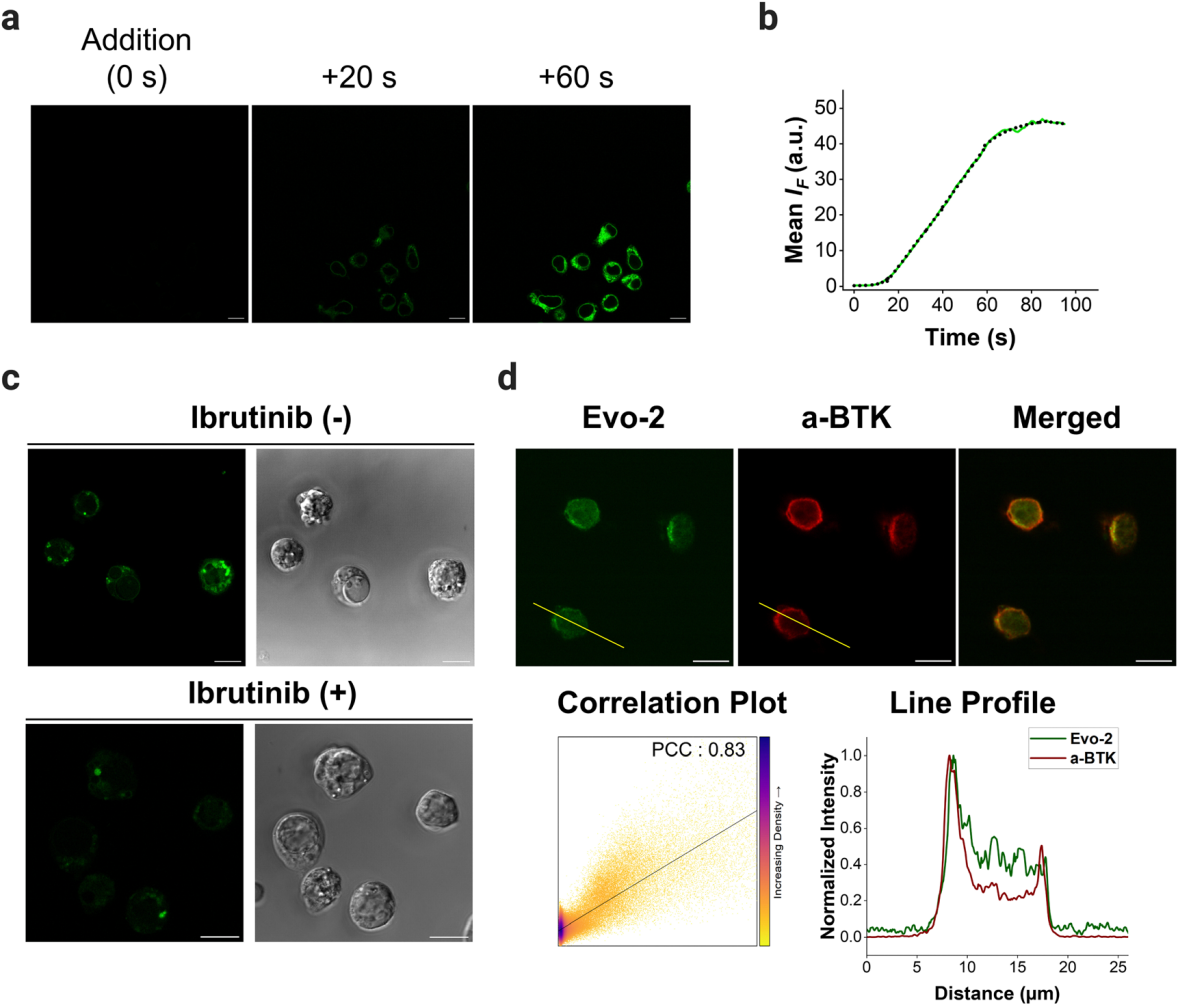
Live-cell imaging of Ramos cells with Evo-2. (a) Confocal microscopy images of Ramos cells showing the rapid cellular uptake of Evo-2 (500 nM; green, *λ*_exc_ = 488 nm). (b) Changes in fluorescence emission upon excitation at 488 nm during the cellular uptake of Evo-2. The dotted line serves as a visual guide. (c) Competition experiments with ibrutinib. Ramos cells were pretreated with or without ibrutinib (5 μM) for 30 min. The unwashed cells were incubated with Evo-2 (1 μM; green, *λ*_exc_ = 488 nm) for 2 h. The corresponding bright-field images are also shown. (d) Co-localisation studies of Evo-2 and cellular BTK. Cells were incubated with 1 μM Evo-2 for 2 h (green, *λ*_exc_ = 488 nm) before staining with mouse anti-BTK antibody, followed by goat anti-mouse Alexa 633 antibody (red, *λ*_exc_ = 633 nm). The correlation plot with the PCC value and the emission intensity profile plot along the yellow line for the two channels under study are shown. Scale bars: 10 μm.

To demonstrate the potential of Evo-2 as a tool for labelling endogenous BTK, we used confocal microscopy to visualise the cellular uptake and subcellular location of Evo-2 in Ramos cells. The imaging experiments revealed that the probe permeabilised the cell membrane rapidly and localised in the cytosol in the absence of cell stimulation (Fig. 5(a), and Supplementary Movie 1, ESI†). The fluorescence reached an intensity plateau approximately 60 s after the addition (Fig. 5(b)). In contrast, negative control cells treated with only DMSO displayed only minor background fluorescence (Fig. S19, ESI†), indicating that most of the observed fluorescence signal is related to Evo-2.

**Fig. 6:**
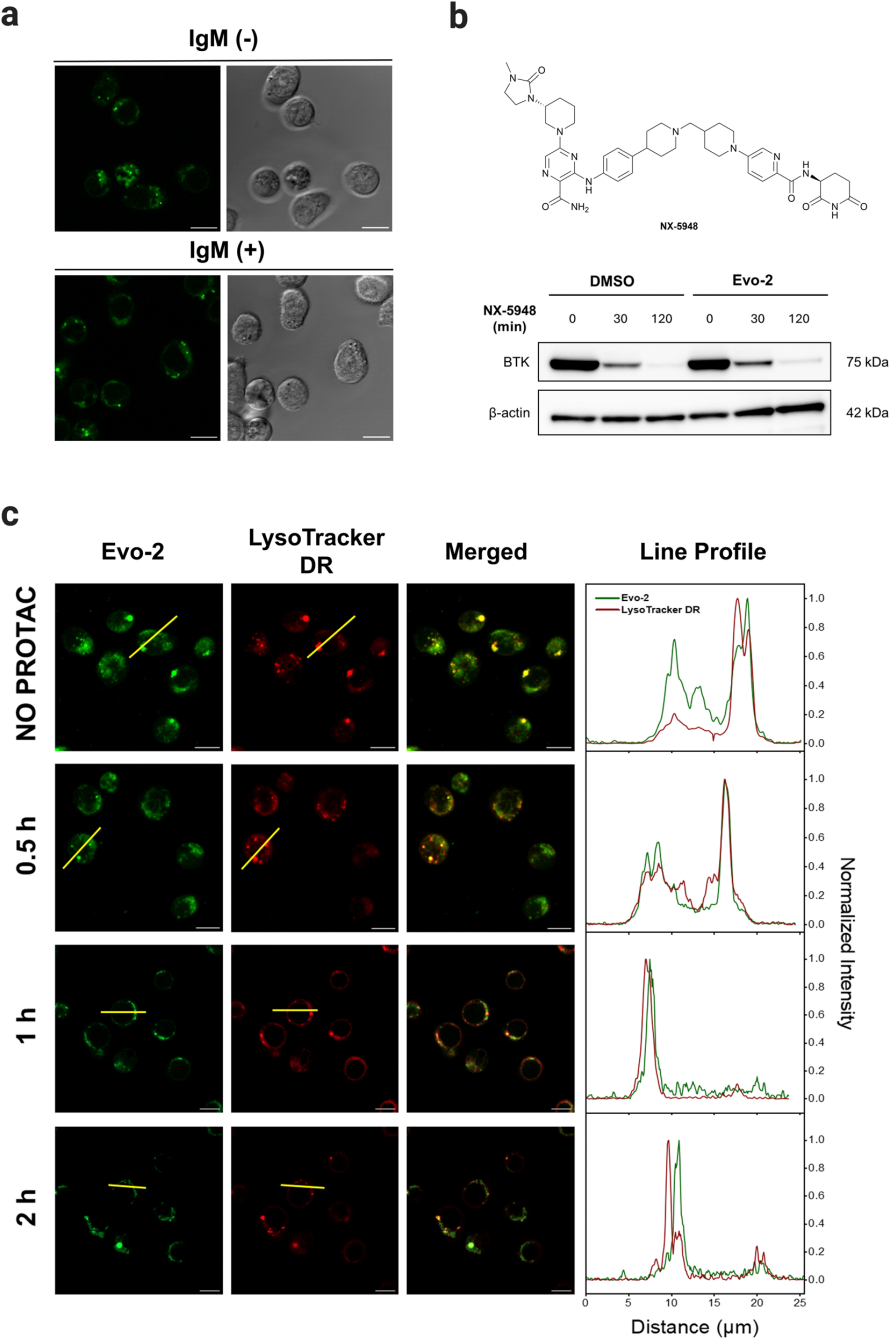
Trafficking of Evo-2-labelled BTK. (a) Translocation stimulation experiments with anti-human IgM (10 μg μL^−1^). Ramos cells were incubated with Evo-2 (1 μM; green, *λ*_exc_ = 488 nm) for 2 h, and then exposed or not to the presence of anti-human IgM (10 μg μL^−1^) for 10 min. The corresponding bright-field images are also presented. (b) The effect of Evo-2 on PROTAC NX-5948-induced degradation of cellular BTK as assessed by western blotting. Ramos cells were incubated with 1 μM Evo-2 for 2 h, and after washing, cells were treated with 10 nM NX-5948 for 0-, 30-, and 120-min. Samples were then lysed and analysed by western blot using anti-BTK and anti-β actin antibodies. (c) The degradation effect of Evo-2-labelled-BTK by PROTAC NX-5948 as determined by confocal microscopy. Fluorescence images of Evo-2 (green, *λ*_exc_ = 488 nm), LysoTracker DeepRed (red, *λ*_exc_ = 639 nm), and the emission intensity profile plots along the yellow line for the two channels under study. Ramos cells were incubated with 1 μM Evo-2 for 2 h, and after washing, cells were labelled with LysoTracker DeepRed (50 nM) for 30 min followed by treatment with PROTAC NX-5948 (10 nM) for 0.5, 1 and 2 h. Shown is a representative frame from live-cell imaging of cells at the different incubation time-points with the PROTAC. Scale bars: 10 μm.

To assess the ability of Evo-2 for labelling BTK, competition experiments with ibrutinib were conducted (Fig. 5(c)). Treating Ramos cells with excess of ibrutinib (5 μM) resulted in a substantially reduced green fluorescence intensity compared to cells not exposed to the inhibitor. This points towards Evo-2 binding mainly to BTK, agreeing with the visual analysis of the images in Fig. 5(c) and the corrected total cell fluorescence (CTCF) plot (Fig. S20, ESI†). Negative control cells, which were not treated with the probe, exhibited only minimal background fluorescence (Fig. S21, ESI†). These findings were in agreement with our cellular labelling experiments with ibrutinib (Fig. S18, ESI†), further confirming that Evo-2 binds to the same site as ibrutinib. To further confirm that Evo-2 labels endogenous BTK, we performed co-localisation experiments using an anti-BTK antibody. Ramos cells were first incubated with Evo-2 for 2 h before fixation and blocking. In turn, the cells were treated with an anti-BTK antibody (or an isotype control) followed by treatment with a secondary antibody tagged with Alexa 633 which recognizes and binds to the anti-BTK antibody. Composite images of Evo-2 (green) and Alexa 633 (red) are displayed in Fig. 5(d), showing a good overlap between these signals. This overlap was observed in the fluorescence intensity profiles and was verified by the Pearson correlation coefficient (PCC), which was above 0.80. Negative control cells without the addition of Evo-2 exhibited minimal background fluorescence (Fig. S22, ESI†), in agreement with the live-cell imaging results. Moreover, no fluorescence signal was observed in the isotype control cells (Fig. S23 and S24, ESI†), confirming that fluorescence originates from specific anti-BTK antibody staining and not from unspecific binding to other proteins. Previous studies demonstrated that BTK translocates to the plasma membrane, upon stimulation of BCR with anti-IgM antibodies.^20^ To elucidate if the probe affects the subcellular distribution of BTK upon BCR stimulation, Ramos cells were incubated with 1 μM Evo-2 in the presence or absence of anti-IgM antibodies. When the cells were stimulated with anti-human IgM, the strong fluorescence signal of the probe appeared to accumulate along the cell membrane. In contrast, the fluorescence signal of the non-stimulated cells was primarily observed in the cytoplasm (Fig. 6(a)). These results demonstrate that binding of Evo-2 to BTK does not affect the translocation of the enzyme to the cell membrane upon BCR stimulation. However, some fluorescent punctuated patterns were also detected together with the cytoplasmic and membrane signals in each case. This off-target labelling may be associated with an unspecific accumulation of Evo-2 in some small subcellular vesicles such as endosomes or lysosomes. Lysosomes, the most acidic compartments within the cell (pH ∼ 5), facilitate the accumulation of weakly basic molecules.^40^ The incorporation of lipophilic amines, such as morpholine and piperazine, has been extensively utilized in the generation of lysosome-targeting probes due to their protonation at lower pH. The protonated amines in lysosomes are membrane impermeable, which results in the lysosomal/endosomal selective entrapment of the probes.^41^ We assumed that the intrinsic basicity of the piperidine nitrogen (p*K*_a_ 11.1) would cause lysosomal accumulation of Evo-2 due to the protonation effect. To validate this hypothesis, Evo-2-treated Ramos cells were co-incubated with a lysosome-specific probe (LysoTracker DeepRed). As expected, the bright green fluorescence detected previously in discrete cytoplasmic regions, co-localised with the lysosome-specific probe (Fig. S25, ESI†), thus confirming our hypothesis that Evo-2 exerts a lysosome-anchoring activity. Furthermore, negative control cells without the addition of the probe showed minimal background fluorescence (Fig. S26, ESI†).

As shown above, binding of Evo-2 to BTK does not affect the enzymatic activity or translocation upon activation. This inspired us to investigate whether the targeted degradation of Evo-2-labelled BTK could be tracked in real time using confocal microscopy. Proteolysis targeting chimeras (PROTACs) are a popular modality to induce selective degradation of cellular proteins.^42^NX-5948 is a potent degrader of BTK in primary human B cells (DC_50_ = 0.34 nM), undergoing Phase 1 clinical trials in patients with relapsed or refractory B cell malignancies.^43^

For these studies, we first examined if Evo-2 interferes with the binding of the non-covalent PROTAC NX-5948 (Fig. 6(b)). Ramos cells were incubated with 1 μM Evo-2 for 2 h, and after washing, cells were treated with 10 nM NX-5948 for 30 and 120 min, and BTK degradation was evaluated by western blot. As expected, the fluorescent tag did not interfere with the ternary complex formation and the levels of BTK decreased rapidly even during the first 30 minutes of treatment. With these data in hand, we next sought to monitor the time-dependent degradation of BTK in live cells by confocal microscopy (Fig. 6(c)). Ramos cells were incubated with 1 μM Evo-2 for 2 h and after washing, the cells were treated with 10 nM NX-5948 for 0.5, 1, and 2 h.

In the absence of PROTAC, the fluorescent signal was predominantly cytoplasmic, albeit some fluctuating fluorescent patterns co-localised with LysoTracker DeepRed were observed. This is particularly evident in the line profile plot, where, despite the strong correlation between the fluorescent peaks attributed to lysosomes, the signal observed along the cytoplasm in the green channel (Evo-2) is stronger than that of the red (LysoTracker DeepRed). After 30 minutes of treatment with PROTAC, the cytoplasmic fluorescent signal was significantly reduced and after 2 h, the fluorescence co-localised predominantly with lysosomes. These results showcase the potential of Evo-2 as an imaging tool to label endogenous BTK and to study its dynamic signalling pathway in live cells without hampering the enzymatic activity.

## Conclusions

We developed a cell-permeable fluorescent probe for Bruton's tyrosine kinase, based on the evobrutinib scaffold, using ligand-directed chemistry techniques. We proved that the probe binds covalently to BTK without affecting its enzymatic activity in biochemical and cellular assays. Using confocal microscopy, we demonstrated the possibility of employing this probe as an imaging tool to investigate BTK's translocation upon B-cell receptor pathway activation in Ramos cells, as well as its PROTAC-induced degradation. This probe has the potential to advance our understanding of BTK signalling dynamics, encompassing real-time spatial localisation within live cells, without the need for genetic manipulation. Furthermore, this method may apply to other kinases, as more than 200 kinases have an accessible Cys, or Ser, or Lys residue that can be targeted by ATP-competitive covalent kinase inhibitors.^44^ However, careful optimisation is required to avoid off-target labelling and achieve selectivity for each kinase. Hence, this methodology has the potential to expand our knowledge of the roles that kinases play in cell-signalling pathways and may lead to the identification of new therapeutic targets.

## Author contributions

A. P. V. and H. N. wrote the paper with contributions from all authors. M. G. designed the research project. A. P. V. and H. N. performed molecular modelling, synthesised, and characterised all the probes, and conducted the cell-free biochemical assays. C. S. prepared the fluorescent group used for probe synthesis. L. H. and W. S. J. performed the cell-based experiments. A. P. V., H. N., W. S. J., and A. C. carried out the flow cytometry experiments. A. P. V. and C. B.-M. conducted the fluorescence bioimaging experiments. M. G., J. B., and J. A. directed the project.

## Data availability

All data supporting the findings of this research can be found in the main article and the ESI.†

## Conflicts of interest

The authors declare no competing interest.

## Supplementary Material

CB-006-D4CB00313F-s001

CB-006-D4CB00313F-s002

CB-006-D4CB00313F-s003
