# Supplementary material for: Design and application of a fluorescent probe for imaging of endogenous Bruton's tyrosine kinase with preserved enzymatic activity
Source: RSC Chem Biol. 2025 Feb 20;6(4):618–29. doi: 10.1039/d4cb00313f (PMC11867108; doi:10.1039/d4cb00313f)
Supplement: CB-006-D4CB00313F-s002 [file CB-006-D4CB00313F-s002.pdf]

Supplementary Information (SI) for RSC Chemical Biology.
This journal is © The Royal Society of Chemistry 2025

Supporting Information

Design and application of a fluorescent probe for imaging of endogenous Bruton's tyrosine kinase with preserved enzymatic activity

Anna P. Valaka[a†], Hampus Nyström[a†], Liliana Håversen[b], Carlos Benitez-Martin[a], Clara Schäfer[a], Woo Suk Jang[c], Alessandro Camponeschi[c], Joakim Andréasson[d], Jan Borén[b] and Morten Grøtli[a*]

[a]Department of Chemistry and Molecular Biology, University of Gothenburg, 405 30, Gothenburg, Sweden. [b]Department of Molecular and Clinical Medicine, University of Gothenburg and Sahlgrenska University Hospital, 413 45 Gothenburg, Sweden. [c]Department of Rheumatology and Inflammation Research, Institute of Medicine, Sahlgrenska Academy, University of Gothenburg, Gothenburg, 413 46, Sweden. [d]Department of Chemistry and Chemical Engineering, Chalmers University of Technology, 412 96, Gothenburg, Sweden.

*†These authors contributed equally. *Corresponding author.*

Table of Contents

General Information 2
Experimental Section 3
  Molecular Docking 3
  Chemistry 3
  LC-MS/MS measurements 12
  Biochemical Assays 13
  Cellular Assays and confocal microscopy 14
Supplementary Figures and Tables 19
NMR Spectra 48
HRMS Spectra 78
HPLC Chromatograms 81
References 83

General Information

All solvents and reagents were obtained from commercial suppliers, stored as indicated by the suppliers, and used without further purification unless otherwise stated. Ibrutinib was purchased from MedChemExpress. Dry THF and DCM were obtained from a solvent purification system (PS-MD-5/7 Inert technology). Reactions were monitored by TLC, LC-MS, and/or HPLC. ^{1}H NMR and ^{13}C NMR spectra were recorded on a 400 MHz Varian, a 600 MHz Bruker Avance Neo, a 700 MHz Bruker Avance III, or an 800 MHz Bruker Advance III HD spectrometer at 25°C. All chemical shifts (^{1}H, ^{13}C) are reported in parts per million (δ) relative to the residual solvent peak ($CDCl_3$: 7.26 ppm, 77.16 ppm; $(CD_3)_2SO$: 2.50 ppm, 39.52 ppm; $(CD_3)_2CO$: 2.05 ppm, 29.84 ppm; D_2O: 4.79 ppm). The following abbreviations are used to denote the multiplicities: s = singlet, br s = broad singlet, d = doublet, dd = doublet of doublets, t = triplet, td = triplet of doublets, tt = triplet of triplets, q = quartet, qd = quartet of doublets, m = multiplet. Coupling constants (*J*) are reported in Hz.

TLC was conducted on silica-gel-coated aluminium sheets for normal-phase (Merck TLC Silica gel 60 F_{254}) respectively reverse-phase (Merck TLC Silica gel 60 RP-18 F_{254}s) and were visualized by UV light (λ = 254 nm or 366 nm). LC-MS was performed on a Waters Acquity system (Acquity Arc HPLC system; 2489 UV/Vis Detector; XBridge BEH C18 column, 130Å, 2.5 μm, 2.1 x 50 mm; XBridge BEH C18 Guard column, V-Gd Cart 2.5 μm, 2.1 x 5 mm; Acquity QDa Mass Detector; MeCN:water (0.01% formic acid), 40°C).

Analytical HPLC was performed on a Waters system (2690 Separation Module; 996 Photodiode Array Detector; Chromolith SpeedROD RP-18 endcapped 50-4.6 HPLC column; MeCN:water (0.1% TFA)). Preparative HPLC was performed on a Waters system (1525 Binary HPLC Pump; 2998 Photodiode Array Detector; Atlantis Prep T3 OBD column; MeCN:water (0.1% TFA)), by injecting the crude dissolved in MeOH. Column chromatography was performed on a Selekt or Isolera One flash chromatography system (Biotage) for normal-phase or reverse-phase, respectively. The silica gel was Sfär Silica D Duo 60 μm or Sfär KP-Amino D Duo 50 μm cartridges for normal-phase, and Sfär C18 D Duo 100 Å 30 μm cartridges for reverse-phase (Biotage). For all column chromatography, dry loading was performed using the same type of silica as the stationary phase. The reactions that employed microwave irradiation were performed in capped vials using an Initiator+ Microwave Synthesizer (Biotage) with fixed hold time.

HRMS data were recorded with a QExactive HF Orbitrap mass spectrometer interfaced with Dionex Ultimate 3000 liquid chromatography system (Thermo Fisher Scientific). The instrument operated in full MS mode only, where the ion mass spectra were acquired at a resolution of 120 000, maximum injection time 200 ms for $3x10^{6}$ ions. The Orbitrap was calibrated with Pierce LTQ ESI Positive Ion Calibration Solution prior to the analysis, resulting in mass accuracy better than 5 ppm. Electrospray ionization was performed at 4 kV and 320°C using a metal emitter in the ion source. The sample (1 or 10 μL) was injected onto a reversed-phase XBridge BEH C18 column (3.5 μm, 2.1 x 50 mm, Waters). The analysis was performed using a linear gradient over 2.5 min from 10 to 100% solvent B, followed by isocratic eluted with 100% solvent B for 17.5 min with a flow of 0.300 ml/min (solvent A: water with 0.1% formic acid; solvent B: 80% acetonitrile in water with 0.1% formic acid). Data analysis was performed using the Xcalibur software (Thermo Fischer Scientific).

Experimental Section

Molecular Docking

Molecular docking was conducted using the Molecular Operating Environment (MOE) version 2020.0901 (Chemical Computing Group, Montreal, CA). The structure of evobrutinib co-crystallised with BTK was obtained from the Protein Data Bank (PDB ID: 6OMU). The ligand-receptor complex was prepared with the automated QuickPrep script, which added hydrogen atoms, adjusted protonation states to physiological pH, and refined the structure. QuickPrep used the Amber10:EHT force field. Ligands were built in MOE, with protonation states to physiological pH and energy minimization was preformed using the MMFF94x force field. Docking employed the AMBER10:EHT force field with the Dock Template function, using the evobrutinib-amine (compound **9**) fragment as a template. Settings included rotatable bonds for ligands, a rigid receptor, the GBVI/WSA dG score function, 1000 placement poses, and 100 refinement poses. The top 10 poses were ranked by their scores, and evaluated based on the warhead orientation, and the distance to Cys481.

Chemistry

3-(5,5-difluoro-7,9-dimethyl-5*H*-4λ[4],5λ[4]-dipyrrolo[1,2-*c*:2',1'-*f*][1,3,2] diazaborinin-3-yl)propanoic acid (1)

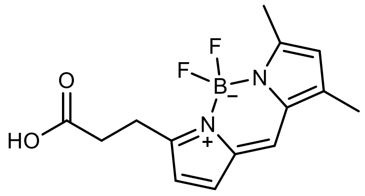

1

Compound **1** was synthesised according to the literature procedure[1].
^{1}H NMR (600 MHz, $CDCl_3$) δ 7.09 (s, 1H), 6.89 (d, *J* = 4.0 Hz, 1H), 6.29 (d, *J* = 4.0 Hz, 1H), 6.12 (s, 1H), 3.30 (t, *J* = 7.6 Hz, 2H), 2.83 (t, *J* = 7.6 Hz, 2H), 2.57 (s, 3H), 2.25 (s, 3H); ^{13}C NMR (151 MHz, $CDCl_3$) δ 176.0, 160.8, 156.5, 144.0, 133.3, 128.0, 123.9, 120.6, 116.7, 32.7, 23.7, 15.0, 11.3; LC-MS (ESI) *m/z*: $[M+H]^+$ calcd. for $C_{14}H_{16}BF_2N_2O_2$: 293.13, found: 293.09.

***tert*-butyl (2-(3-(5,5-difluoro-7,9-dimethyl-5*H*-4λ[4],5λ[4]-dipyrrolo[1,2-*c*:2',1'-*f*][1,3,2]diazaborinin-3-yl)propanamido)ethyl)carbamate (2)**

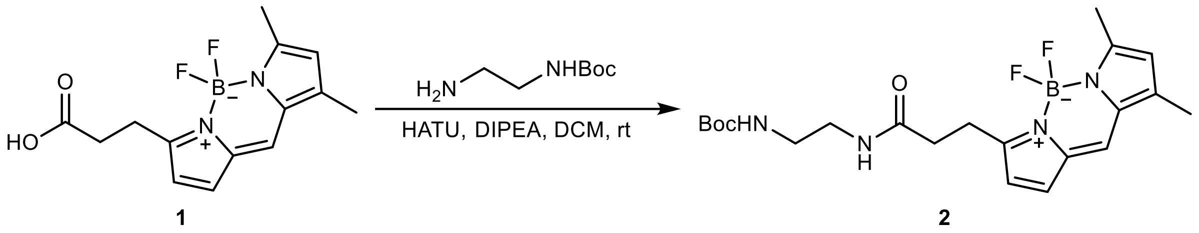

A 100 ml round-bottom flask was charged with **1** (202 mg, 0.69 mmol, 1.0 eq.) and HATU (315 mg, 0.83 mmol, 1.2 eq.), and the flask was purged with N_2. Then, dry DCM (8 ml), DIPEA (0.14 ml, 0.83 mmol, 1.2 eq.), and *N*-Boc-ethylenediamine (0.13 ml, 0.83 mmol, 1.2 eq.) as a solution in dry DCM (2 ml), were added in rapid succession. The orange solution was stirred at room temperature. After 40 min, LC-MS indicated full consumption of the starting material, and the reaction mixture was concentrated under reduced pressure. The residual red oil was dissolved in EtOAc (75 ml), washed with saturated aqueous NH_4Cl (3x50 ml) and water (50 ml), and concentrated under reduced pressure. The crude was purified by column chromatography (silica 10 g, 0–20% MeOH:DCM) to afford **2** (eluted at 10% MeOH) as a red solid (282 mg, 94%).

^{1}H NMR (400 MHz, $(CD_3)_2CO$) δ 7.51 (s, 1H), 7.28 (br s, 1H), 7.03 (d, *J* = 4.1 Hz, 1H), 6.37 (d, *J* = 4.1 Hz, 1H), 6.25 (s, 1H), 6.09 (br s, 1H), 3.30 (q, *J* = 6.0 Hz, 2H), 3.23 (t, *J* = 7.7 Hz, 2H), 3.17 (q, *J* = 5.9 Hz, 2H), 2.59 (t, *J* = 7.7 Hz, 2H), 2.51 (s, 3H), 2.28 (s, 3H), 1.40 (s, 9H); ^{13}C NMR (101 MHz, $(CD_3)_2CO$) δ 172.3, 160.2, 159.3, 156.9, 144.9, 135.8, 134.4, 129.5, 125.6, 121.0, 117.7, 78.7, 41.2, 40.3, 35.4, 28.6, 25.1, 14.8, 11.2; LC-MS (ESI) *m/z*: $[M+Na]^+$ calcd. for $C_{21}H_{29}BF_2N_4NaO_3$: 457.22, found: 457.32.

tert-butyl 4-(3-(5,5-difluoro-7,9-dimethyl-5*H*-4λ⁴,5λ⁴-dipyrrolo[1,2-*c*:2',1'-*f*][1,3,2]diazaborinin-3-yl)propanamido)piperidine-1-carboxylate (3)

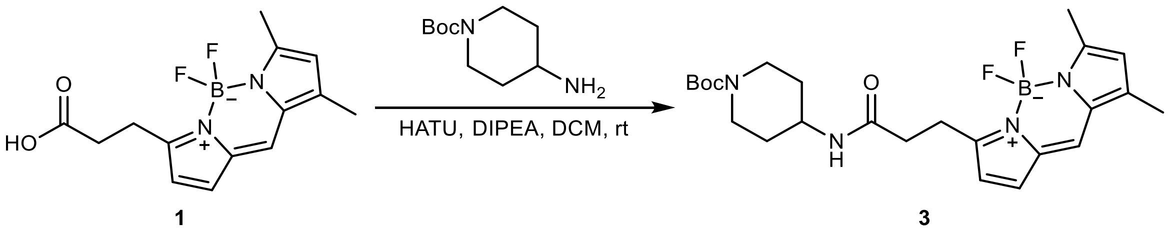

A 50 ml round-bottom flask was charged with **1** (76 mg, 0.26 mmol, 1.0 eq.) and HATU (119 mg, 0.31 mmol, 1.2 eq.), and the flask was purged with N_2. Then, dry DCM (4 ml), DIPEA (54 µL, 0.31 mmol, 1.2 eq.), and 4-amino-1-Boc-piperidine (63 mg, 0.31 mmol, 1.2 eq.) as a solution in dry DCM (2 ml), were added in rapid succession. The orange solution was stirred at room temperature. After 30 min, LC-MS indicated full consumption of the starting material, and the reaction mixture was concentrated under reduced pressure. The residual red oil was dissolved in EtOAc (40 ml), washed with saturated aqueous NH_4Cl (3x40 ml) and water (40 ml), and concentrated under reduced pressure. The crude was purified by column chromatography (silica 10 g, 0–20% MeOH:DCM) to afford **3** (eluted at 15% MeOH) as a red solid (109 mg, 88%).

^{1}H NMR (600 MHz, $(CD_3)_2CO$) δ 7.50 (s, 1H), 7.11 (d, *J* = 7.7 Hz, 1H), 7.03 (d, *J* = 4.0 Hz, 1H), 6.35 (d, *J* = 4.0 Hz, 1H), 6.24 (s, 1H), 3.99-3.91 (m, 2H), 3.91-3.84 (m, 1H), 3.23 (t, *J* = 7.6 Hz, 2H), 2.96-2.79 (m, 2H), 2.56 (t, *J* = 7.6 Hz, 2H), 2.51 (s, 3H), 2.28 (s, 3H), 1.84-1.79 (m, 2H), 1.42 (s, 9H), 1.31 (qd, *J* = 11.7, 4.1 Hz, 2H);

^{13}C NMR (151 MHz, $(CD_3)_2CO$) δ 171.0, 160.2, 159.3, 155.0, 144.9, 135.8, 134.4, 129.5, 125.6, 121.0, 117.7, 79.4, 47.2, 43.8, 43.0, 35.5, 32.6, 28.5, 25.1, 14.8, 11.2; LC-MS (ESI) *m/z*: $[M+Na]^+$ calcd. for $C_{24}H_{33}BF_2N_4NaO_3$: 497.25, found: 497.39.

2-(3-(5,5-difluoro-7,9-dimethyl-5*H*-4λ⁴,5λ⁴-dipyrrolo[1,2-*c*:2',1'-*f*][1,3,2]diazaborinin-3-yl)propanamido)ethan-1-aminium chloride (BODIPY-FL-1)

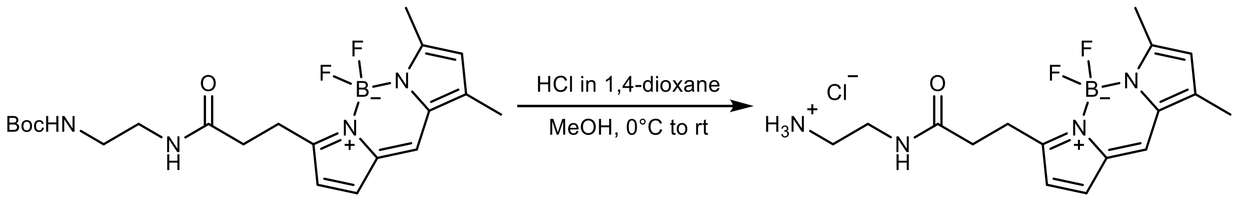

2 **BODIPY-FL-1**

In a 25 ml round-bottom flask, **2** (62 mg, 0.14 mmol, 1.0 eq.) was dissolved in MeOH (5 ml), cooled on ice, and purged with N_2. HCl 4M in 1,4-dioxane (0.34 ml, 1.4 mmol, 10 eq.) was added dropwise and the red solution was stirred at room temperature. After 23 h, LC-MS indicated full consumption of the starting material, and the solution was concentrated under reduced pressure. The residual red oil was taken up in water (10 ml) and washed with EtOAc (10 ml). The aqueous layer was concentrated and dried under reduced pressure to afford **BODIPY-FL-1** as a red oil (53 mg, 100%), that was used in the next step without further purification.

^1H NMR (600 MHz, D_2O) δ 7.43-7.34 (m, 3H), 7.00 (d, *J* = 4.0 Hz, 1H), 6.51 (d, *J* = 4.2 Hz, 1H), 6.40 (s, 1H), 6.33 (d, *J* = 4.0 Hz, 1H), 6.26 (s, 1H), 3.51-3.45 (m, 4H), 3.17-3.07 (m, 8H), 2.73 (t, *J* = 7.3 Hz, 2H), 2.69 (t, *J* = 7.4 Hz, 2H), 2.48 (s, 3H), 2.47 (s, 3H), 2.30 (s, 3H), 2.21 (s, 3H); ^{13}C NMR (151 MHz, D_2O) δ 176.0, 175.4, 161.6, 159.9, 155.4, 150.7, 146.5, 135.4, 133.0, 128.5, 128.1, 125.7, 124.6, 121.0, 119.2, 116.3, 39.1, 39.0, 36.7, 34.4, 33.8, 24.0, 23.6, 14.1, 13.8, 11.2, 10.4; LC-MS (ESI) *m/z*: $[M+H]^+$ calcd. for $C_{16}H_{22}BF_2N_4O$: 335.18, found: 335.27.

Note: the NMR spectra of **BODIPY-FL-1** in D_2O contained two sets of peaks, believed to be due to an equilibrium between protonated and neutral amine. All signals for the pair are listed above. The compound was homogenous in LC-MS analysis.

4-(3-(5,5-difluoro-7,9-dimethyl-5*H*-4λ⁴,5λ⁴-dipyrrolo[1,2-*c*:2',1'-*f*][1,3,2]diazaborinin-3-yl)propanamido)piperidin-1-ium chloride (BODIPY-FL-2)

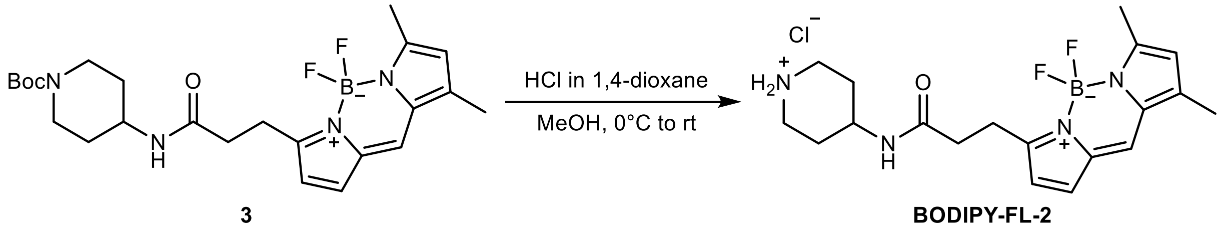

3 **BODIPY-FL-2**

In a 25 ml round-bottom flask, **3** (30 mg, 0.063 mmol, 1.0 eq.) was dissolved in MeOH (3 ml), cooled on ice, and purged with N_2. HCl 4M in 1,4-dioxane (0.15 ml, 0.63 mmol, 10 eq.) was added dropwise and the orange solution was stirred at room temperature. After 24 h, LC-MS indicated full consumption of the starting material, and the solution was concentrated under reduced pressure. The residual red oil was taken up in water (10 ml) and washed with EtOAc (10 ml). The aqueous layer was concentrated and dried under reduced pressure to afford **BODIPY-FL-2** as a red oil (26 mg, 100%), that was used in the next step without further purification.

^{1}H NMR (600 MHz, D_2O) δ 7.26 (s, 1H), 6.92 (d, *J* = 4.0 Hz, 1H), 6.26 (d, *J* = 4.0 Hz, 1H), 6.18 (s, 1H), 3.93-3.86 (m, 1H), 3.43-3.36 (m, 2H), 3.12-3.04 (m, 4H), 2.61 (t, *J* = 7.4 Hz, 2H), 2.42 (s, 3H), 2.14 (s, 3H), 2.07-2.00 (m, 2H), 1.69-1.60 (m, 2H); ^{13}C NMR (151 MHz, D_2O) δ 174.3, 161.3, 155.3, 146.2, 135.2, 133.0, 128.5, 124.5, 120.9, 116.4, 44.1, 42.6, 34.4, 27.6, 24.2, 14.1, 10.4; LC-MS (ESI) *m/z:* $[M+H]^+$ calcd. for $C_{19}H_{26}BF_2N_4O$: 375.22, found: 375.29.

tert-butyl 4-(((6-amino-5-chloropyrimidin-4-yl)amino)methyl)piperidine-1-carboxylate (6)

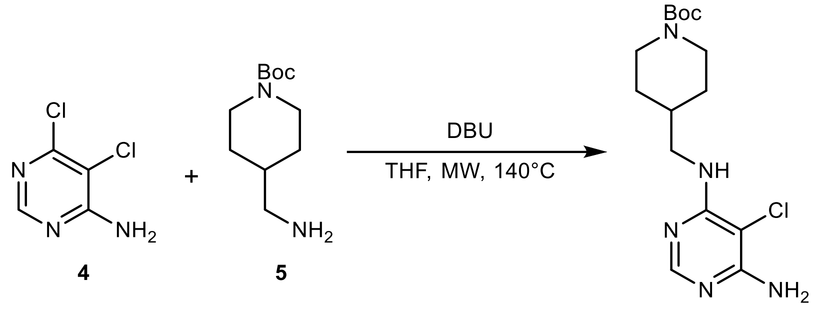

4 **5** **6**

Compound **6** was synthesised using a modified literature procedure[2]:
An oven-dried 20 ml microwave vial was charged with **4** (1.00 g, 6.11 mmol, 1.0 eq.), **5** (1.42 ml, 6.72 mmol, 1.1 eq.), DBU (1.82 ml, 12.2 mmol, 2.0 eq.), and dry THF (8 ml), and purged with N_2. The reaction mixture was irradiated at 140°C in the microwave reactor for 4 h, until reversed-phase TLC (MeCN:water 4:1; R_f(**4**) = 0.79; R_f(**6**) = 0.46) indicated full consumption of the starting material. The white solids were filtered, and the orange filtrate was concentrated under reduced pressure. The residual orange oil was dissolved in EtOAc, washed once with water, three times with saturated aqueous NH_4Cl, and once with brine. The organic layer was concentrated under reduced pressure and dried under high vacuum to afford a pale orange solid (1.47 g, 70%).
^{1}H NMR (600 MHz, $(CD_3)_2SO$) δ 7.83 (s, 1H), 6.68 (t, J = 6.0 Hz, 1H), 6.43 (br s, 2H), 3.90 (s, 2H), 3.21 (t, J = 6.5 Hz, 2H), 2.64 (br s, 2H), 1.79-1.70 (m, 1H), 1.59 (d, J = 13.4 Hz, 2H), 1.38 (s, 9H), 0.97 (qd, J = 12.2, 4.2 Hz, 2H); ^{13}C NMR (201 MHz, $(CD_3)_2SO$) δ 158.3, 157.3, 154.8, 153.9, 90.3, 78.4, 45.6, 43.7, 42.7, 35.6, 29.5, 28.1; LC-MS (ESI) m/z: $[M+H]^+$ calcd. for $C_{15}H_{25}ClN_5O_2$: 342.17, found: 342.19.

tert-butyl 4-(((6-amino-5-(4-phenoxyphenyl)pyrimidin-4-yl)amino)methyl)piperidine-1-carboxylate (7)

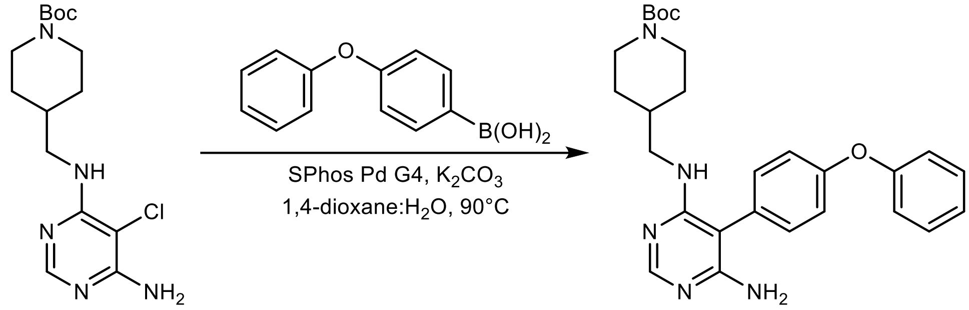

6 **7**

Compound **7** was synthesised using a modified literature procedure[2]:
A 20 ml microwave vial was charged with **6** (506 mg, 1.48 mmol, 1.0 eq.), K_2CO_3 (409 mg, 2.96 mmol, 2.0 eq.), 4-phenoxyphenylboronic acid (475 mg, 2.22 mmol, 1.5 eq.), SPhos Pd G4 (29.4 mg, 0.037 mmol, 2.5 mol%), and purged with argon. 1,4-dioxane (10 ml) degassed with argon, and H_2O (2.5 ml), were added and the solution was heated to 90°C, upon it changed colour from yellow to orange. After 20 h, LC-MS indicated

full consumption of the starting material. The reaction mixture was filtered on celite, and the filtrate was concentrated under reduced pressure. The crude was triturated with EtOAc multiple times, until no more precipitate was formed, to afford **7** as a white solid (533 mg, 76%).

^{1}H NMR (700 MHz, $(CD_3)_2SO$) δ 7.94 (s, 1H), 7.42 (t, *J* = 7.7 Hz, 2H), 7.21 (d, *J* = 8.5 Hz, 2H), 7.16 (tt, *J* = 7.4, 1.1 Hz, 1H), 7.13-7.09 (m, 4H), 5.48-5.39 (m, 3H), 3.93-3.85 (m, 2H), 3.12 (t, *J* = 6.6 Hz, 2H), 2.73-2.55 (m, 2H), 1.75-1.68 (m, 1H), 1.56-1.50 (m, 2H), 0.90 (qd, *J* = 12.1, 4.4 Hz, 2H); ^{13}C NMR (151 MHz, $(CD_3)_2SO$) δ 160.0, 159.5, 156.6, 156.3, 156.0, 153.8, 132.2, 130.0, 128.1, 123.5, 119.9, 118.8, 96.1, 78.4, 45.5, 43.7, 42.8, 35.5, 29.4, 28.1; LC-MS (ESI) *m/z*: $[M+H]^+$ calcd. for $C_{27}H_{34}N_5O_3$: 476.27, found: 476.35.

4-(((6-amino-5-(4-phenoxyphenyl)pyrimidin-4-yl)amino)methyl)piperidin-1-ium chloride (8)

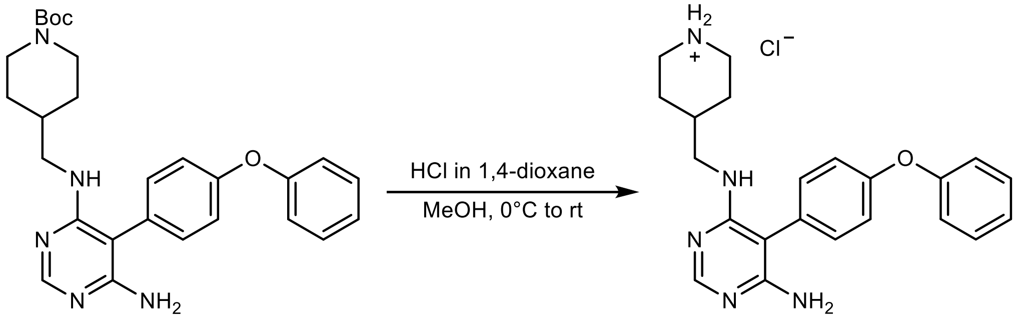

7 **8**

Compound **8** was synthesised using a modified literature procedure[2]:

In a 100 ml round-bottom flask, **7** (635 mg, 1.34 mmol, 1.0 eq.) was suspended in MeOH (25 ml), cooled on ice, and purged with N_2. HCl 4M in 1,4-dioxane (3.20 ml, 13.4 mmol, 10 eq.) was added dropwise and the white suspension was stirred at room temperature. After 18 h, LC-MS indicated full consumption of the starting material. The mixture was concentrated under reduced pressure to afford **8** as a pale yellow crystalline solid (550 mg, 100%), that was used in the next step without further purification.

LC-MS (ESI) *m/z*: $[M+H]^+$ calcd. for $C_{22}H_{26}N_5O$: 376.21, found: 376.35.

Note: The crude salt **8** performed well in the amide coupling to form evobrutinib (**10**) by basification *in situ* with additional equivalents of DIPEA. However, in the *N*-alkylation with 2-(bromomethyl)acrylic acid to form **11**, the free amine **9** in high purity performed better. The basification and purification procedures are described below.

5-(4-phenoxyphenyl)-N^4-(piperidin-4-ylmethyl)pyrimidine-4,6-diamine (9)

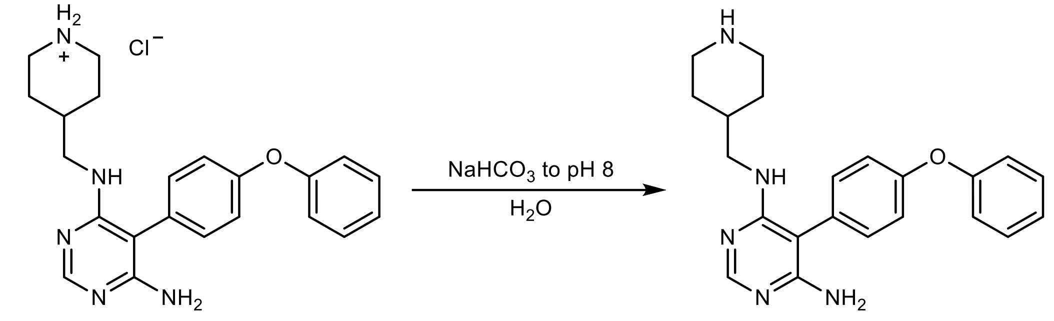

8 **9**

In a 50 ml round-bottom flask, **8** (240 mg, 0.58 mmol, 1.0 eq.) was dissolved in water (5 ml), and the salt was basified to pH 8 by addition of a saturated aqueous $NaHCO_3$ solution. The water was removed under reduced pressure, and the free amine was purified by column chromatography (KP-Amino silica 11 g, 0–100% EtOAc:pentane, then 0–25% MeOH:EtOAc) to afford **9** (eluted at 15% MeOH) as a pale yellow solid (119 mg, 54%).

^{1}H NMR (600 MHz, $(CD_3)_2SO$) δ 7.94 (s, 1H), 7.41 (t, *J* = 7.7 Hz, 2H), 7.21 (d, *J* = 8.1 Hz, 2H), 7.16 (t, *J* = 7.4 Hz, 1H), 7.13-7.09 (m, 4H), 5.41 (s, 2H), 5.29 (t, *J* = 6.2 Hz, 1H), 3.10 (t, *J* = 6.6 Hz, 2H), 2.86 (d, *J* = 12.0 Hz, 2H), 2.34 (t, *J* = 11.9 Hz, 2H), 1.63-1.53 (m, 1H), 1.46 (d, *J* = 12.5 Hz, 2H), 0.91 (qd, *J* = 12.2, 4.0 Hz, 2H); ^{13}C NMR (151 MHz, $(CD_3)_2SO$) δ 160.0, 159.5, 156.6, 156.3, 156.0, 132.2, 130.0, 128.1, 123.5, 120.0, 118.7, 96.0, 46.4, 45.9, 36.2, 30.9; LC-MS (ESI) *m/z*: $[M+H]^+$ calcd. for $C_{22}H_{26}N_5O$: 376.21, found: 376.35.

1-(4-(((6-amino-5-(4-phenoxyphenyl)pyrimidin-4-yl)amino)methyl)piperidin-1-yl)prop-2-en-1-one (10)

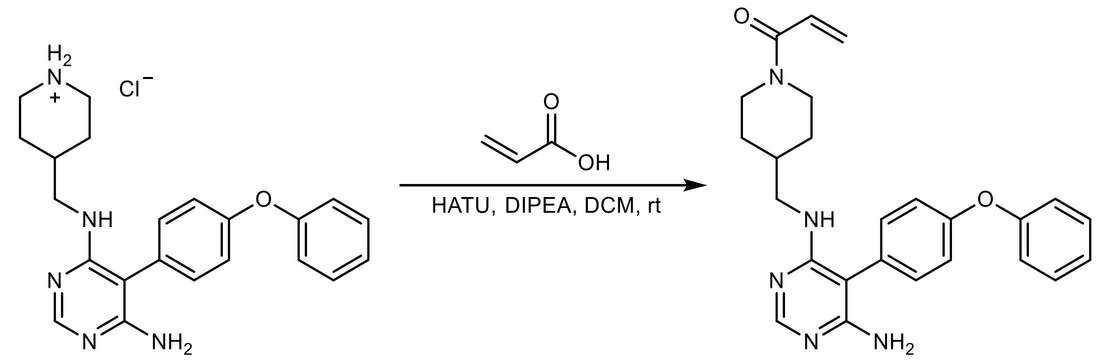

8 **10**

In a 25 ml round-bottom flask purged with N_2, **8** (54 mg, 0.13 mmol, 1.2 eq.) was dissolved in dry DCM (2 ml) and basified by the addition of DIPEA (23 µL, 0.13 mmol, 1.2 eq.). The colourless solution was stirred at room temperature for 10 min. A 50 ml round-bottom flask was charged with acrylic acid (8 µL, 0.11 mmol, 1.0 eq.) and HATU (50 mg, 0.13 mmol, 1.2 eq.), and the flask was purged with N_2. Then, dry DCM (4 ml), DIPEA (23 µL, 0.13 mmol, 1.2 eq.), and the amine solution in the other flask, were added in rapid succession. The pale yellow solution was stirred at room temperature. After 20 min, LC-MS indicated full consumption

of the starting material, and the reaction mixture was concentrated under reduced pressure. The crude was purified by reversed-phase column chromatography (C18 silica 12 g, 0–100% MeCN:water) to afford **10** (eluted at 70% MeCN) as a white solid (16 mg, 35%).

^{1}H NMR (600 MHz, $(CD_3)_2SO$) δ 7.95 (s, 1H), 7.42 (t, *J* = 7.9 Hz, 2H), 7.21 (d, *J* = 8.4 Hz, 2H), 7.16 (t, *J* = 7.4 Hz, 1H), 7.13-7.09 (m, 4H), 6.77 (dd, *J* = 16.7, 10.5 Hz, 1H), 6.05 (dd, *J* = 16.7, 2.4 Hz, 1H), 5.62 (dd, *J* = 10.4, 2.4 Hz, 1H), 5.50-5.39 (m, 3H), 4.35 (d, *J* = 13.0 Hz, 1H), 3.99 (d, *J* = 13.6 Hz, 1H), 3.13 (t, *J* = 6.6 Hz, 2H), 2.96 (t, *J* = 12.1 Hz, 1H), 2.57 (t, *J* = 11.2 Hz, 1H), 1.87-1.78 (m, 1H), 1.60 (t, *J* = 12.7 Hz, 2H), 1.00-0.87 (m, 2H); ^{13}C NMR (151 MHz, $(CD_3)_2SO$) δ 164.1, 159.9, 159.5, 156.6, 156.2, 156.1, 132.3, 130.0, 128.6, 128.0, 126.8, 123.5, 119.9, 118.8, 96.1, 45.4, 45.0, 41.4, 35.7, 30.4, 29.3; HRMS (ESI) *m/z*: $[M+H]^+$ calcd. for $C_{25}H_{28}N_5O_2$: 430.2238, found: 430.2222. Characterisation data was consistent with the literature reference[2].

2-((4-(((6-amino-5-(4-phenoxyphenyl)pyrimidin-4-yl)amino)methyl)piperidin-1-yl)methyl)acrylic acid (11)

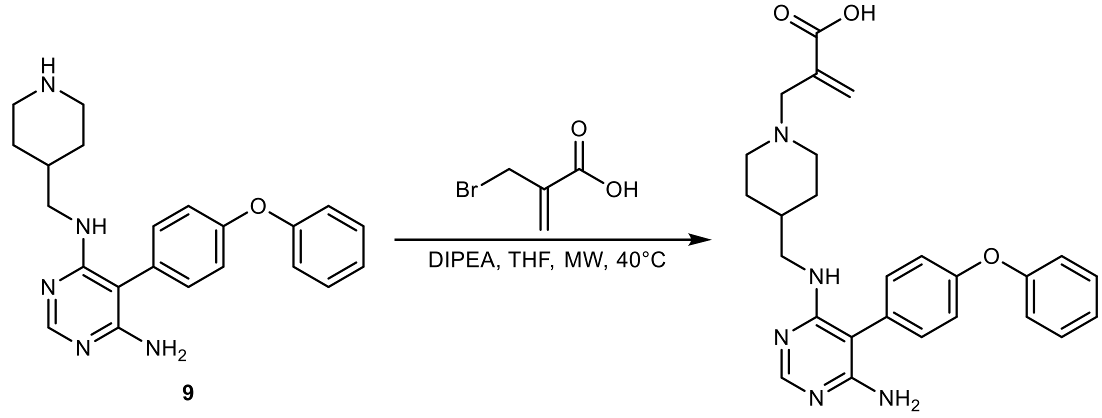

Compound **11** was synthesised using a modified literature procedure[3]:

A 5 ml microwave vial was charged with **9** (80 mg, 0.21 mmol, 1.0 eq.), 2-(bromomethyl)acrylic acid (32 mg, 0.19 mmol, 0.9 eq.), dry THF (3 ml), and purged with N_2. DIPEA (37 µL, 0.21 mmol, 1.0 eq.) was added and the reaction mixture was irradiated at 40°C in the microwave reactor for 15 min. After this time, HPLC indicated full consumption of the starting material, and the reaction mixture was concentrated under reduced pressure. The crude was purified by reversed-phase column chromatography (C18 silica 12 g, 0–100% MeCN:water) to afford **11** (eluted at 40% MeCN) as a white crystalline solid (61 mg, 62%).

^{1}H NMR (600 MHz, $(CD_3)_2SO$) δ 7.94 (s, 1H), 7.41 (t, *J* = 7.8 Hz, 2H), 7.22 (d, *J* = 8.6 Hz, 2H), 7.16 (t, *J* = 7.4 Hz, 1H), 7.13-7.09 (m, 4H), 6.03 (s, 1H), 5.55 (s, 1H), 5.47-5.39 (m, 3H), 3.34 (s, 2H), 3.14 (t, *J* = 6.4 Hz, 2H), 2.97-2.92 (m, 2H), 2.27-2.19 (m, 2H), 1.69-1.60 (m, 3H), 1.16-1.06 (m, 2H); ^{13}C NMR (176 MHz, $(CD_3)_2SO$) δ 168.1, 160.1, 159.5, 156.6, 156.4, 156.1, 136.8, 132.3, 130.1, 128.1, 124.8, 123.6, 120., 118.8, 96.2, 58.5, 51.5, 45.3, 34.5, 28.6; LC-MS (ESI) *m/z*: $[M+H]^+$ calcd. for $C_{26}H_{30}N_5O_3$: 460.23, found: 460.32.

2-((4-(((6-amino-5-(4-phenoxyphenyl)pyrimidin-4-yl)amino)methyl)piperidin-1-yl)methyl)-*N*-(2-(3-(5,5-difluoro-7,9-dimethyl-5*H*-4λ[4],5λ[4]-dipyrrolo[1,2-*c*:2',1'-*f*][1,3,2]diazaborinin-3-yl)propanamido)ethyl)acrylamide (Evo-1)

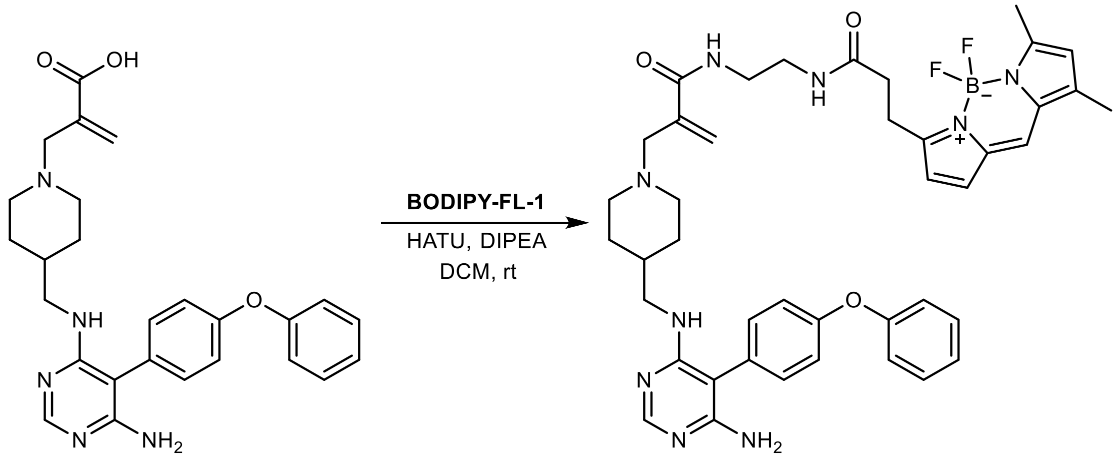

11 **Evo-1**

In a 25 ml round-bottom flask purged with N_2, **BODIPY-FL-1** (51 mg, 0.14 mmol, 1.2 eq.) was dissolved in dry DCM (3 ml) and basified by the addition of DIPEA (24 µL, 0.14 mmol, 1.2 eq.). The red solution was stirred at room temperature for 10 min. A 50 ml round-bottom flask was charged with **11** (52 mg, 0.11 mmol, 1.0 eq.) and HATU (52 mg, 0.14 mmol, 1.2 eq.), and the flask was purged with N_2. Then, dry DCM (5 ml), DIPEA (24 µL, 0.14 mmol, 1.2 eq.), and the amine solution in the other flask, were all added in rapid succession. The red solution was stirred at room temperature. After 45 min, LC-MS indicated full consumption of the starting material, and the reaction mixture was concentrated under reduced pressure. The crude was purified by preparative-HPLC (C18 column, 20-80% MeCN:water with 0.1% TFA), to afford **Evo-1** as a brown solid (38 mg, 43%).

^{1}H NMR (700 MHz, $(CD_3)_2CO$) δ 8.29 (s, 1H), 7.51 (s, 1H), 7.42 (t, *J* = 7.6 Hz, 2H), 7.37 (d, *J* = 8.1 Hz, 2H), 7.19 (tt, *J* = 7.4, 1.1 Hz, 1H), 7.12 (d, *J* = 8.4 Hz, 2H), 7.07 (d, *J* = 7.8 Hz, 2H), 7.03 (d, *J* = 4.0 Hz, 1H), 6.37 (d, *J* = 4.0 Hz, 1H), 6.27 (s, 1H), 6.25 (s, 1H), 6.04 (s, 1H), 4.08 (br s, 2H), 3.66 (d, *J* = 12.1 Hz, 2H), 3.46-3.35 (m, 6H), 3.21 (t, *J* = 7.8 Hz, 2H), 3.02 (t, *J* = 13.0 Hz, 2H), 2.62 (t, *J* = 7.7 Hz, 2H), 2.50 (s, 3H), 2.28 (s, 3H), 2.00-1.95 (m, 2H), 1.64 (q, *J* = 13.5 Hz, 2H); ^{13}C NMR (176 MHz, $(CD_3)_2CO$) δ 173.5, 167.8, 161.5, 160.5, 159.1, 158.9, 157.5, 154.1, 149.3, 145.2, 135.9, 135.0, 134.5, 133.5, 131.0, 129.7, 129.4, 125.8, 124.8, 124.5, 121.2, 121.1, 120.1, 117.5, 96.5, 58.1, 53.2, 46.5, 40.9, 39.1, 35.2, 34.8, 27.9, 25.1, 15.0, 11.3; HRMS (ESI) *m/z*: $[M+H]^+$ calcd. for $C_{42}H_{49}BF_2N_9O_3$: 776.4014, found: 776.3997.

***N*-(1-(2-((4-(((6-amino-5-(4-phenoxyphenyl)pyrimidin-4-yl)amino)methyl)piperidin-1-yl)methyl)acryloyl)piperidin-4-yl)-3-(5,5-difluoro-7,9-dimethyl-5*H*-4λ^4,5λ^4-dipyrrolo[1,2-*c*:2',1'-*f*][1,3,2]diazaborinin-3-yl)propanamide (Evo-2)**

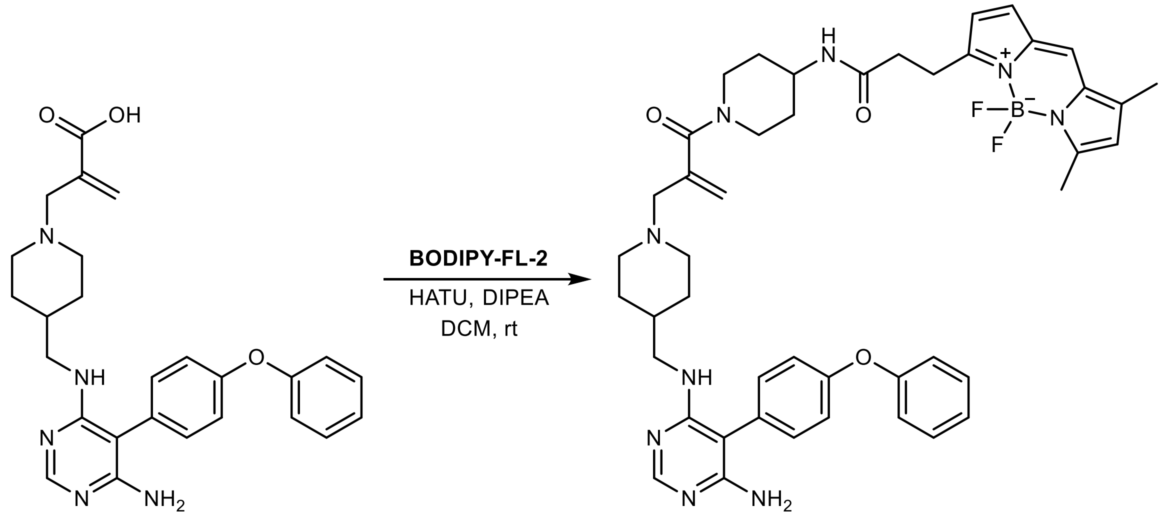

In a 25 ml round-bottom flask purged with N_2, **BODIPY-FL-2** (65 mg, 0.16 mmol, 1.2 eq.) was dissolved in dry DCM (3 ml) and basified by the addition of DIPEA (28 µL, 0.16 mmol, 1.2 eq.). The red solution was stirred at room temperature for 10 min. A 50 ml round-bottom flask was charged with **11** (61 mg, 0.13 mmol, 1.0 eq.) and HATU (61 mg, 0.16 mmol, 1.2 eq.), and the flask was purged with N_2. Then, dry DCM (5 ml), DIPEA (28 µL, 0.16 mmol, 1.2 eq.), and the amine solution in the other flask, were all added in rapid succession. The red solution was stirred at room temperature. After 1 h, LC-MS indicated full consumption of the starting material, and the reaction mixture was concentrated under reduced pressure. The crude was purified by preparative-HPLC (C18 column, 20–80% MeCN:water with 0.1% TFA), to afford **Evo-2** as a black solid (75 mg, 69%).

^{1}H NMR (600 MHz, $(CD_3)_2CO$) δ 8.33 (s, 1H), 7.52 (s, 1H), 7.45-7.39 (m, 4H), 7.19 (t, *J* = 7.4 Hz, 1H), 7.15 (d, *J* = 8.5 Hz, 2H), 7.08 (d, *J* = 7.5 Hz, 2H), 7.04 (d, *J* = 4.0 Hz, 1H), 6.76 (t, *J* = 6.3 Hz, 1H), 6.35 (d, *J* = 4.0 Hz, 1H), 6.26 (s, 1H), 5.95 (s, 1H), 5.71 (s, 1H), 4.38-4.19 (m, 1H), 4.17-4.00 (m, 3H), 3.99-3.93 (m, 1H), 3.71 (br s, 2H), 3.46 (br s, 2H), 3.32 (br s, 1H), 3.22 (t, *J* = 7.7 Hz, 2H), 3.09-2.92 (m, 3H), 2.56 (t, *J* = 7.6 Hz, 2H), 2.51 (s, 3H), 2.29 (s, 3H), 2.08-2.06 (m, 1H), 2.03-1.98 (m, 2H), 1.95-1.89 (m, 2H), 1.72-1.61 (m, 2H), 1.52-1.39 (m, 2H); ^{13}C NMR (151 MHz, $(CD_3)_2CO$) δ 171.2, 168.7, 161.4, 161.4, 161.1, 160.4, 159.3, 159.1, 157.5, 154.2, 149.4, 145.0, 135.8, 134.4, 133.5, 130.9, 129.5, 127.1, 125.7, 124.8, 124.5, 121.0, 120.0, 117.7, 96.5, 59.8, 53.0, 47.2, 47.1, 46.4, 35.4, 34.9, 32.9, 31.9, 27.9, 25.1, 14.9, 11.2; HRMS (ESI) *m/z*: $[M+H]^+$ calcd. for $C_{45}H_{53}BF_2N_9O_3$: 816.4327, found: 816.4324.

LC-MS/MS Measurements

Protein Labelling and Digestion

Full-length recombinant BTK (MRC PPU Reagents, University of Dundee, UK, #DU12110) was diluted to 5 µM in Tris buffer (50 mM Tris-HCl, 10 mM $MgCl_2$, 100 mM KCl, 50 µM DTT,

0.05% Tween-20, pH 7.4) and treated with either DMSO or 50 µM of **Evo-2**. The samples (50 µL, 20 µg BTK), were incubated at room temperature for 1 h. **Evo-2** treatment resulted in a red precipitate, dissolved by adding 2% SDS to form a green solution. Samples were processed using a modified SP3 method[4] reduced in 10 mM DTT at 56°C for 30 min, alkylated in 20 mM iodoacetamide at room temperature for 30 min and bound to washed hydrophobic and hydrophilic Sera-Mag™ SpeedBeads (Carboxylate-Modified, Cytiva) at a 10:1 bead to protein ratio. Proteins were precipitated with 70% acetonitrile, washed with 80% ethanol, and dried. Beads were resuspended in 50 mM TEAB, and proteins were digested overnight with Trypsin/Lys-C Mix (1:25, Promega). Magnetic beads were removed, and peptides were cleaned with High Protein and Peptide Recovery Detergent Removal Spin Columns, and Pierce Peptide Desalting Spin Columns (Thermo Fisher Scientific).

Proteomics Analysis

Samples were analysed using an Orbitrap Eclipse Tribrid Mass Spectrometer coupled with an Easy-nLC 1200 liquid chromatography system (Thermo Fisher Scientific). Peptides were separated on a 35 cm x 75 µm in-house packed analytical column (particle size 3 µm, Reprosil-Pur C18, Dr. Maisch) using a 55-minute stepped gradient from 5% to 80% acetonitrile in 0.2% formic acid at 300 nL/min. Precursor ion mass spectra were acquired at 120,000 resolution. Abundant precursors or those from an inclusion list (*m/z* 743.14 and 990.51 Da, and 25 ppm mass tolerance) were isolated with an *m/z* window of 1.4 and fragmented by higher-energy collisional dissociation (HCD) at 30%. Fragment spectra were recorded at 30,000 resolution.

Database Search

Raw files were analysed with Proteome Discoverer (version 3.0, Thermo Fisher Scientific). The data was matched against BTK sequence (Q06187) retrieved from SwissProt database using Sequest HT (v1.17) with a precursor tolerance of 7 ppm and fragment ion tolerance of 0.02 Da. Tryptic peptides with 1 missed cleavage were accepted. Methionine oxidation (M), deamidation of asparagine and glutamine (N, Q), cysteine carbamidomethylation (C), and cysteine probe adduct (441.22734 Da, C) were set as variable modifications. Fragments with H_2O or NH_3 loss were included in the search. Fragment spectra were manually inspected to verify correct matching. A detailed list of all the identified peptides can be found in the supporting information file.

Biochemical Assays

In-Gel Fluorescence Labelling of Recombinant BTK

For covalent binding tests of **Evo-1** and **Evo-2** to BTK, 100 ng (1 µL) of full-length recombinant BTK (#V2941, Promega) was combined with 1 µL of **Evo-1** or **Evo-2** (4 µM to 62.5 nM) and 18 µL PBS, incubated for 1 h at room temperature. Samples were mixed with 4x LDS sample buffer (Thermo Fisher Scientific #NP0007), and reducing agent (NuPAGE sample reducing agent, Thermo Fisher Scientific, #NP0009), and the proteins were separated by electrophoresis on 4-12% NuPAGE Bis-Tris gels (Thermo Fisher Scientific, #NP0321). A ChemiDoc Imager (Bio-Rad, Alexa 488 filter) was used for fluorescence detection.

ADP-Glo Assay-Determination of IC_{50} Values

The ADP-Glo Kinase Assay (Promega) was performed using the supplied protocol with full-length recombinant BTK (MRC PPU Reagents, University of Dundee, UK, #DU12110). All procedures were at room temperature, with dilutions in Tris buffer (40 mM Tris-HCl, 20 mM $MgCl_2$, 2 mM $MnCl_2$, 0.1 mg/mL BSA, 50 µM DTT, pH 7.5). The kinase reaction quadruplicates, (5 µL reaction volume) were carried out in a 384-well White Polystyrene Microplate (Corning model 3824) with 10 ng of BTK, 50 µM ATP, 0.2 µg/µL Poly(Glu_4Tyr_1), and test compounds (0-1000 µM). Final DMSO concentration was 1%. After pre-incubation (1 µL compound, 2 µL BTK solution for 30 min), 2 µL substrate solution was added. The reaction was incubated for 60 min, followed by 5 µL ADP-Glo Reagent, and another 40 min incubation. Kinase Detection Reagent (10 µL) was added, and the solution was incubated for 60 min. Luminescence was measured on a SpectraMax iD5 Microplate Reader (Molecular Devices) using 1000 ms integration time. Luminescence values were converted to % activity, normalized to the positive control, and plotted using non-linear regression of the Sigmoidal dose-response curve. The IC_{50} values were determined and shown as mean ± SD.

ADP-Glo Assay-ATP Competition Experiment

To determine ATP-binding interference by **Evo-1** and **Evo-2**, BTK reactions were performed in triplicates (5 µL volume, 10 ng/µL BTK, 500 nM test compounds, 0-200 µM ATP, 0.2 µg/µL Poly(Glu_4Tyr_1, 1% DMSO). After 60 min incubation, the ADP-Glo procedure was performed as described above. Relative luminescence values (mean ± SD, after subtracting the negative control) were plotted versus the ATP concentrations for each compound.

Cellular Assays and Confocal Microscopy

Cell Lines

Ramos B-cells, and Jurkat T- cells (Clone E6-1) were purchased from the American Type Culture Collection (ATCC). Cells were maintained in Iscove's Modified Dulbecco's Medium with 25 mM HEPES (IMDM1x Glutamax, Gibco), supplemented with 10% heat-inactivated fetal bovine serum (FBS, Gibco), penicillin (100 U/mL), streptomycin (100 µg/mL) (HyClone Cytiva), and 50 µM β-mercaptoethanol (Gibco). Cells were grown at 37°C in a humidified 5%

CO2 atmosphere, and tested for mycoplasma contamination (Mycoplasmacheck test, Eurofins Genomics, Germany).

Flow Cytometry

Ramos and Jurkat cells (500,000 cells/mL) were treated in a 96-well microplate with 0.1 % DMSO, 4 µM compound **2**, **Evo-1**, or **Evo-2** (100 nM to 4 µM) at 37°C in a 5% CO_2 atmosphere for 2 h (*n* = 3). Cells were centrifuged (3 min, 300x*g*, 20°C), the supernatant was removed, and the cells were resuspended and washed twice with PBS. Cells for immediate acquisition were resuspended in 100 µL PBS; those for washout were resuspended in 200 µL fresh medium, incubated for 16 h (37°C, 5% CO_2), and then washed twice with PBS and resuspended in 100 µL PBS. Data was acquired using a FACSLyric flow cytometer (λ_{exc} = 488 nm, λ_{em} = 527 nm BD Biosciences), and analysed with FlowJo software (version 10, TreeStar Inc.).

In-Gel Fluorescence Detection of Cellular Proteins Labelled by the Probes

Ramos and Jurkat cells (500,000 cells/mL) were treated for 2 h with 0.1% DMSO or 500 nM **Evo-1** and **Evo-2**. For the competition experiment, cells were pretreated with or without 5 µM ibrutinib for 30 min followed by 2-h treatment with 0.1% DMSO or 500 nM **Evo-1**, and **Evo-2**. For the cellular labelling experiment with different concentrations of **Evo-2**, cells were treated with 0.1 % DMSO or **Evo-2**, (100 nM to 4 µM). Cells were harvested, washed thrice with PBS, and lysed on ice for 30 min with 1x RIPA buffer (Cell Signalling, #9806) containing protease and phosphatase inhibitors (Halt Protease and Phosphatase Inhibitor Cocktail, Thermo Fisher Scientific #87785). Lysates were sonicated (3x30s), centrifuged (28,000xg, 10 min), and the protein concentration was determined using the BCA protein assay (Pierce BCA Protein Assay Kit, ThermoScientific, #23225). Samples (16-19 µg total protein) were mixed with 4xLDS sample buffer and reducing agent, separated by electrophoresis on 4-12% NuPAGE Bis-Tris gels. Gels were scanned for BODIPY tag fluorescence (ChemiDoc Imager Bio-Rad, Alexa488 filter). After the fluorescence scanning, gels were stained for total proteins (Colloidal Blue Kit, Thermo Fisher Scientific, #LC6025).

Western Blot

Proteins were transferred to nitrocellulose membranes (Bio-Rad) after electrophoresis. Membranes were blocked with 5% BSA in TBS-Tween (for phospho-BTK and BTK detection, and 3% BSA in TBS-Tween for β-actin detection). After blocking, the membranes were incubated overnight at 4°C with primary antibodies: rabbit anti-phospho-BTK (Tyr233) (Cell Signaling, #87141, 1:1000 in 5% milk/ TBS-Tween), mouse anti-BTK antibody (Cell Signalling, #56044, 1:1000 in 5% BSA/ TBS-Tween), and mouse anti-β-actin (Abcam, #ab8226, 1:1000 in 3% BSA/ TBS-Tween). Membranes were washed twice with TBS-Tween, then incubated for

30 min at room temperature with secondary antibodies: goat anti-mouse Alexa633 (Invitrogen, #A21050) or HRP-conjugated antibodies (donkey anti-rabbit–HRP, Sigma #NA934V; sheep anti-mouse-HRP Sigma #NA931V, 1:5000 in 5% milk/ TBS-Tween). Fluorescence was detected using a ChemiDoc Imager (Alexa647 filter). Chemiluminescence signals were developed with Supersignal HRP substrate (Thermo Fisher Scientific, #23225) for phospho-BTK and BTK, and Immobilon forte HRP substrate (Millipore) for β-actin detection. Images were acquired with a digital camera (ChemiDoc, Bio-Rad). The membrane was stripped using Restore stripping buffer (Thermo Fisher Scientific, #21059) between detections.

Immunoprecipitation

Ramos cells (500,000 cells/mL) were treated in 10 cm^2 dishes with either 0.1% DMSO or 4 μM **Evo-2** for 4 h**.** Cells were harvested by centrifugation (5 min, 200xg , 4°C) washed thrice with ice-cold PBS, and lysed on ice for 30 min in 200 μL lysis buffer (50 mM Tris-HCl, 150 mM NaCl, 1% NP-40, pH 7.4) containing protease and phosphatase inhibitors. Lysates were sonicated thrice for 30s, centrifuged (14,000xg, 10 min, 4°C), and protein concentrations were determined using the BCA assay. Samples were adjusted to the same concentration, and lysates were immunoprecipitated overnight at 4˚C with rabbit anti-BTK antibodies (Abcam, #ab137503) or rabbit IgG isotype control, coupled to Dynabeads Protein G (Thermo Fisher Scientific #10004D). Immunoprecipitated samples were mixed with 4x LDS sample buffer and reducing agent, then separated by electrophoresis on 4-12% NuPAGE Bis-Tris gels. Gels were transferred to PVDF membranes, and fluorescence of **Evo-2** bound to BTK was detected using a ChemiDoc apparatus (Alexa488 filter). Membranes were then immunoblotted with mouse-anti-BTK and mouse anti-β-actin antibodies, followed by sheep anti-mouse HRP antibodies to detect BTK and β-actin, as described in the Western blot section.

Cell Viability Assay

Ramos cells (600,000 cells/mL) were treated with **Evo-1** and **Evo-2** at concentrations ranging from 0 to 10 μM for 2 and 4 h respectively. Control wells with 0.1 % DMSO or no treatment were included. Post-treatment, 10 μL of each cell sample was mixed with 10 μL 0.4% Trypan blue (Logos Biosystems, #T13001), and cell viability was measured using a Luna-II automated cell counter (Logos Biosystems). Measurements were performed in triplicate. Percent cell viability was calculated as: %viability = (A/A')*100, where A is mean cell viability for each concentration and A' mean cell viability of the DMSO control. Mean viability was averaged for each aliquot.

BTK Activity

Ramos cells (500,000 cells/mL) were pre-treated with or without 5 μM ibrutinib for 1 h at 37°C, and then treated with 500 nM **Evo-2** for 2 h. Cells were washed twice with PBS, resuspended in medium, and incubated with or without anti-human IgM (Jackson ImmunoResearch, #109-006-129, 10 μg/mL) for 10 min at 37°C to stimulate BCR signalling. Cells were harvested, and immunoblots of phospho-BTK, total-BTK, and β-actin were performed as described in the Western blot section.

Cellular Uptake of Evo-2

Ramos cells (500,000 cells/mL) were pelleted by centrifugation at 200xg for 5 min. Growth medium was replaced with Iscove's Modified Dulbecco's Medium without phenol red, supplemented with 10% FBS and 50 μM β-mercaptoethanol. Cells were treated with 500 nM **Evo-2**, and images of cellular uptake were captured every second immediately after addition without washing. Imaging data were collected on a Zeiss LSM880 Airyscan using a 63x/1.2 oil Corr FCS M27 objective with excitation at 488 nm and BP495-550+LP620 filters. Conditions were maintained at 37°C and 5% CO_2. Image processing used ZEN software (Zeiss), and analysis with ImageJ[5].

Fluorescence Bioimaging Experiments

Intracellular fluorescence and distribution of **Evo-2** within Ramos cells were analysed either using a Carl Zeiss LSM 880 Airyscan microscope with C-Apochromat 40x/1.2 W Corr FCS M27, or 63x/1.2 W Corr FCS M27, or 63x/1.2 oil Corr FCS M27 objectives. Cells were visualised upon excitation at 488 or 633 nm, using suitable filters (BP495-550+LP620 or BP420-480+LP605). Bright-field images were captured with a 405 nm laser. Conditions were maintained at 37°C and 5% CO_2. Image processing used ZEN software, and analysis was performed with ImageJ[5].

Immunostaining experiments

Ramos cells (500,000 cells/mL) were treated for 2 h with 0.1% DMSO or 1 μM **Evo-2** at 37°C, 5% CO_2. Cells were harvested, washed twice with PBS, resuspended in PBS ($1x10^6$ cells/mL), and transferred to 12-well plates with coverslips. Cells adhered by sedimentation for 30 min at room temperature, fixed with 4% paraformaldehyde in PBS for 10 min, washed twice with PBS and permeabilized with 0.1% Triton X with 6 μg/mL goat IgG in blocking buffer (5% fish gelatine in PBS) for 1 h at room temperature. Cells were incubated with primary antibodies (anti-mouse BTK, Cell Signalling, #56044, 1:50 in 0.1% Triton X with 6 μg/mL goat IgG in blocking buffer; anti-mouse IgG2b isotype control, Abcam, #ab170192, 1:1000 in same buffer) for 1 h at room temperature. Thereafter, cells were washed thrice with PBS and incubated with goat anti-mouse Alexa 633 secondary antibody (Thermo Fisher Scientific, #A21050) for 1 h at room temperature. Cells were washed thrice with PBS, once with water,

and mounted on slides (Superfrost ThermoScientific, #AGAA000080#32) with ProLong Gold (Invitrogen, #P36930). Imaging data were collected on a Zeiss LSM880 microscope using a 63x/1.2 W Corr FCS M27 objective. Image processing used ZEN software, and co-localisation analysis with ImageJ[5] using JACoP toolbox[6].

Degradation of Evo-2-labelled BTK

Ramos cells (420,000 cells/mL) were treated for 2 h with 0.1% DMSO or 1 µM Evo-2 at 37°C, 5% CO_2. Cells were harvested, washed twice with PBS, resuspended in medium and treated with 10 nM PROTAC **NX-5948** for 0, 30 and 120 min at 37°C, 5% CO_2. Cells were harvested, and immunoblots of total-BTK, and β-actin were performed as described in the Western blot section.

For the bio-imaging experiments with PROTAC **NX-5948**, Ramos cells were treated with **Evo-2** were attached to the bottom of imaging dishes (Ibidi, #81158) using a Cell-Tak adhesive mixture (Corning, #354240). It was prepared with a mixture of sodium bicarbonate (75 g/L), sodium hydroxide (40 g/L) and Cell-Tak (2.03 g/L) at a volume ratio of 291:5:4. For each well, 500 µL of Cell-Tak mix was added and incubated at room temperature for 30 min. The Cell-Tak mix was then removed, the dishes were washed twice with H_2O and completely dried off. Next, Ramos cells suspended in PBS and previously treated with either **Evo-2** or 0.1% DMSO, were added to the wells. Plated cells were incubated at 37°C, 5% CO_2 for 45 min and unadhered cells were carefully removed. Cells were then treated with 50 nM LysoTracker DeepRed (Invitrogen, #L12492) for 30 min at 37°C, 5% CO_2, and after washing once with PBS, media was changed to pre-warmed IMDM no phenol red (Glutamax, Gibco) supplemented with 10% heat-inactivated FBS. Then, 10 nM PROTAC **NX-5948** was added to the plated cells and cells were imaged at 0.5, 1 and 2 h of treatment with PROTAC. Cells were imaged using a Carl Zeiss LSM 980 confocal microscope equipped with a 63x/1.4 oil DIC M27 objective. Cells were visualised upon excitation at 488 nm and 639 nm by using a 32-channel GaAsP PMT Detector in the green spectral range (464-517 nm) and a PMT Multialkaline Detector centered in the red region (647-700 nm). Bright-field images were captured with a halogen lamp. Conditions were maintained at 37°C and 5% CO_2. Image processing used ZEN software, and analysis was performed with ImageJ[5].

Supplementary Figures and Tables

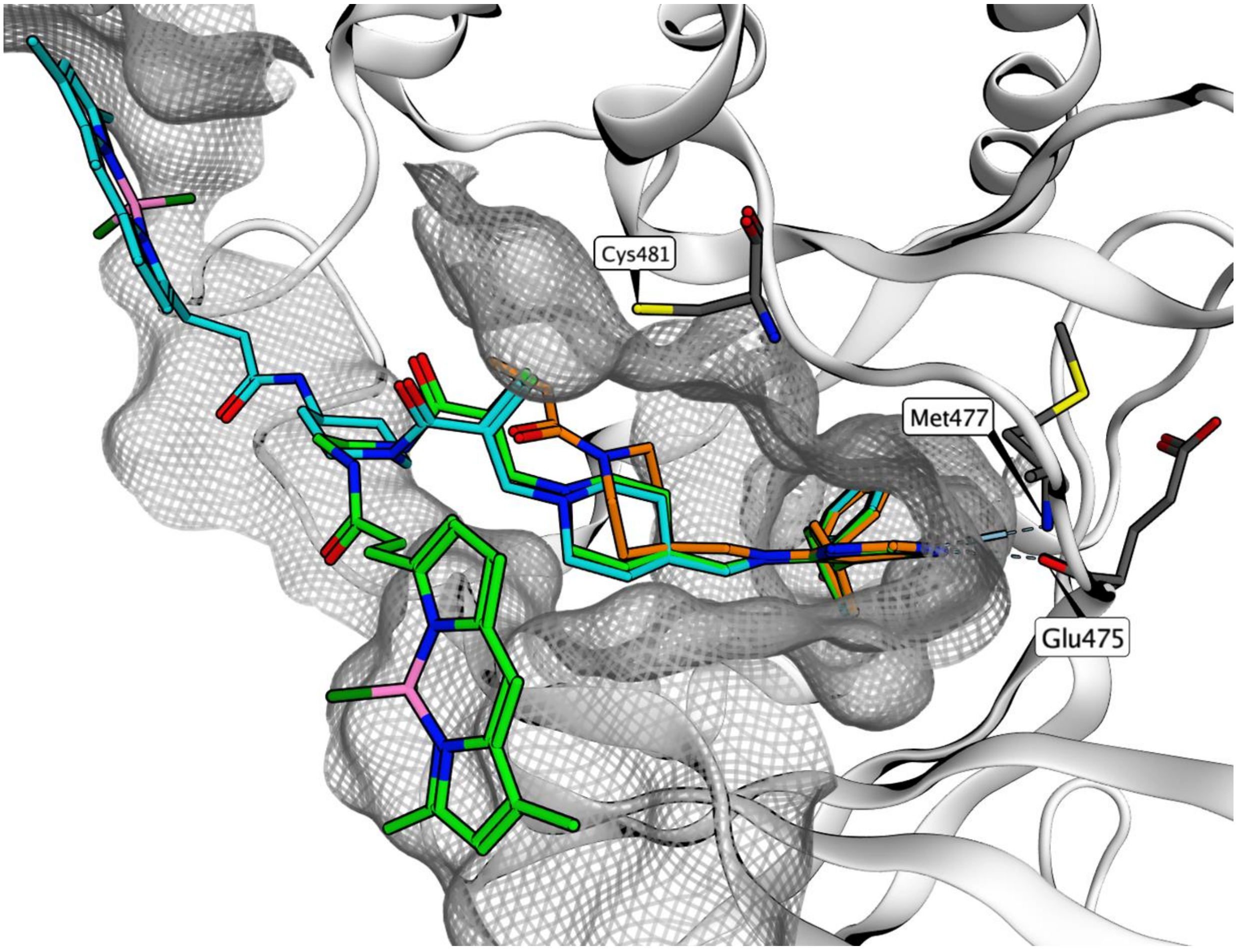

Figure S1. Molecular docking structures of **Evo-1** (light green carbons) and **Evo-2** (cyan carbons) bound to the kinase domain of BTK superimposed on the crystal structure of evobrutinib (orange carbons, PBD ID: 6OMU). A 4.5 Å molecular surface of all BTK residues near the ligands is shown. The interactions between the aminopyrimidine ring and the hinge region are illustrated with dashed lines.

Table S1 | Optimisation of the Suzuki-Miyaura reaction[a]

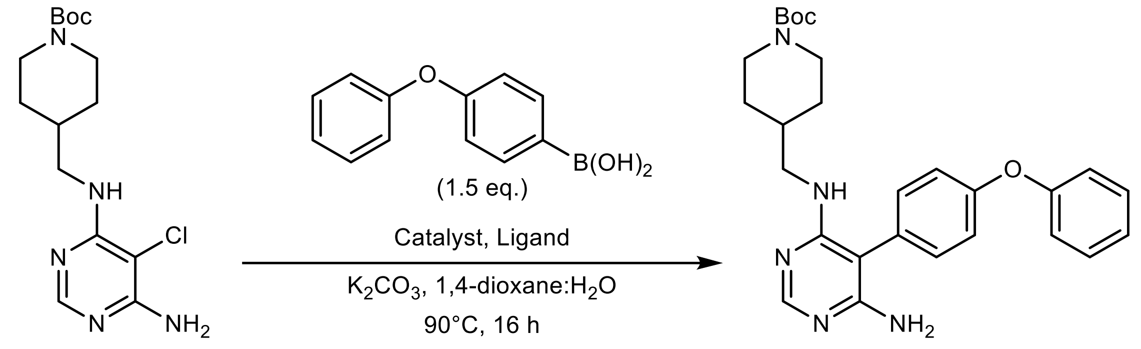

6 **7**

Entry	Catalyst	Ligand	K_2CO_3 (eq.)	1,4-dioxane:H_2O	Yield (%)[b]
1[c]	$Pd(OAc)_2$ (10 mol%)	SPhos (0.1 eq.)	3	9:1	28
2	$Pd(OAc)_2$ (10 mol%)	XPhos (0.1 eq.)	2	2:1	29
3	PEPPSI-IPr (5 mol%)	-	2	2:1	41
4	XPhos Pd G2 (5 mol%)	-	2	2:1	55
5	SPhos Pd G3 (5 mol%)	-	2	2:1	55
6[d]	SPhos Pd G3 (5 mol%)	-	2	4:1	67
7[e]	SPhos Pd G4 (2.5 mol%)	-	2	4:1	76

[a] Reactions were conducted at 0.4 mmol scale. [b] Isolated yields. [c] Reaction was run in the microwave reactor at 150°C for 2 h. [d] Reaction run for 2 h. [e] Reaction conditions were performed at 1.5 mmol scale.

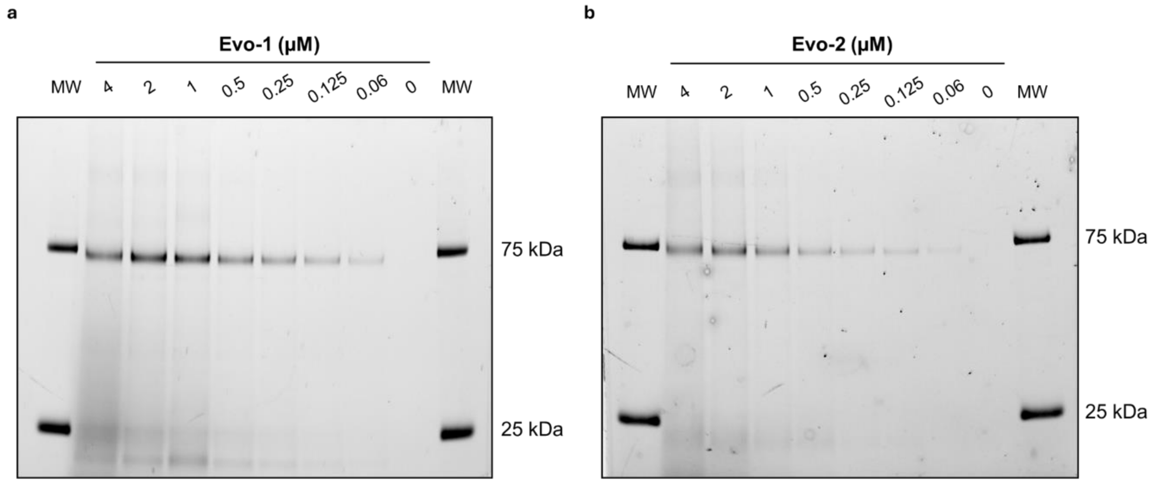

Figure S2. **a** In-gel fluorescence scanning of dose-dependent labelling of BTK by **Evo-1**. **b** In-gel fluorescence scanning of dose-dependent labelling of BTK by **Evo-2**. In both cases, recombinant BTK (0.1 μg) was incubated with decreasing concentrations of **Evo-1** and **Evo-2** for 1 h and then imaged with ChemiDoc apparatus (Alexa488 filter). The molecular weight (MW) of the fluorescent markers 75 and 25 kDa are shown on the far left and right of the gels.

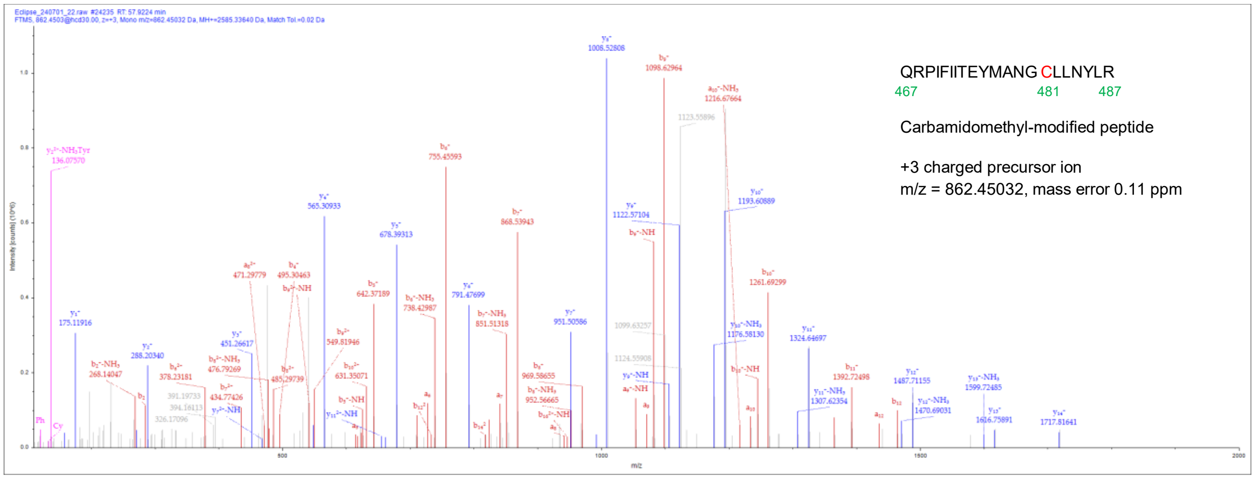

Figure S3. MS/MS-spectrum of the +3 charged precursor ion of the carbamidomethyl-modified peptide (467-487), resulting from BTK incubation with DMSO. Peaks are coloured as follows: y-ions in blue, a- and b-ions in red, immonium ions in magenta, and other ions in grey. The fragment ions that matched in the database search are highlighted in **Tables S2** and **S3**.

Table S2 | Fragments matched for carbamidomethyl-modified peptide (+3 charged precursor ion)[a]

#1	a^{+}	a^{2+}	b^{+}	b^{2+}	Immonium	Seq.uence	y^{+}	y^{2+}	#2
1	101.0709	51.0391	129.0659	65.0366	101.0709	Q			21
2	257.1721	129.0897	285.1670	143.0871	129.1135	R	2457.2781	1229.1427	20
3	354.2248	177.6161	382.2197	191.6135	70.0651	P	2301.1770	1151.0921	19
4	467.3089	234.1581	495.3038	248.1555	86.0964	I	2204.1242	1102.5658	18
5	614.3773	307.6923	642.3722	321.6897	120.0808	F	2091.0402	1046.0237	17
6	727.4614	364.2343	755.4563	378.2318	86.0964	I	1943.9718	972.4895	16
7	840.5454	420.7764	868.5403	434.7738	86.0964	I	1830.8877	915.9475	15
8	941.5931	471.3002	969.5880	485.2976	74.0600	T	1717.8036	859.4055	14
9	1070.6357	535.8215	1098.6306	549.8189	102.0550	E	1616.7560	808.8816	13
10	1233.6990	617.3532	1261.6939	631.3506	136.0757	Y	1487.7134	744.3603	12
11	1364.7395	682.8734	1392.7344	696.8709	104.0529	M	1324.6500	662.8287	11
12	1435.7766	718.3920	1463.7715	732.3894	44.0495	A	1193.6096	597.3084	10
13	1549.8196	775.4134	1577.8145	789.4109	87.0553	N	1122.5724	561.7899	9
14	1606.8410	803.9241	1634.8359	817.9216	30.0338	G	1008.5295	504.7684	8
15	1766.8717	883.9395	1794.8666	897.9369	133.0430	C-Alkylated	951.5081	476.2577	7
16	1879.9557	940.4815	1907.9506	954.4790	86.0964	L	791.4774	396.2423	6
17	1993.0398	997.0235	2021.0347	1011.0210	86.0964	L	678.3933	339.7003	5
18	2107.0827	1054.0450	2135.0776	1068.0425	87.0553	N	565.3093	283.1583	4
19	2270.1460	1135.5767	2298.1410	1149.5741	136.0757	Y	451.2663	226.1368	3
20	2383.2301	1192.1187	2411.2250	1206.1162	86.0964	L	288.2030	144.6052	2
21					129.1135	R	175.1190	88.0631	1

[a]The table shows calculated masses for a-, b-, y-, and immonium ions. The fragment ions detected in the MS/MS spectra that matched in the database search are highlighted in colour.

Table S3 | Fragments matched for carbamidomethyl-modified peptide (+3 charged precursor ion)[a]

#1	a-NH_3^+	a-NH_3^{2+}	b-NH_3^+	b-NH_3^{2+}	Seq.uence	y-NH_3^+	y-NH_3^{2+}	#2
1	84.0444	42.5258	112.0393	56.5233	Q			21
2	240.1455	120.5764	268.1404	134.5739	R	2440.2516	1220.6294	20
3	337.1983	169.1028	365.1932	183.1002	P	2284.1505	1142.5789	19
4	450.2823	225.6448	478.2772	239.6423	I	2187.0977	1094.0525	18
5	597.3507	299.1790	625.3457	313.1765	F	2074.0136	1037.5105	17
6	710.4348	355.7210	738.4297	369.7185	I	1926.9452	963.9763	16
7	823.5189	412.2631	851.5138	426.2605	I	1813.8612	907.4342	15
8	924.5666	462.7869	952.5615	476.7844	T	1700.7771	850.8922	14
9	1053.6091	527.3082	1081.6041	541.3057	E	1599.7294	800.3683	13
10	1216.6725	608.8399	1244.6674	622.8373	Y	1470.6868	735.8471	12
11	1347.7130	674.3601	1375.7079	688.3576	M	1307.6235	654.3154	11
12	1418.7501	709.8787	1446.7450	723.8761	A	1176.5830	588.7951	10
13	1532.7930	766.9001	1560.7879	780.8976	N	1105.5459	553.2766	9
14	1589.8145	795.4109	1617.8094	809.4083	G	991.5030	496.2551	8
15	1749.8451	875.4262	1777.8400	889.4237	C-Alkylated	934.4815	467.7444	7
16	1862.9292	931.9682	1890.9241	945.9657	L	774.4509	387.7291	6
17	1976.0132	988.5103	2004.0082	1002.5077	L	661.3668	331.1870	5
18	2090.0562	1045.5317	2118.0511	1059.5292	N	548.2827	274.6450	4
19	2253.1195	1127.0634	2281.1144	1141.0608	Y	434.2398	217.6235	3
20	2366.2036	1183.6054	2394.1985	1197.6029	L	271.1765	136.0919	2
21					R	158.0924	79.5498	1

[a]The table shows calculated masses for a-, b-, and y-ions with loss of NH_3. The fragment ions detected in the MS/MS spectra that matched in the database search are highlighted in colour.

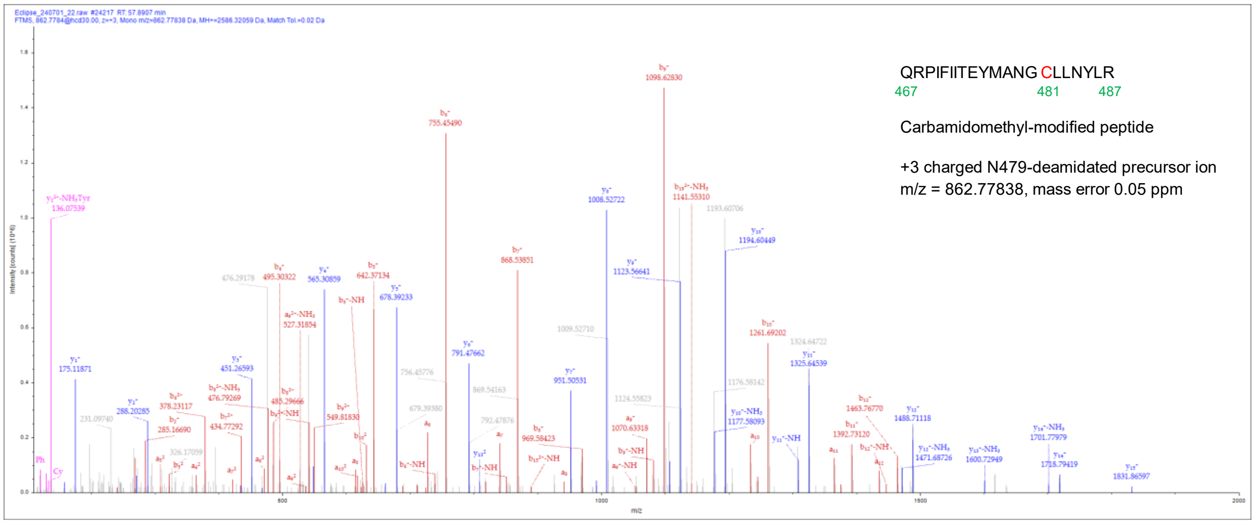

Figure S4. MS/MS-spectrum of the +3 charged N479-deamidated precursor ion of the carbamidomethyl-modified peptide (467-487), resulting from BTK incubation with DMSO. Peaks are coloured as follows: y-ions in blue, a- and b-ions in red, immonium ions in magenta, and other ions in grey. The fragment ions that matched in the database search are highlighted in **Tables S4** and **S5**.

Table S4 | Fragments matched for carbamidomethyl-modified peptide (+3 charged N-deamidated precursor ion)[a]

#1	a^+	a^{2+}	b^+	b^{2+}	Immonium	Seq.uence	y^+	y^{2+}	#2
1	101.0709	51.0391	129.0659	65.0366	101.0709	Q			21
2	257.1721	129.0897	285.1670	143.0871	129.1135	R	2458.2621	1229.6347	20
3	354.2248	177.6161	382.2197	191.6135	70.0651	P	2302.1610	1151.5842	19
4	467.3089	234.1581	495.3038	248.1555	86.0964	I	2205.1083	1103.0578	18
5	614.3773	307.6923	642.3722	321.6897	120.0808	F	2092.0242	1046.5157	17
6	727.4614	364.2343	755.4563	378.2318	86.0964	I	1944.9558	972.9815	16
7	840.5454	420.7764	868.5403	434.7738	86.0964	I	1831.8717	916.4395	15
8	941.5931	471.3002	969.5880	485.2976	74.0600	T	1718.7877	859.8975	14
9	1070.6357	535.8215	1098.6306	549.8189	102.0550	E	1617.7400	809.3736	13
10	1233.6990	617.3532	1261.6939	631.3506	136.0757	Y	1488.6974	744.8523	12
11	1364.7395	682.8734	1392.7344	696.8709	104.0529	M	1325.6341	663.3207	11
12	1435.7766	718.3920	1463.7715	732.3894	44.0495	A	1194.5936	597.8004	10
13	1550.8036	775.9054	1578.7985	789.9029	88.0393	N-Deamidated	1123.5565	562.2819	9
14	1607.8250	804.4162	1635.8199	818.4136	30.0338	G	1008.5295	504.7684	8
15	1767.8557	884.4315	1795.8506	898.4289	133.0430	C-Alkylated	951.5081	476.2577	7
16	1880.9397	940.9735	1908.9347	954.9710	86.0964	L	791.4774	396.2423	6
17	1994.0238	997.5155	2022.0187	1011.5130	86.0964	L	678.3933	339.7003	5
18	2108.0667	1054.5370	2136.0616	1068.5345	87.0553	N	565.3093	283.1583	4
19	2271.1301	1136.0687	2299.1250	1150.0661	136.0757	Y	451.2663	226.1368	3
20	2384.2141	1192.6107	2412.2090	1206.6082	86.0964	L	288.2030	144.6052	2
21					129.1135	R	175.1190	88.0631	1

[a]The table shows calculated masses for a-, b-, y-, and immonium ions. The fragment ions detected in the MS/MS spectra that matched in the database search are highlighted in colour.

Table S5 | Fragments matched for carbamidomethyl-modified peptide (+3 charged N-deamidated precursor ion)[a]

#1	a-NH_3^+	a-NH_3^{2+}	b-NH_3^+	b-NH_3^{2+}	Seq.uence	y-NH_3^+	y-NH_3^{2+}	#2
1	84.0444	42.5258	112.0393	56.5233	Q			21
2	240.1455	120.5764	268.1404	134.5739	R	2441.2356	1221.1214	20
3	337.1983	169.1028	365.1932	183.1002	P	2285.1345	1143.0709	19
4	450.2823	225.6448	478.2772	239.6423	I	2188.0817	1094.5445	18
5	597.3507	299.1790	625.3457	313.1765	F	2074.9977	1038.0025	17
6	710.4348	355.7210	738.4297	369.7185	I	1927.9292	964.4683	16
7	823.5189	412.2631	851.5138	426.2605	I	1814.8452	907.9262	15
8	924.5666	462.7869	952.5615	476.7844	T	1701.7611	851.3842	14
9	1053.6091	527.3082	1081.6041	541.3057	E	1600.7134	800.8604	13
10	1216.6725	608.8399	1244.6674	622.8373	Y	1471.6708	736.3391	12
11	1347.7130	674.3601	1375.7079	688.3576	M	1308.6075	654.8074	11
12	1418.7501	709.8787	1446.7450	723.8761	A	1177.5670	589.2872	10
13	1533.7770	767.3921	1561.7719	781.3896	N-Deamidated	1106.5299	553.7686	9
14	1590.7985	795.9029	1618.7934	809.9003	G	991.5030	496.2551	8
15	1750.8291	875.9182	1778.8240	889.9157	C-Alkylated	934.4815	467.7444	7
16	1863.9132	932.4602	1891.9081	946.4577	L	774.4509	387.7291	6
17	1976.9973	989.0023	2004.9922	1002.9997	L	661.3668	331.1870	5
18	2091.0402	1046.0237	2119.0351	1060.0212	N	548.2827	274.6450	4
19	2254.1035	1127.5554	2282.0984	1141.5529	Y	434.2398	217.6235	3
20	2367.1876	1184.0974	2395.1825	1198.0949	L	271.1765	136.0919	2
21					R	158.0924	79.5498	1

[a]The table shows calculated masses for a-, b-, and y-ions with loss of NH_3. The fragment ions detected in the MS/MS spectra that matched in the database search are highlighted in colour.

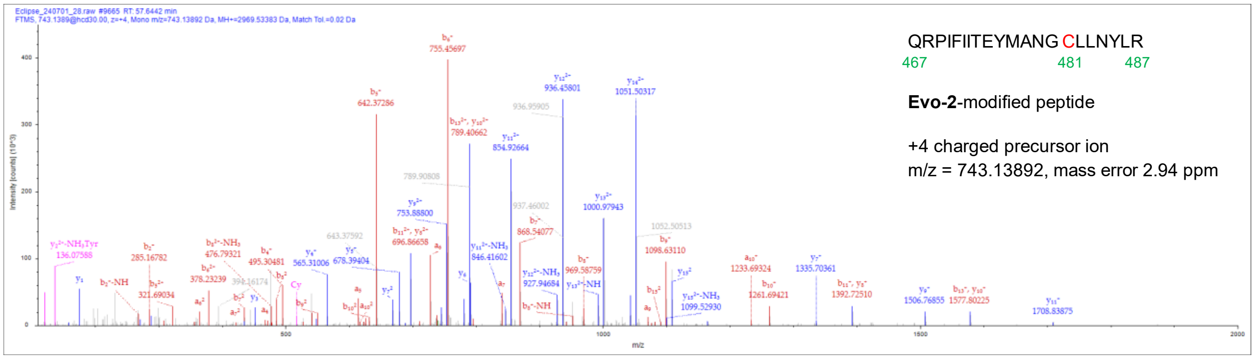

Figure S5. MS/MS-spectrum of the +4 charged precursor ion of the **Evo-2**-modified peptide (467-487), resulting from BTK incubation with **Evo-2**. Peaks are coloured as follows: y-ions in blue, a- and b-ions in red, immonium ions in magenta, and other ions in grey. The fragment ions that matched in the database search are highlighted in **Tables S6** and **S7**.

Table S6 | Fragments matched for Evo-2-modified peptide (+4 charged precursor ion)[a]

#1	a^{+}	a^{2+}	b^{+}	b^{2+}	Immonium	Seq.uence	y^{+}	y^{2+}	#2
1	101.0709	51.0391	129.0659	65.0366	101.0709	Q			21
2	257.1721	129.0897	285.1670	143.0871	129.1135	R	2841.4840	1421.2456	20
3	354.2248	177.6161	382.2197	191.6135	70.0651	P	2685.3829	1343.1951	19
4	467.3089	234.1581	495.3038	248.1555	86.0964	I	2588.3301	1294.6687	18
5	614.3773	307.6923	642.3722	321.6897	120.0808	F	2475.2461	1238.1267	17
6	727.4614	364.2343	755.4563	378.2318	86.0964	I	2328.1776	1164.5925	16
7	840.5454	420.7764	868.5403	434.7738	86.0964	I	2215.0936	1108.0504	15
8	941.5931	471.3002	969.5880	485.2976	74.0600	T	2102.0095	1051.5084	14
9	1070.6357	535.8215	1098.6306	549.8189	102.0550	E	2000.9618	1000.9846	13
10	1233.6990	617.3532	1261.6939	631.3506	136.0757	Y	1871.9192	936.4633	12
11	1364.7395	682.8734	1392.7344	696.8709	104.0529	M	1708.8559	854.9316	11
12	1435.7766	718.3920	1463.7715	732.3894	44.0495	A	1577.8154	789.4114	10
13	1549.8196	775.4134	1577.8145	789.4109	87.0553	N	1506.7783	753.8928	9
14	1606.8410	803.9241	1634.8359	817.9216	30.0338	G	1392.7354	696.8713	8
15	2151.0775	1076.0424	2179.0725	1090.0399	517.2489	C-Labelled	1335.7139	668.3606	7
16	2264.1616	1132.5844	2292.1565	1146.5819	86.0964	L	791.4774	396.2423	6
17	2377.2457	1189.1265	2405.2406	1203.1239	86.0964	L	678.3933	339.7003	5
18	2491.2886	1246.1479	2519.2835	1260.1454	87.0553	N	565.3093	283.1583	4
19	2654.3519	1327.6796	2682.3468	1341.6771	136.0757	Y	451.2663	226.1368	3
20	2767.4360	1384.2216	2795.4309	1398.2191	86.0964	L	288.2030	144.6052	2
21					129.1135	R	175.1190	88.0631	1

[a]The table shows calculated masses for a-, b-, y-, and immonium ions. The fragment ions detected in the MS/MS spectra that matched in the database search are highlighted in colour.

Table S7 | Fragments matched for Evo-2-modified peptide (+4 charged precursor ion)[a]

#1	a-NH_3^+	a-NH_3^{2+}	b-NH_3^+	b-NH_3^{2+}	Seq.uence	y-NH_3^+	y-NH_3^{2+}	#2
1	84.0444	42.5258	112.0393	56.5233	Q			21
2	240.1455	120.5764	268.1404	134.5739	R	2824.4574	1412.7324	20
3	337.1983	169.1028	365.1932	183.1002	P	2668.3563	1334.6818	19
4	450.2823	225.6448	478.2772	239.6423	I	2571.3036	1286.1554	18
5	597.3507	299.1790	625.3457	313.1765	F	2458.2195	1229.6134	17
6	710.4348	355.7210	738.4297	369.7185	I	2311.1511	1156.0792	16
7	823.5189	412.2631	851.5138	426.2605	I	2198.0670	1099.5372	15
8	924.5666	462.7869	952.5615	476.7844	T	2084.9830	1042.9951	14
9	1053.6091	527.3082	1081.6041	541.3057	E	1983.9353	992.4713	13
10	1216.6725	608.8399	1244.6674	622.8373	Y	1854.8927	927.9500	12
11	1347.7130	674.3601	1375.7079	688.3576	M	1691.8294	846.4183	11
12	1418.7501	709.8787	1446.7450	723.8761	A	1560.7889	780.8981	10
13	1532.7930	766.9001	1560.7879	780.8976	N	1489.7518	745.3795	9
14	1589.8145	795.4109	1617.8094	809.4083	G	1375.7088	688.3581	8
15	2134.0510	1067.5291	2162.0459	1081.5266	C-Labelled	1318.6874	659.8473	7
16	2247.1351	1124.0712	2275.1300	1138.0686	L	774.4509	387.7291	6
17	2360.2191	1180.6132	2388.2140	1194.6107	L	661.3668	331.1870	5
18	2474.2620	1237.6347	2502.2570	1251.6321	N	548.2827	274.6450	4
19	2637.3254	1319.1663	2665.3203	1333.1638	Y	434.2398	217.6235	3
20	2750.4094	1375.7084	2778.4044	1389.7058	L	271.1765	136.0919	2
21					R	158.0924	79.5498	1

[a]The table shows calculated masses for a-, b-, and y-ions with loss of NH_3. The fragment ions detected in the MS/MS spectra that matched in the database search are highlighted in colour.

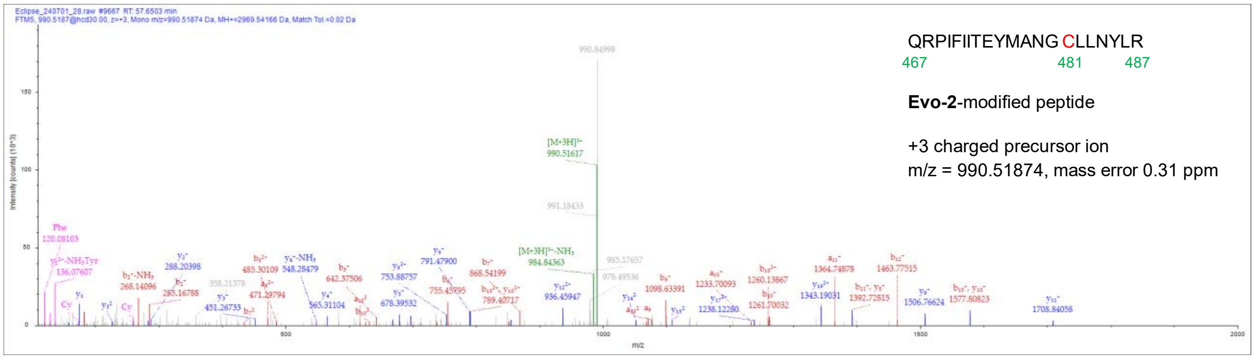

Figure S6. MS/MS-spectrum of the +3 charged precursor ion of the **Evo-2**-modified peptide (467-487), resulting from BTK incubation with **Evo-2**. Peaks are coloured as follows: y-ions in blue, a- and b-ions in red, immonium ions in magenta, precursor ions in green, and other ions in grey. The fragment ions that matched in the database search are highlighted in **Tables S8** and **S9**.

Table S8 | Fragments matched for Evo-2-modified peptide (+3 charged precursor ion)[a]

#1	a^{+}	a^{2+}	b^{+}	b^{2+}	Immonium	Seq.uence	y^{+}	y^{2+}	#2
1	101.0709	51.0391	129.0659	65.0366	101.0709	Q			21
2	257.1721	129.0897	285.1670	143.0871	129.1135	R	2841.4840	1421.2456	20
3	354.2248	177.6161	382.2197	191.6135	70.0651	P	2685.3829	1343.1951	19
4	467.3089	234.1581	495.3038	248.1555	86.0964	I	2588.3301	1294.6687	18
5	614.3773	307.6923	642.3722	321.6897	120.0808	F	2475.2461	1238.1267	17
6	727.4614	364.2343	755.4563	378.2318	86.0964	I	2328.1776	1164.5925	16
7	840.5454	420.7764	868.5403	434.7738	86.0964	I	2215.0936	1108.0504	15
8	941.5931	471.3002	969.5880	485.2976	74.0600	T	2102.0095	1051.5084	14
9	1070.6357	535.8215	1098.6306	549.8189	102.0550	E	2000.9618	1000.9846	13
10	1233.6990	617.3532	1261.6939	631.3506	136.0757	Y	1871.9192	936.4633	12
11	1364.7395	682.8734	1392.7344	696.8709	104.0529	M	1708.8559	854.9316	11
12	1435.7766	718.3920	1463.7715	732.3894	44.0495	A	1577.8154	789.4114	10
13	1549.8196	775.4134	1577.8145	789.4109	87.0553	N	1506.7783	753.8928	9
14	1606.8410	803.9241	1634.8359	817.9216	30.0338	G	1392.7354	696.8713	8
15	2151.0775	1076.0424	2179.0725	1090.0399	517.2489	C-Labelled	1335.7139	668.3606	7
16	2264.1616	1132.5844	2292.1565	1146.5819	86.0964	L	791.4774	396.2423	6
17	2377.2457	1189.1265	2405.2406	1203.1239	86.0964	L	678.3933	339.7003	5
18	2491.2886	1246.1479	2519.2835	1260.1454	87.0553	N	565.3093	283.1583	4
19	2654.3519	1327.6796	2682.3468	1341.6771	136.0757	Y	451.2663	226.1368	3
20	2767.4360	1384.2216	2795.4309	1398.2191	86.0964	L	288.2030	144.6052	2
21					129.1135	R	175.1190	88.0631	1

[a]The table shows calculated masses for a-, b-, y-, and immonium ions. The fragment ions detected in the MS/MS spectra that matched in the database search are highlighted in colour.

Table S9 | Fragments matched for Evo-2-modified peptide (+3 charged precursor ion)[a]

#1	a-NH_3^+	a-NH_3^{2+}	b-NH_3^+	b-NH_3^{2+}	Seq.uence	y-NH_3^+	y-NH_3^{2+}	#2
1	84.0444	42.5258	112.0393	56.5233	Q			21
2	240.1455	120.5764	268.1404	134.5739	R	2824.4574	1412.7324	20
3	337.1983	169.1028	365.1932	183.1002	P	2668.3563	1334.6818	19
4	450.2823	225.6448	478.2772	239.6423	I	2571.3036	1286.1554	18
5	597.3507	299.1790	625.3457	313.1765	F	2458.2195	1229.6134	17
6	710.4348	355.7210	738.4297	369.7185	I	2311.1511	1156.0792	16
7	823.5189	412.2631	851.5138	426.2605	I	2198.0670	1099.5372	15
8	924.5666	462.7869	952.5615	476.7844	T	2084.9830	1042.9951	14
9	1053.6091	527.3082	1081.6041	541.3057	E	1983.9353	992.4713	13
10	1216.6725	608.8399	1244.6674	622.8373	Y	1854.8927	927.9500	12
11	1347.7130	674.3601	1375.7079	688.3576	M	1691.8294	846.4183	11
12	1418.7501	709.8787	1446.7450	723.8761	A	1560.7889	780.8981	10
13	1532.7930	766.9001	1560.7879	780.8976	N	1489.7518	745.3795	9
14	1589.8145	795.4109	1617.8094	809.4083	G	1375.7088	688.3581	8
15	2134.0510	1067.5291	2162.0459	1081.5266	C-Labelled	1318.6874	659.8473	7
16	2247.1351	1124.0712	2275.1300	1138.0686	L	774.4509	387.7291	6
17	2360.2191	1180.6132	2388.2140	1194.6107	L	661.3668	331.1870	5
18	2474.2620	1237.6347	2502.2570	1251.6321	N	548.2827	274.6450	4
19	2637.3254	1319.1663	2665.3203	1333.1638	Y	434.2398	217.6235	3
20	2750.4094	1375.7084	2778.4044	1389.7058	L	271.1765	136.0919	2
21					R	158.0924	79.5498	1

[a]The table shows calculated masses for a-, b-, and y-ions with loss of NH_3. The fragment ions detected in the MS/MS spectra that matched in the database search are highlighted in colour.

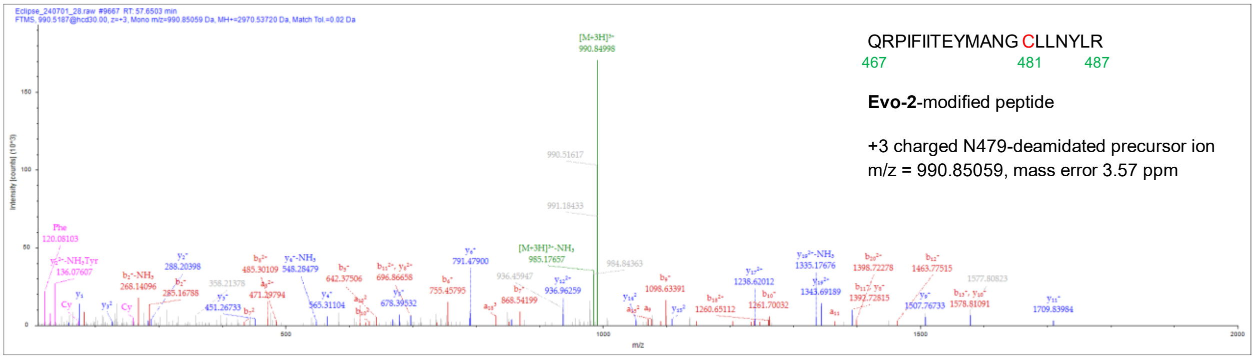

Figure S7. MS/MS-spectrum of the +3 charged N479-deamidated precursor ion of the **Evo-2**-modified peptide (467-487), resulting from BTK incubation with **Evo-2**. Peaks are coloured as follows: y-ions in blue, a- and b-ions in red, immonium ions in magenta, precursor ions in green, and other ions in grey. The fragment ions that matched in the database search are highlighted in **Tables S10** and **S11**.

Table S10 | Fragments matched for Evo-2-modified peptide (+3 charged N-deamidated precursor ion)[a]

#1	a^+	a^{2+}	b^+	b^{2+}	Immonium	Seq.uence	y^+	y^{2+}	#2
1	101.0709	51.0391	129.0659	65.0366	101.0709	Q			21
2	257.1721	129.0897	285.1670	143.0871	129.1135	R	2842.4680	1421.7376	20
3	354.2248	177.6161	382.2197	191.6135	70.0651	P	2686.3669	1343.6871	19
4	467.3089	234.1581	495.3038	248.1555	86.0964	I	2589.3141	1295.1607	18
5	614.3773	307.6923	642.3722	321.6897	120.0808	F	2476.2301	1238.6187	17
6	727.4614	364.2343	755.4563	378.2318	86.0964	I	2329.1617	1165.0845	16
7	840.5454	420.7764	868.5403	434.7738	86.0964	I	2216.0776	1108.5424	15
8	941.5931	471.3002	969.5880	485.2976	74.0600	T	2102.9935	1052.0004	14
9	1070.6357	535.8215	1098.6306	549.8189	102.0550	E	2001.9459	1001.4766	13
10	1233.6990	617.3532	1261.6939	631.3506	136.0757	Y	1872.9033	936.9553	12
11	1364.7395	682.8734	1392.7344	696.8709	104.0529	M	1709.8399	855.4236	11
12	1435.7766	718.3920	1463.7715	732.3894	44.0495	A	1578.7995	789.9034	10
13	1550.8036	775.9054	1578.7985	789.9029	88.0393	N-Deamidated	1507.7623	754.3848	9
14	1607.8250	804.4162	1635.8199	818.4136	30.0338	G	1392.7354	696.8713	8
15	2152.0616	1076.5344	2180.0565	1090.5319	517.2489	C-Labelled	1335.7139	668.3606	7
16	2265.1456	1133.0765	2293.1405	1147.0739	86.0964	L	791.4774	396.2423	6
17	2378.2297	1189.6185	2406.2246	1203.6159	86.0964	L	678.3933	339.7003	5
18	2492.2726	1246.6399	2520.2675	1260.6374	87.0553	N	565.3093	283.1583	4
19	2655.3359	1328.1716	2683.3309	1342.1691	136.0757	Y	451.2663	226.1368	3
20	2768.4200	1384.7136	2796.4149	1398.7111	86.0964	L	288.2030	144.6052	2
21					129.1135	R	175.1190	88.0631	1

[a]The table shows calculated masses for a-, b-, y-, and immonium ions. The fragment ions detected in the MS/MS spectra that matched in the database search are highlighted in colour.

Table S11 | Fragments matched for Evo-2-modified peptide (+3 charged N-deamidated precursor ion)[a]

#1	a-NH_3^+	a-NH_3^{2+}	b-NH_3^+	b-NH_3^{2+}	Seq.uence	y-NH_3^+	y-NH_3^{2+}	#2
1	84.0444	42.5258	112.0393	56.5233	Q			21
2	240.1455	120.5764	268.1404	134.5739	R	2825.4415	1413.2244	20
3	337.1983	169.1028	365.1932	183.1002	P	2669.3404	1335.1738	19
4	450.2823	225.6448	478.2772	239.6423	I	2572.2876	1286.6474	18
5	597.3507	299.1790	625.3457	313.1765	F	2459.2035	1230.1054	17
6	710.4348	355.7210	738.4297	369.7185	I	2312.1351	1156.5712	16
7	823.5189	412.2631	851.5138	426.2605	I	2199.0510	1100.0292	15
8	924.5666	462.7869	952.5615	476.7844	T	2085.9670	1043.4871	14
9	1053.6091	527.3082	1081.6041	541.3057	E	1984.9193	992.9633	13
10	1216.6725	608.8399	1244.6674	622.8373	Y	1855.8767	928.4420	12
11	1347.7130	674.3601	1375.7079	688.3576	M	1692.8134	846.9103	11
12	1418.7501	709.8787	1446.7450	723.8761	A	1561.7729	781.3901	10
13	1533.7770	767.3921	1561.7719	781.3896	N-Deamidated	1490.7358	745.8715	9
14	1590.7985	795.9029	1618.7934	809.9003	G	1375.7088	688.3581	8
15	2135.0350	1068.0211	2163.0299	1082.0186	C-Labelled	1318.6874	659.8473	7
16	2248.1191	1124.5632	2276.1140	1138.5606	L	774.4509	387.7291	6
17	2361.2031	1181.1052	2389.1980	1195.1027	L	661.3668	331.1870	5
18	2475.2461	1238.1267	2503.2410	1252.1241	N	548.2827	274.6450	4
19	2638.3094	1319.6583	2666.3043	1333.6558	Y	434.2398	217.6235	3
20	2751.3935	1376.2004	2779.3884	1390.1978	L	271.1765	136.0919	2
21					R	158.0924	79.5498	1

[a]The table shows calculated masses for a-, b-, and y-ions with loss of NH_3. The fragment ions detected in the MS/MS spectra that matched in the database search are highlighted in colour.

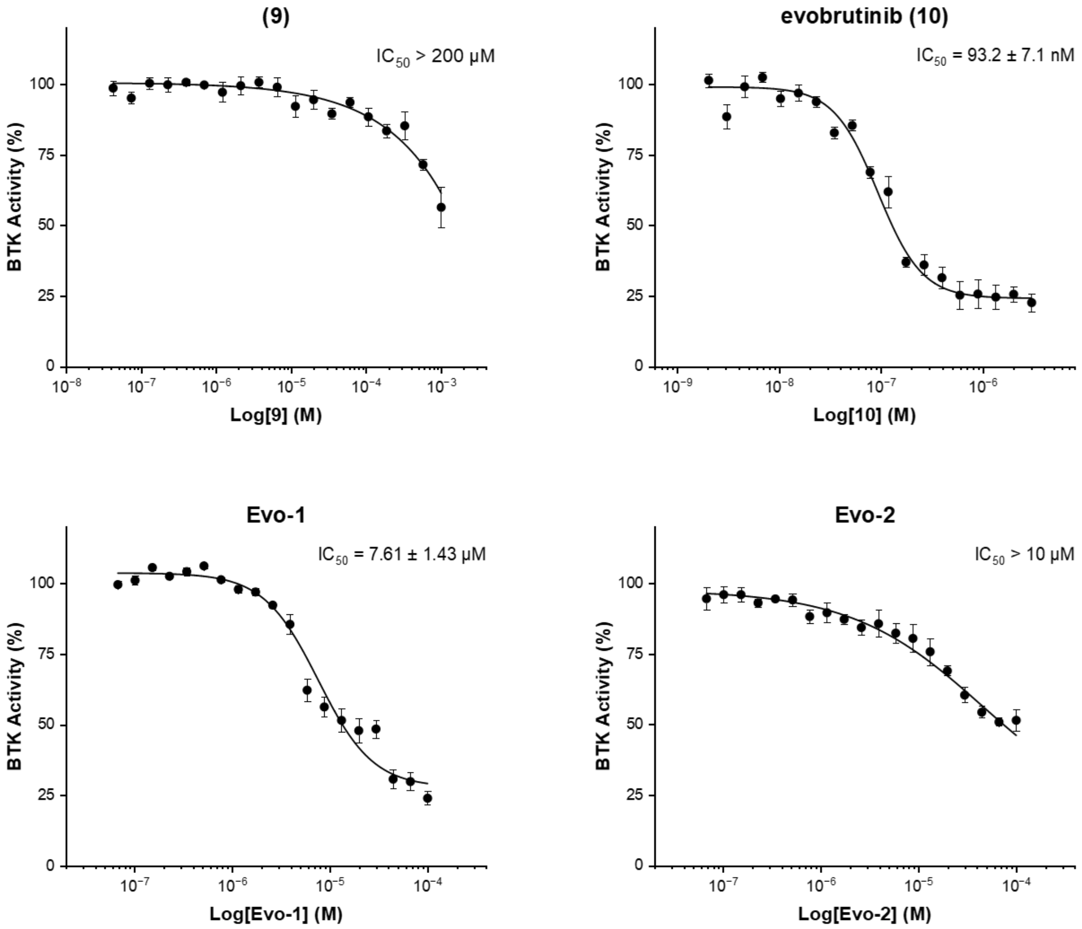

Figure S8. IC_{50} values of compounds 9, 10, Evo-1, and Evo-2 against full-length BTK. An ADP-Glo Assay was performed with 10 ng of BTK, 50 µM ATP, 0.2 µg/µL Poly(Glu_4Tyr_1), test compounds (0-1000 µM) and 1% DMSO. Data are presented as the mean ± SD (*n* = 4).

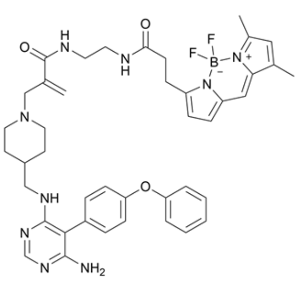

Evo-1

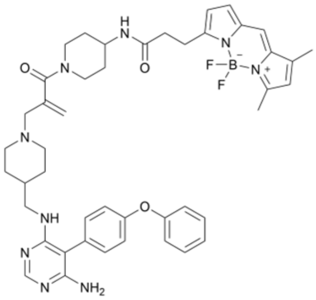

Evo-2

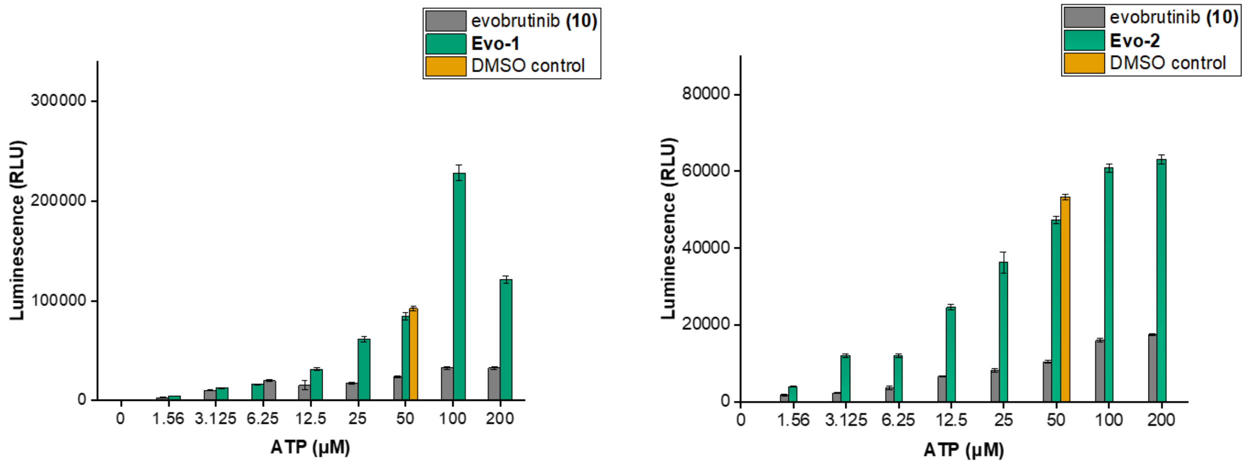

Figure S9. Evo-1 and Evo-2 do not interfere with the ATP-binding in recombinant BTK. An ADP-Glo Assay was performed with 10 ng of BTK, 500 nM **Evo-1** or **Evo-2** (green bars), or evobrutinib (grey bars) under increasing concentrations of ATP. Control experiments with DMSO under normal assay conditions (50 µM ATP, orange) were also performed. Luminescence is presented as relative luminescence units [RLU]. Data are presented as the mean ± SD (*n* = 3).

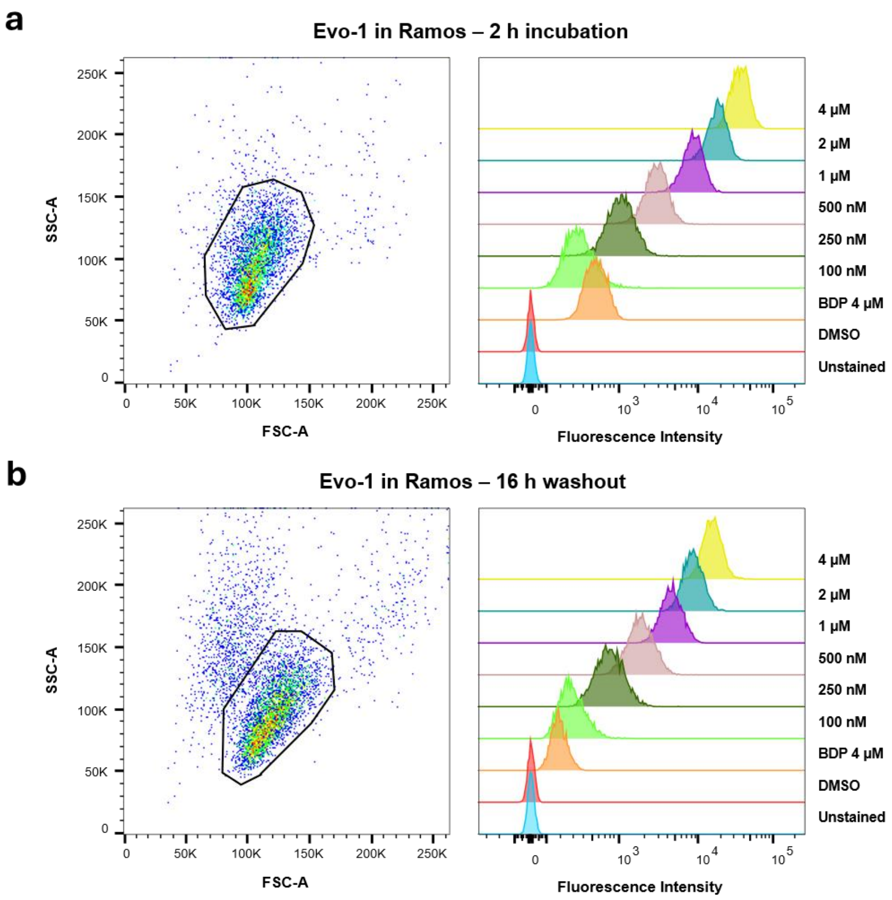

Figure S10. Cellular uptake of Evo-1 in Ramos cells. Histogram of fluorescence intensities after, **a** 2 h incubation and **b** 2 h incubation followed by 16 h washout.

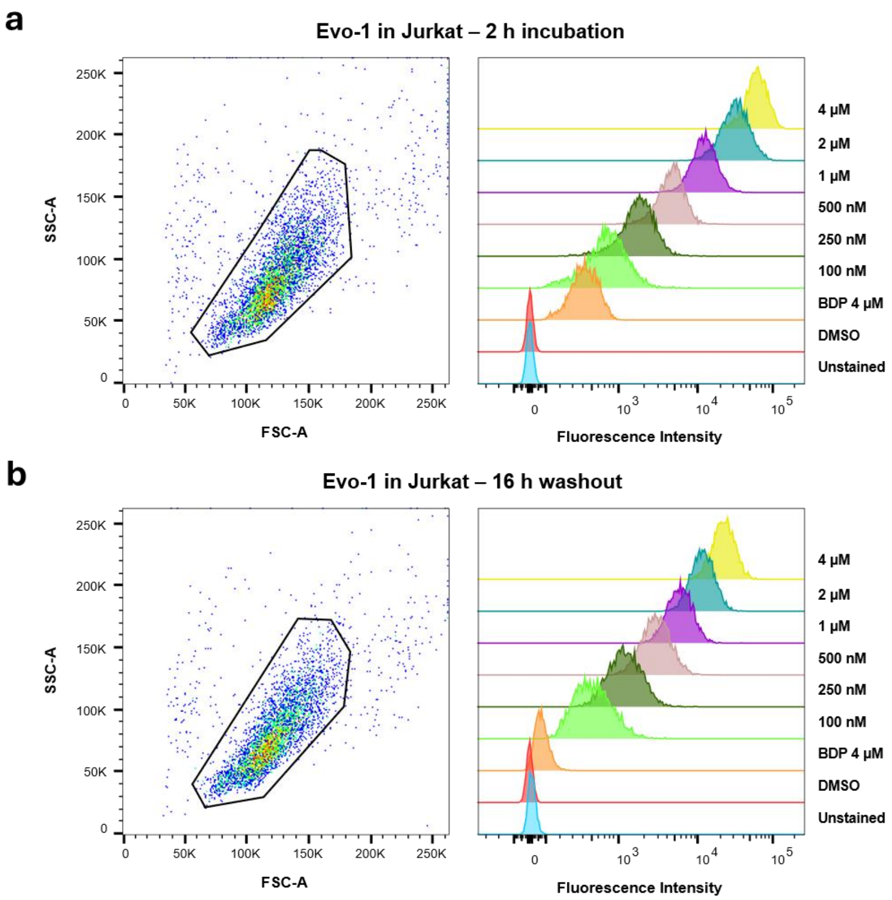

Figure S11. Cellular uptake of Evo-1 in Jurkat cells. Histogram of fluorescence intensities after, **a** 2 h incubation and **b** 2 h incubation followed by 16 h washout.

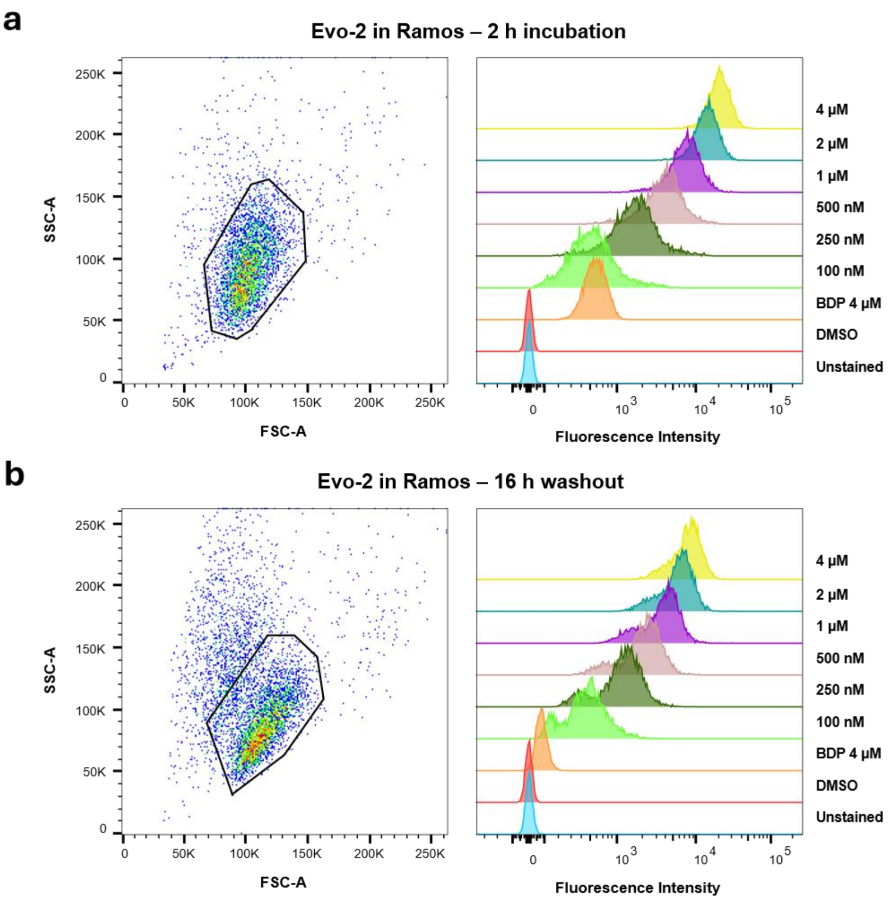

Figure S12. Cellular uptake of Evo-2 in Ramos cells. Histogram of fluorescence intensities after, **a** 2 h incubation and **b** 2 h incubation followed by 16 h washout.

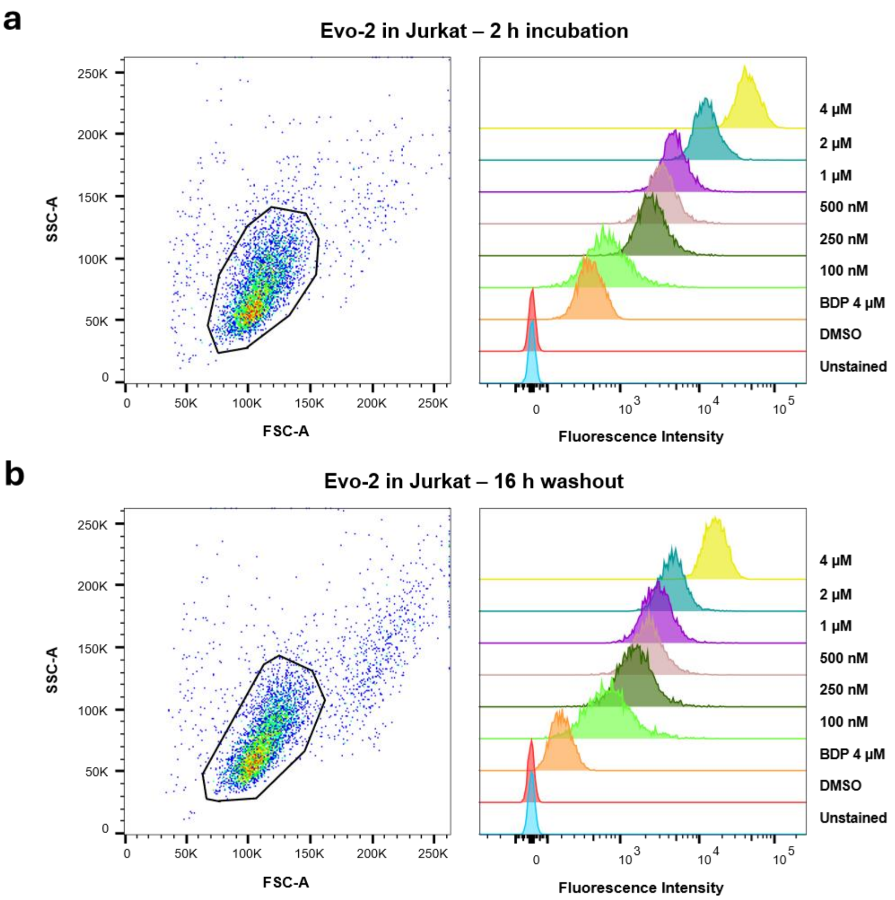

Figure S13. Cellular uptake of Evo-2 in Jurkat cells. Histogram of fluorescence intensities after, **a** 2 h incubation and **b** 2 h incubation followed by 16 h washout.

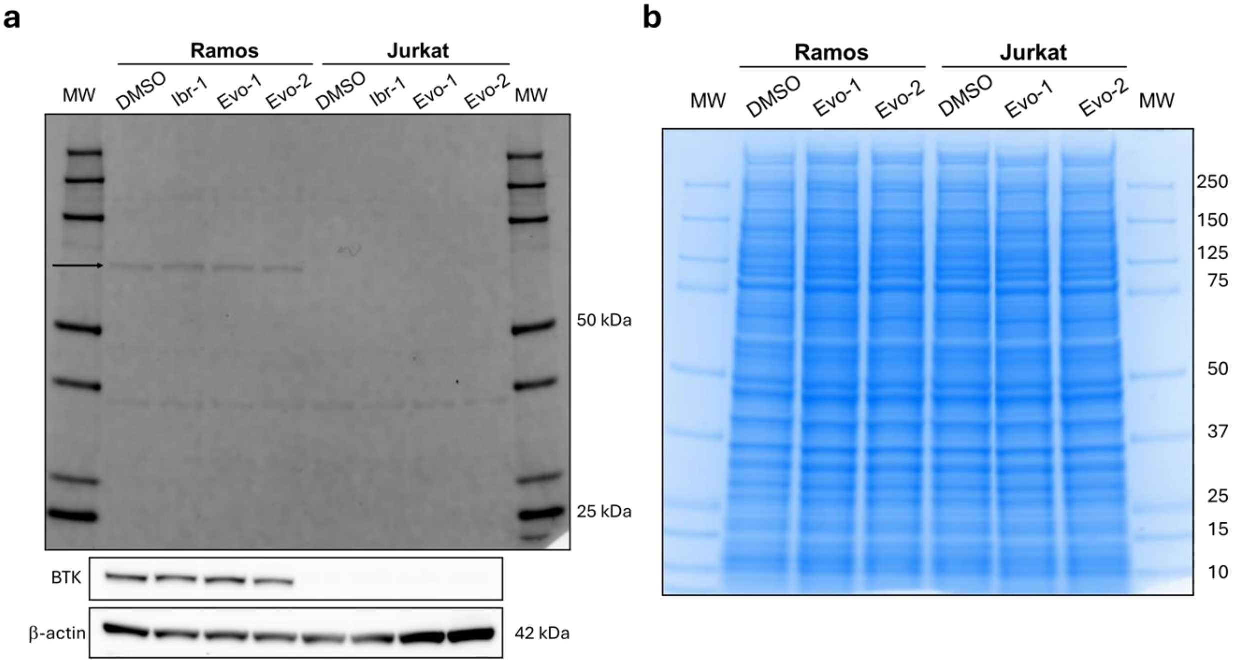

Figure S14. BTK expression in Ramos and Jurkat cells as assessed by immunoblotting. a Membrane fluorescence reading. Immunoblotting with mouse anti-BTK antibody followed by goat anti-mouse Alexa633 secondary antibody (top). Fluorescence detection of cross-coupled BTK with Alexa633 secondary antibody was performed on a ChemiDoc Imager (Alexa647 filter). The black arrow indicates the labelled BTK. Membranes were re-blotted with antibodies against BTK and β-actin and the presence of BTK was tested using the relevant antibodies by Western blot (bottom panel). MW fluorescent markers of 50 and 25 kDa are shown on the far left and right of the gel. **b** Total protein staining of the same gel with Colloidal blue. Size marker is shown on the far left and right.

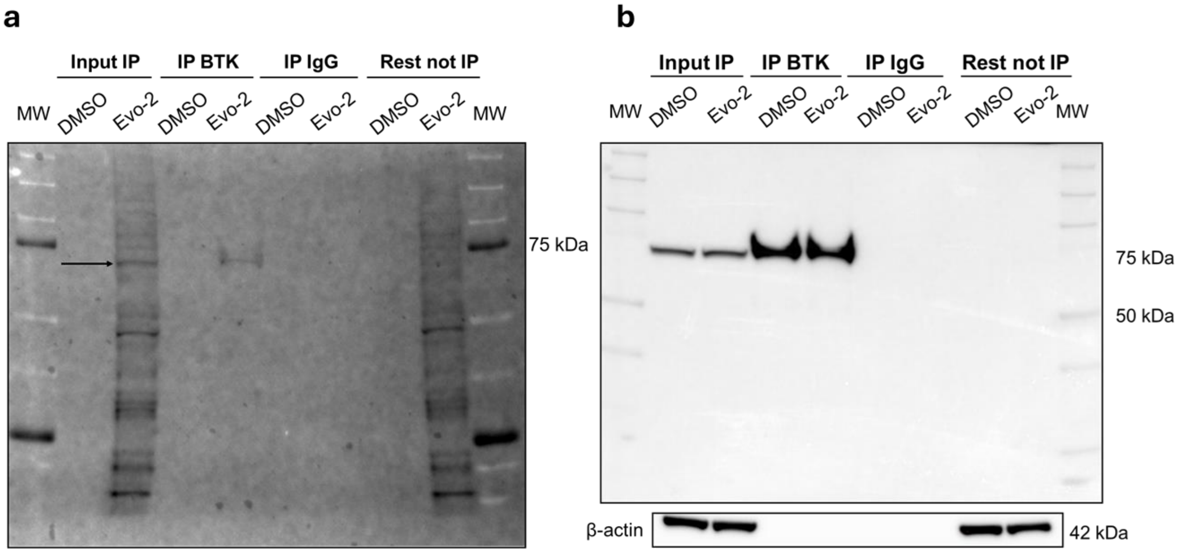

Figure S15. Immunoprecipitation of Evo-2-bound-BTK in Ramos cells after treatment with 4 µM Evo-2 for 4 h. a Detection of the immunoprecipitated BTK bound to **Evo-2** via fluorescence (ChemiDoc apparatus, Cy2 filter). The black arrow indicates the labelled BTK. MW fluorescent markers of 75 kDa are shown on the far left and right of the gel. **b** Membranes were immunoassayed with antibodies against BTK and β-actin respectively. MW size markers are shown on the far left and right of the membrane.

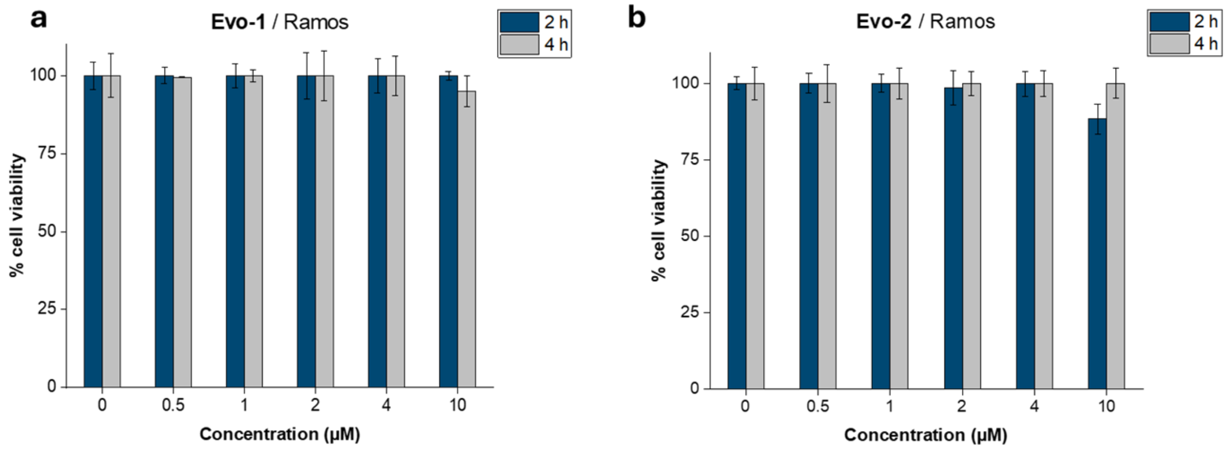

Figure S16. Cell cytotoxicity assay for probes Evo-1 and Evo-2 in Ramos cells. Ramos cells were treated with **Evo-1** and **Evo-2** at a range of concentrations 0-10 µM for 2 h (blue bars) and 4 h (grey bars) respectively. Data are presented as the mean ± SD (*n* = 3).

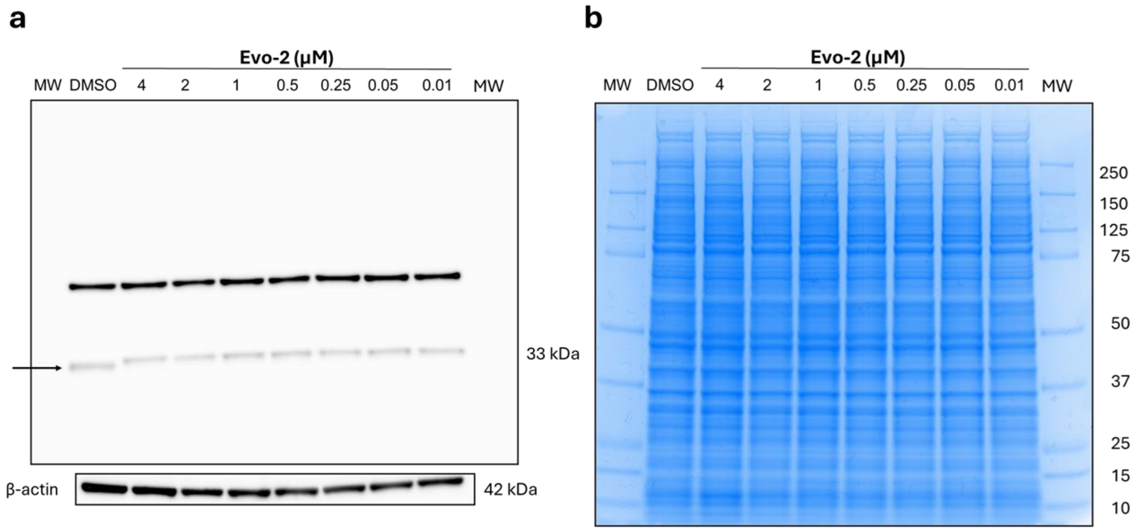

Figure S17. Binding of Evo-2 to SLC25A20 as suggested by immunoblotting. a Western blot analysis of Ramos whole-cell lysates after incubation with different concentrations of **Evo-2** for 2 h using rabbit monoclonal anti-SLC25A20 antibody (Abclonal, #ab23763). Note that the antibody for SLC25A20 gives also a nonspecific band at 50 kDa in addition to the specific band at around 30 kDa that corresponds to SLC25A20's MW (indicated by a black arrow). The slight difference in the protein's electrophoretic mobility

is probably attributed to the binding of **Evo-2** to SLC25A20, thus increasing the total MW compared to the DMSO control. **b** Total protein staining of the same gel with Colloidal blue. Size marker is shown on the far left and right.

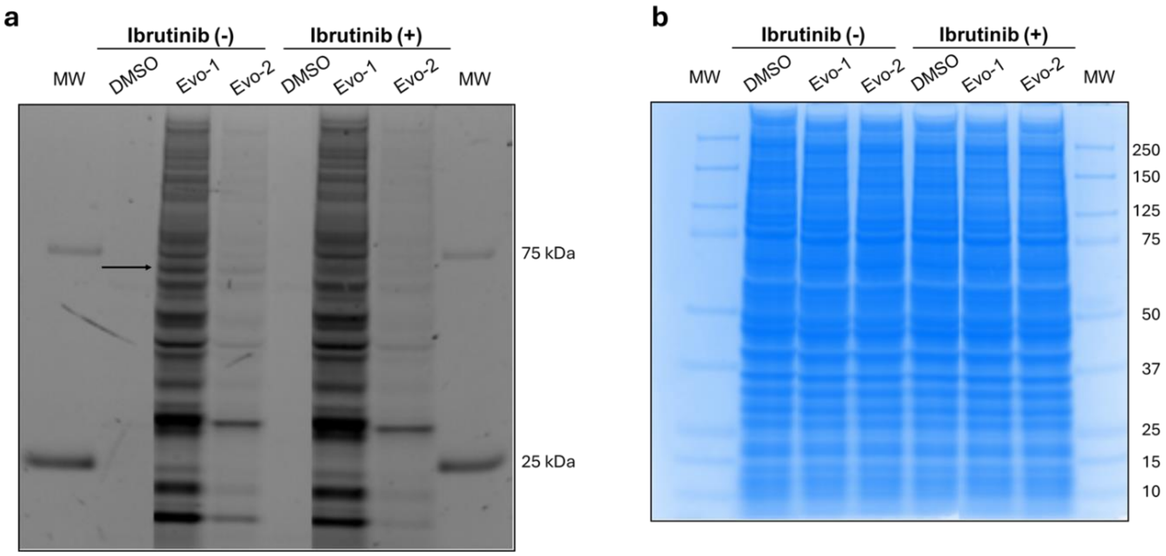

Figure S18. Competition experiment with ibrutinib. Pre-treatment of Ramos cells with 5 μM ibrutinib reduced the binding of fluorescent probes (500 nM) to BTK suggesting that the probes bind to the same site on cellular BTK as ibrutinib. **a** Cellular protein labelling profile of **Evo-1** and **Evo-2** in Ramos and Jurkat cells. Cells were pre-treated with ibrutinib (5 μM) for 30 min followed by incubation with 500 nM of compound or 0.1% DMSO for 2 h. The cells were washed, lysed, and cellular proteins were separated on SDS-PAGE gel under denatured and reduced conditions. The fluorescent signals of cellular proteins labelled by the probes were detected using a ChemiDoc apparatus (Alexa488 filter). The black arrow indicates the labelled BTK. MW fluorescent markers of 75 and 25 kDa are shown on the far left and right of the gel. **b** Total protein staining of the same gel with Colloidal blue. MW Size marker is shown on the far left and right of the gel.

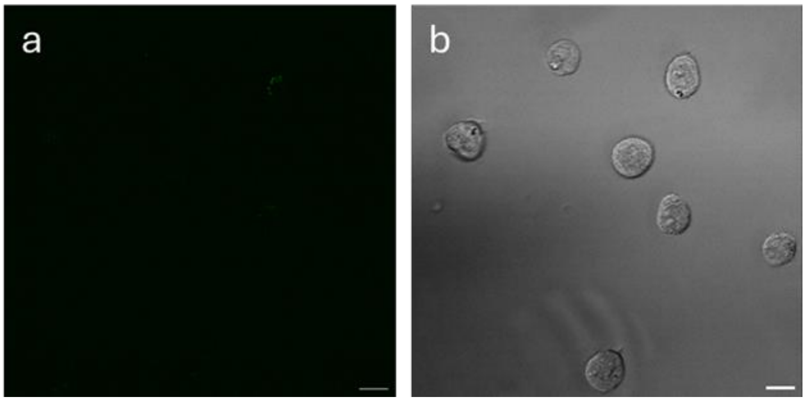

Figure S19. Fluorescence images of control Ramos cells without addition of Evo-2. a Fluorescence images were captured under probe's excitation wavelength (green, λ_{exc} = 488 nm). **b** Corresponding bright-field image. Scale bars: 10 µm.

Corrected Total Cell Fluorescence (CTCF) was calculated by using ImageJ[5], and according to the following equation:

CTCF = Integrated Density – (Area of selected cell × Mean fluorescence of background readings)

For the calculation of CTCF, three-dimensional images or Z-stacks were reduced to bidimensional images by arithmetic sum of the fluorescence intensity of the different slices. CTCF was expressed as the average of measuring various cells. As for the background readings, equal number of regions of interest (ROIs) were drawn in the corresponding pictures.

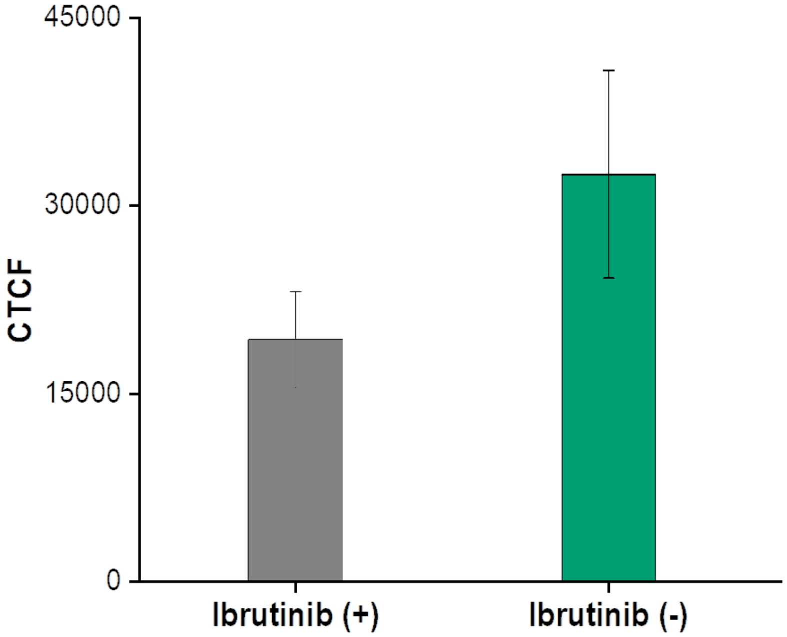

Figure S20. Competition experiment with ibrutinib - differences in fluorescence intensities as assessed by CTCF analysis. Ramos cells were pretreated with (grey bar) or without ibrutinib (green bar) (5 µM) for 30 minutes followed by incubation with **Evo-2** (1 µM) for 2 h. CTCF was expressed as the average of measuring various cells.

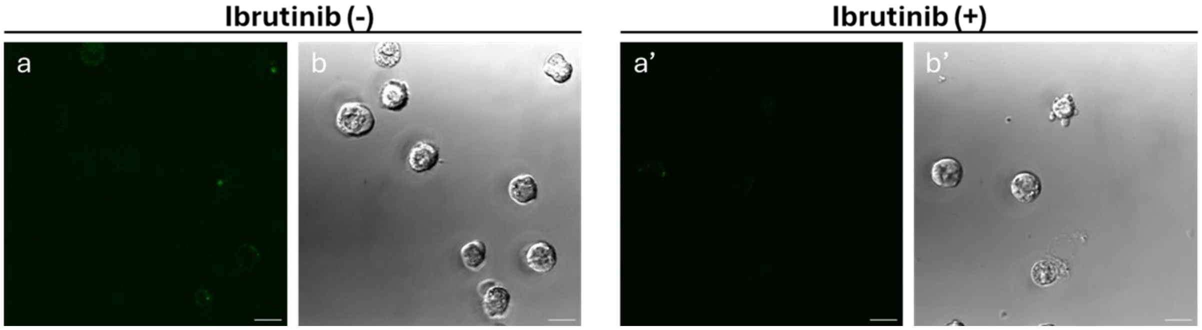

Figure S21. Fluorescence images of control Ramos cells after incubation with or without Ibrutinib (5 μM) and without addition of Evo-2. a, a' Fluorescence images were captured under probe's excitation wavelength (green, λ_{exc} = 488 nm). **b, b'** Corresponding bright-field images. Scale bars: 10 μm.

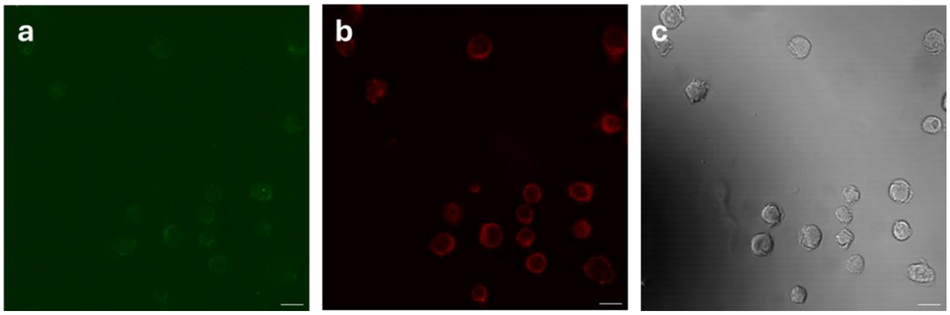

Figure S22. Fluorescence images of control fixed Ramos cells stained with anti-BTK/Alexa633 antibodies. Ramos cells were treated with DMSO for 2 h. After fixation, permeabilization, and antibody staining, cells were imaged under, **a** Probe's excitation wavelength (green, λ_{exc} = 488 nm), **b** antibodies' excitation wavelength wavelength (red, λ_{exc} = 633 nm). **c** Corresponding bright-field image. Scale bars: 10 μm.

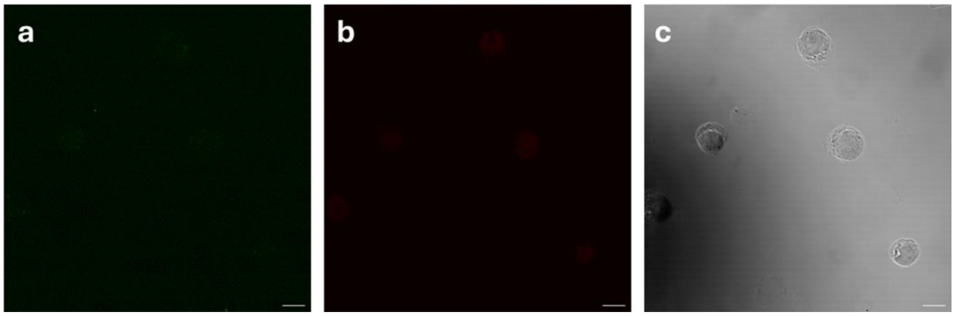

Figure S23. Fluorescence images of control fixed Ramos cells stained with isotype control/Alexa633 antibodies. Ramos cells were treated with DMSO for 2 h. After fixation, permeabilization, and antibody staining, cells were imaged under, **a** Probe's excitation wavelength (green, λ_{exc} = 488 nm), **b** antibodies' excitation wavelength wavelength (red, λ_{exc} = 633 nm). **c** Corresponding bright-field image. Scale bars: 10 μm.

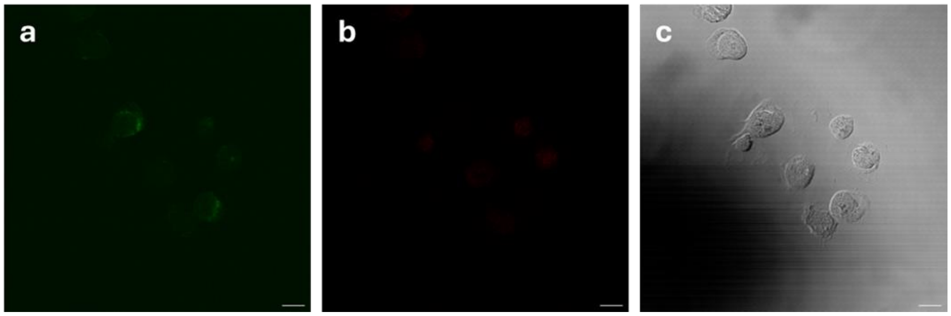

Figure S24. Fluorescence images of control fixed Ramos cells stained with isotype control/Alexa633 antibodies. Ramos cells were treated with 1 μM **Evo-2** for 2 h. After fixation, permeabilization, and antibody staining, cells were imaged under, **a** Probe's excitation wavelength (green, λ_{exc} = 488 nm), **b** antibodies' excitation wavelength (red, λ_{exc} = 633 nm). **c** Corresponding bright-field image. Scale bars: 10 μm.

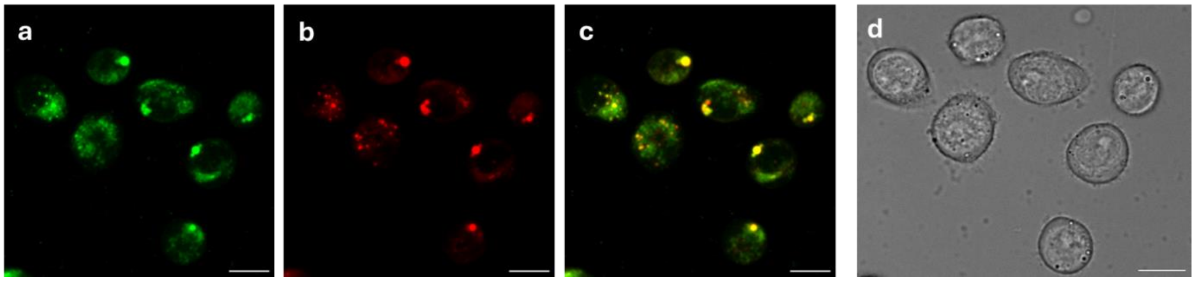

Figure S25. Fluorescence images of Ramos cells treated with Evo-2 and LysoTracker DeepRed. Ramos cells were treated with 1 μM Evo-2 for 2 h, followed by treatment with LysoTracker Deep Red (50 nM, 30 min) and cells were imaged under, **a** Probe's excitation wavelength (green, λ_{exc} = 488 nm), **b** Lysotracker's excitation wavelength (red, λ_{exc} = 639 nm). **c** Corresponding bright-field image. Scale bars: 10 μm.

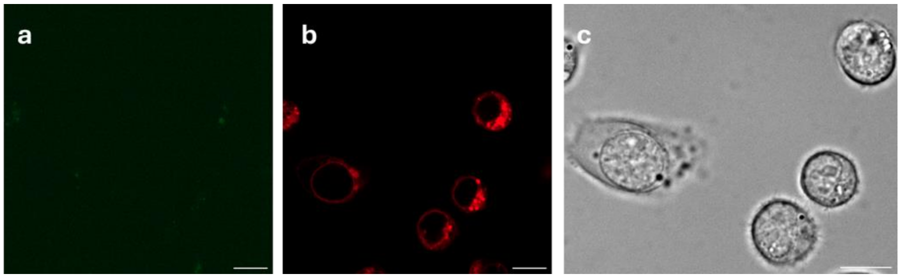

Figure S26. Fluorescence images of control Ramos cells treated with DMSO and LysoTracker DeepRed. Ramos cells were treated with DMSO for 2 h, followed by treatment with LysoTracker Deep Red (50 nM, 30 min) and cells were imaged under, **a** Probe's excitation wavelength (green, λ_{exc} = 488 nm), **b** Lysotracker's excitation wavelength (red, λ_{exc} = 639 nm). **c** Corresponding bright-field image. Scale bars: 10

NMR Spectra

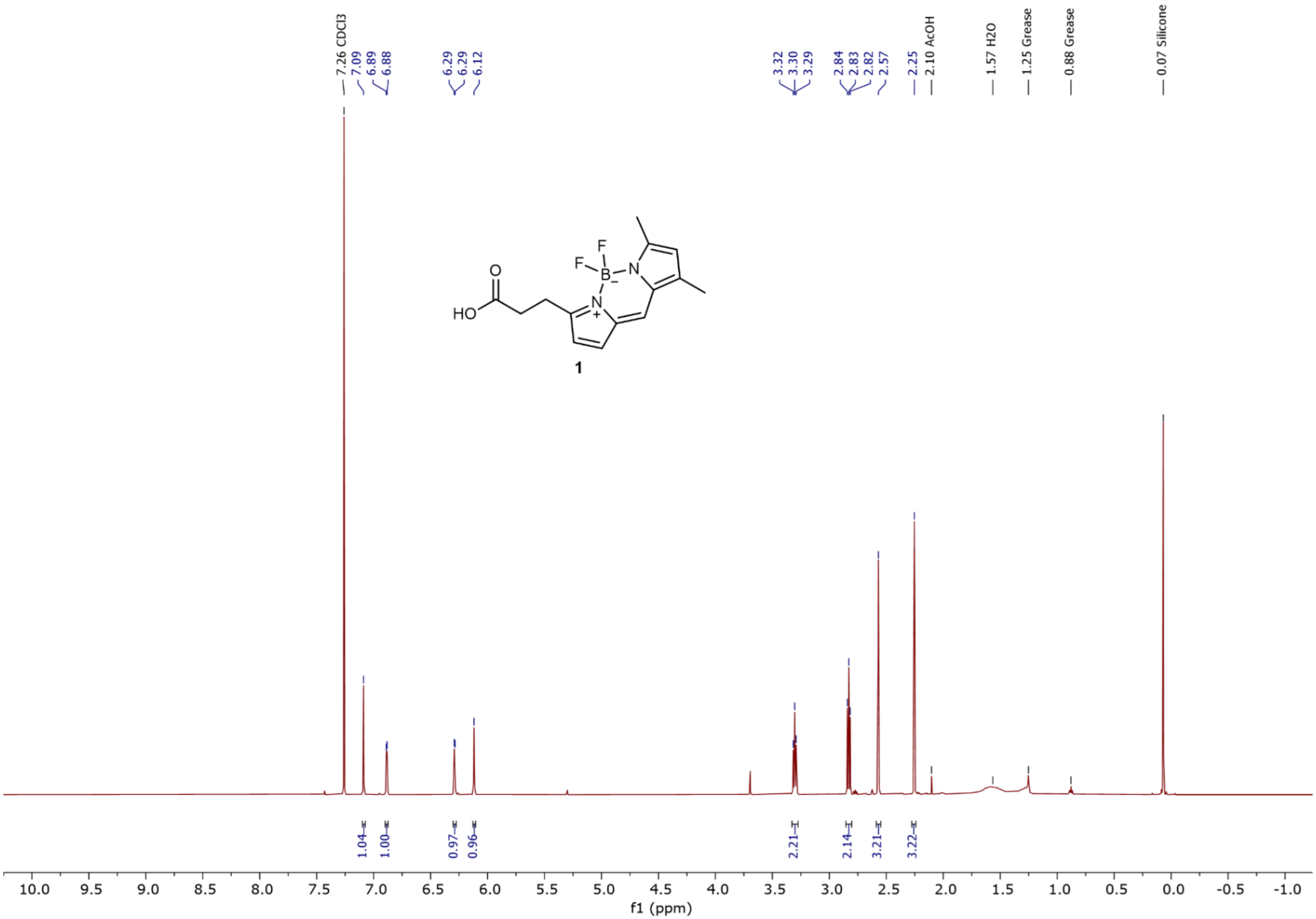

Figure S27. ^{1}H NMR spectrum of compound **1** (600 MHz, $CDCl_3$).

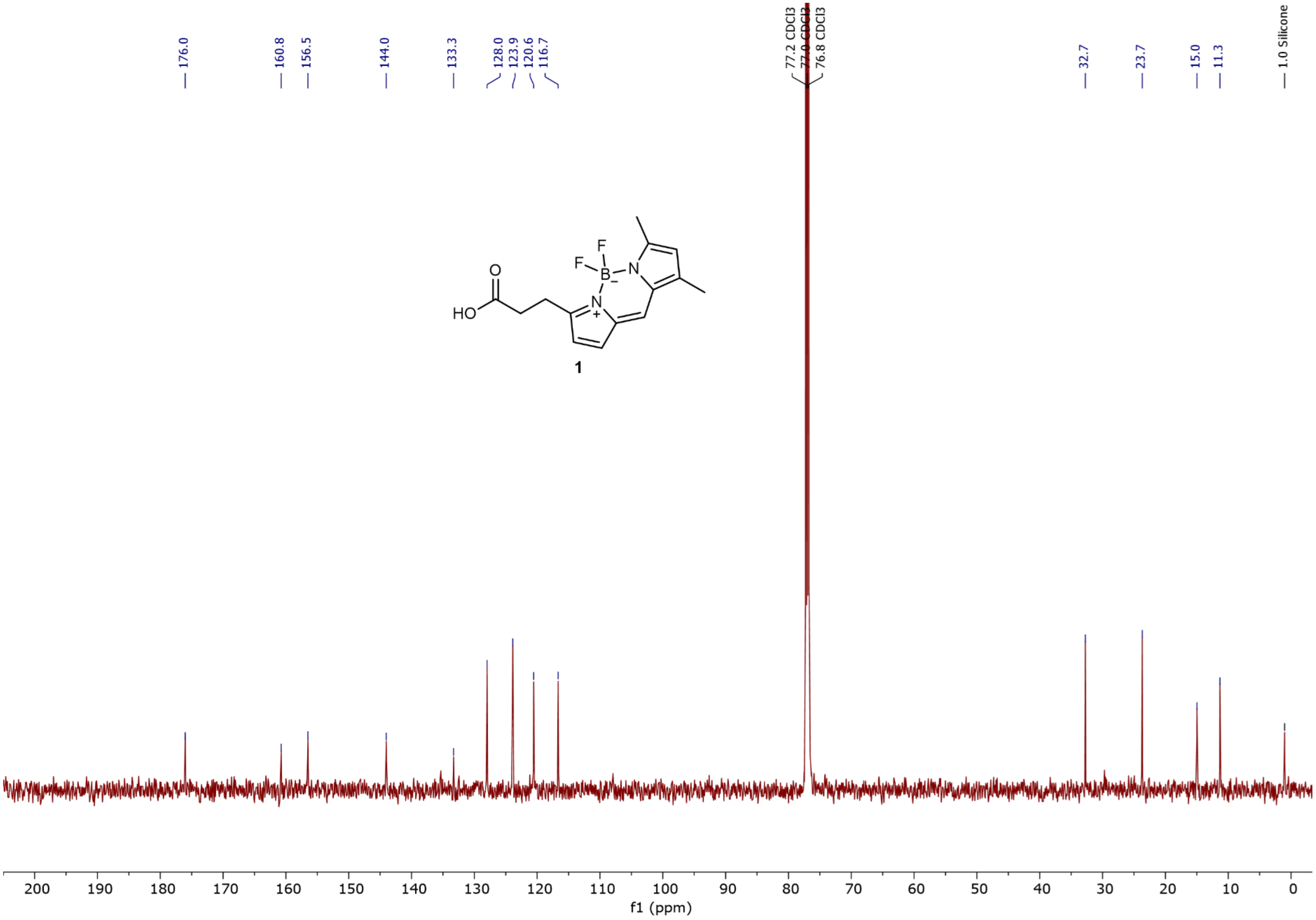

Figure S18. $^{13}C\{^{1}H\}$ NMR spectrum of compound **1** (151 MHz, $CDCl_3$).

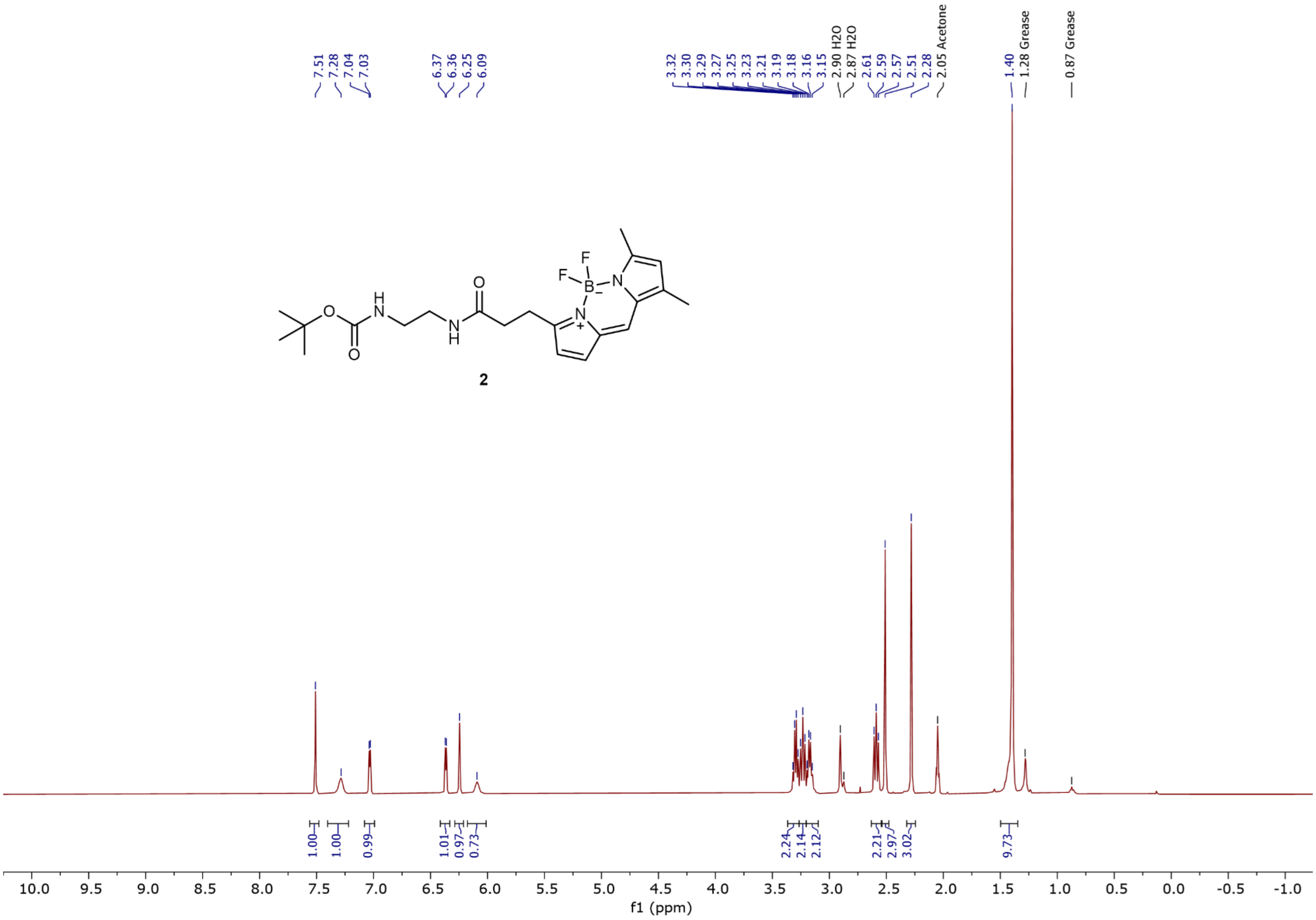

Figure S29. ^{1}H NMR spectrum of compound **2** (400 MHz, $(CD_3)_2CO$).

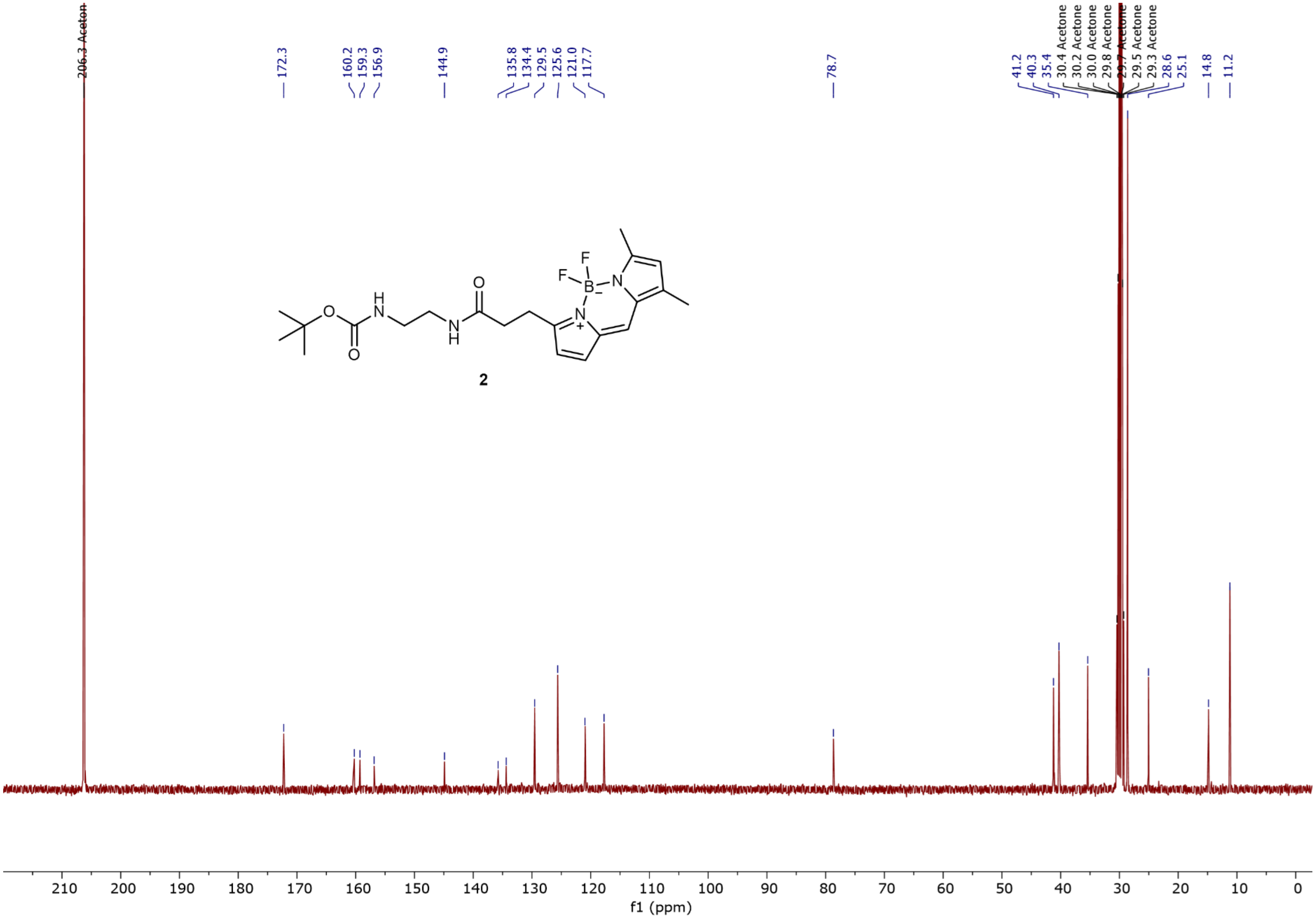

Figure S30. $^{13}C\{^{1}H\}$ NMR spectrum of compound **2** (101 MHz, $(CD_3)_2CO$).

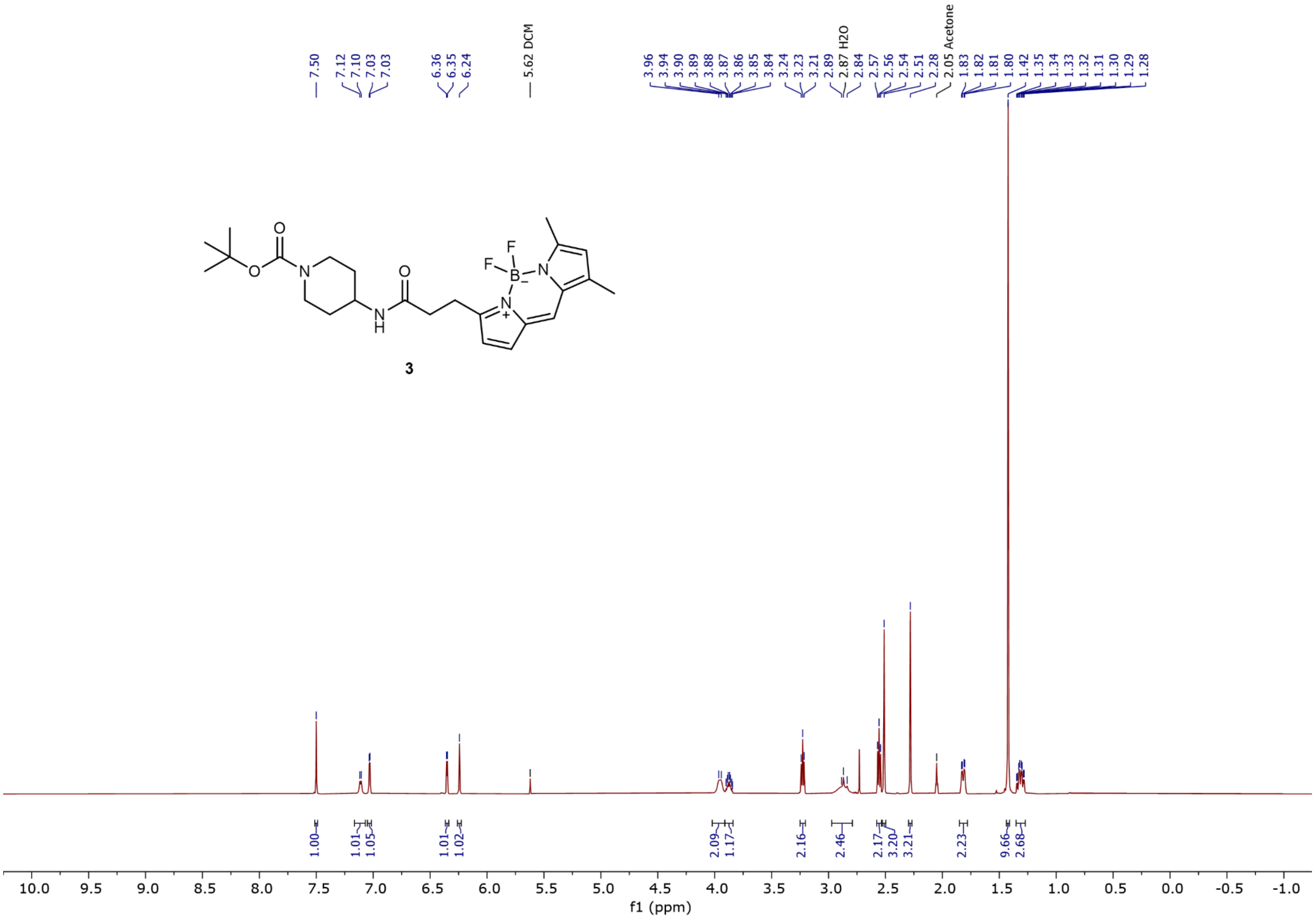

Figure S31. ^{1}H NMR spectrum of compound **3** (600MHz, $(CD_3)_2CO$).

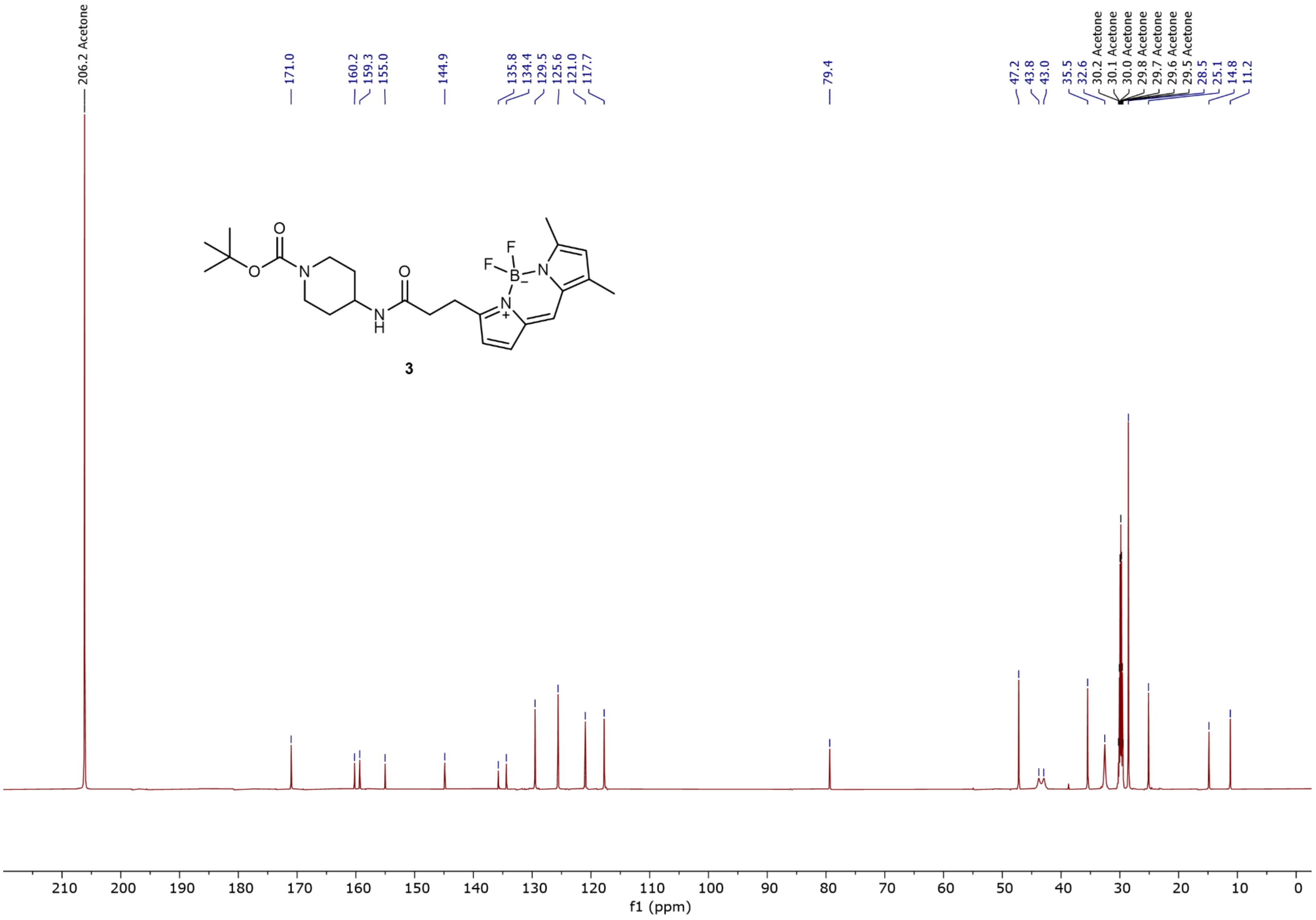

Figure S32. $^{13}C\{^{1}H\}$ NMR spectrum of compound **3** (151 MHz, $(CD_3)_2CO$).

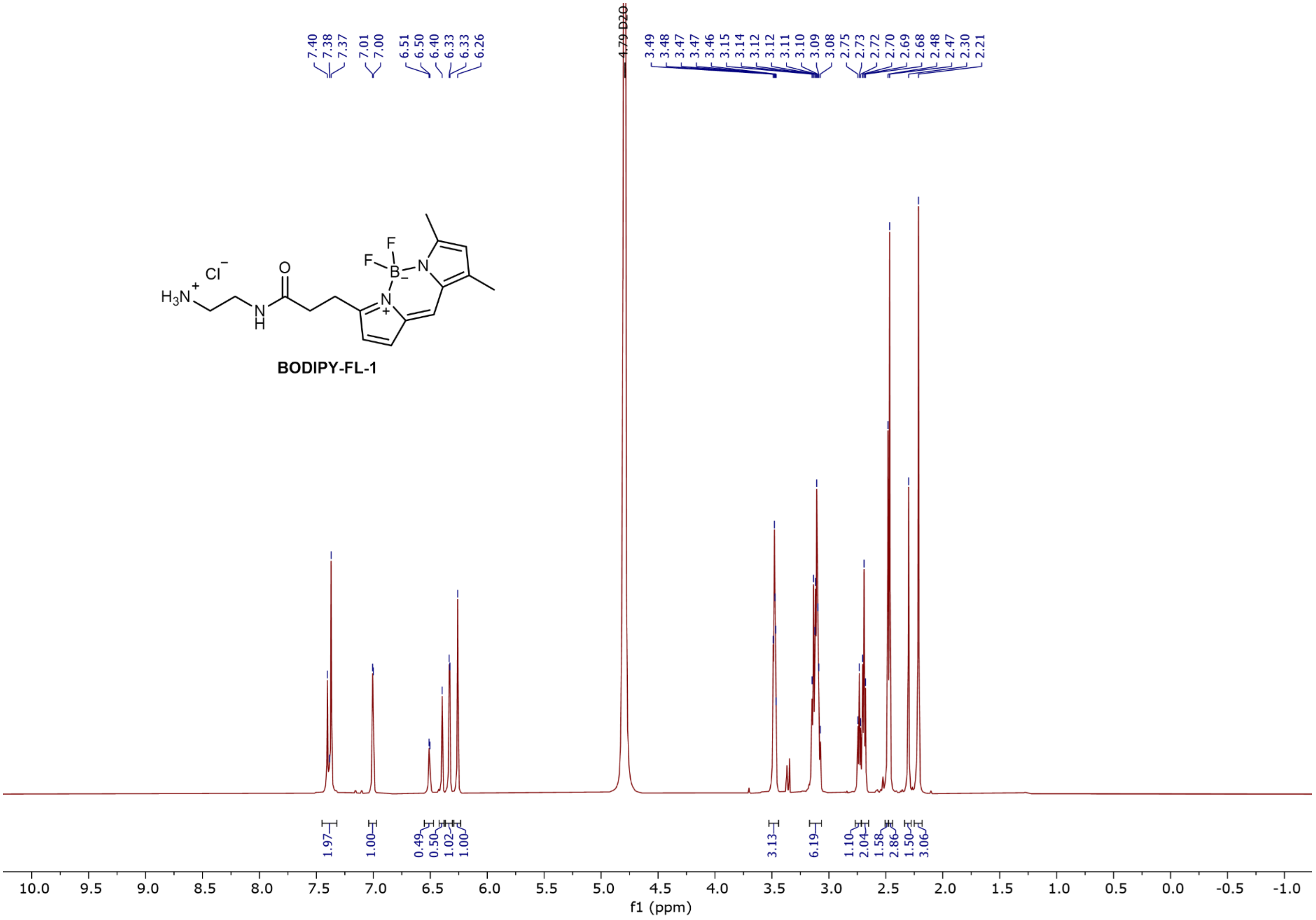

Figure S33. ^{1}H NMR spectrum of compound **BODIPY-FL-1** (600MHz, D_2O).

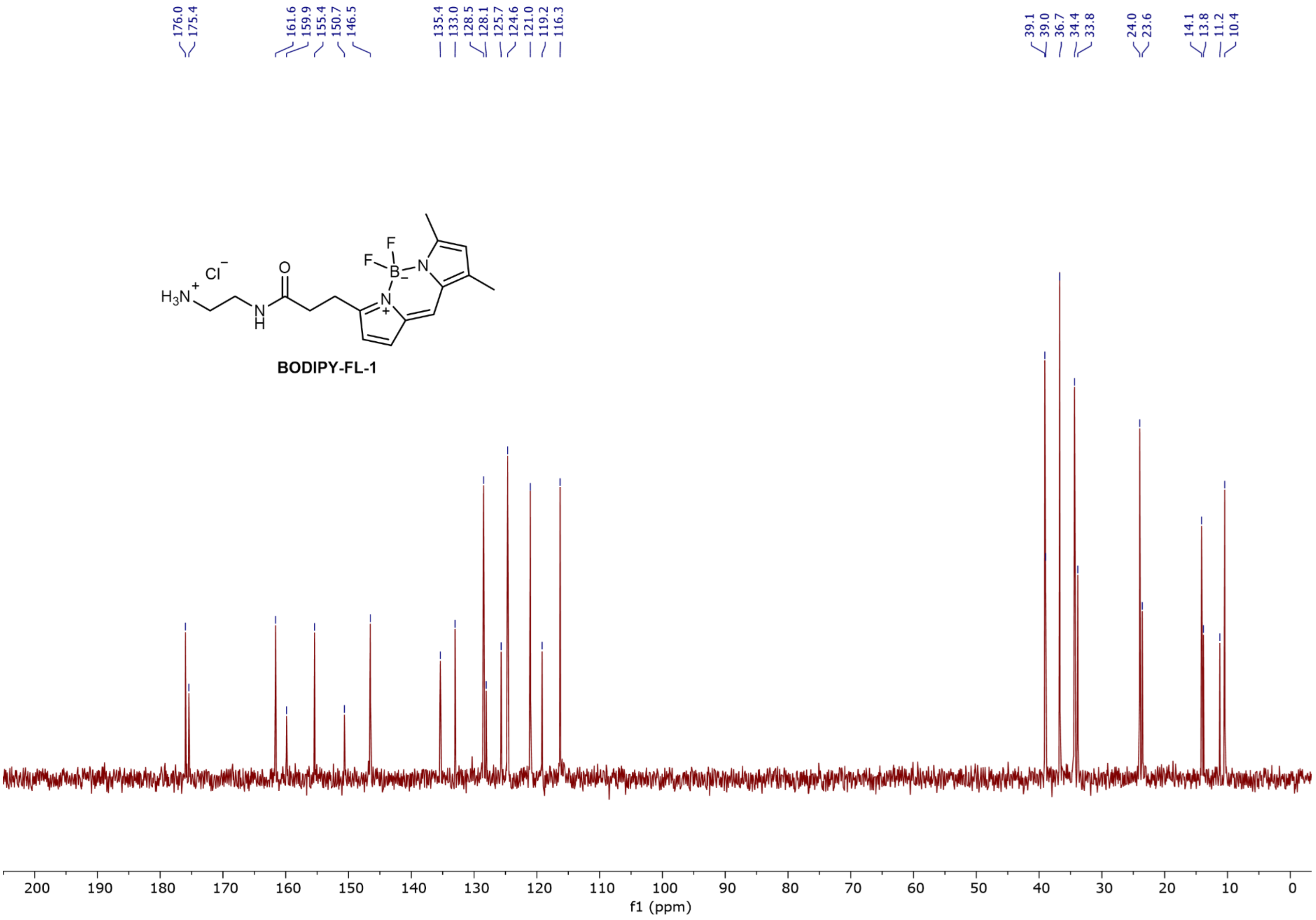

Figure S34. $^{13}C\{^{1}H\}$ NMR spectrum of compound **BODIPY-FL-1** (151 MHz, D_2O).

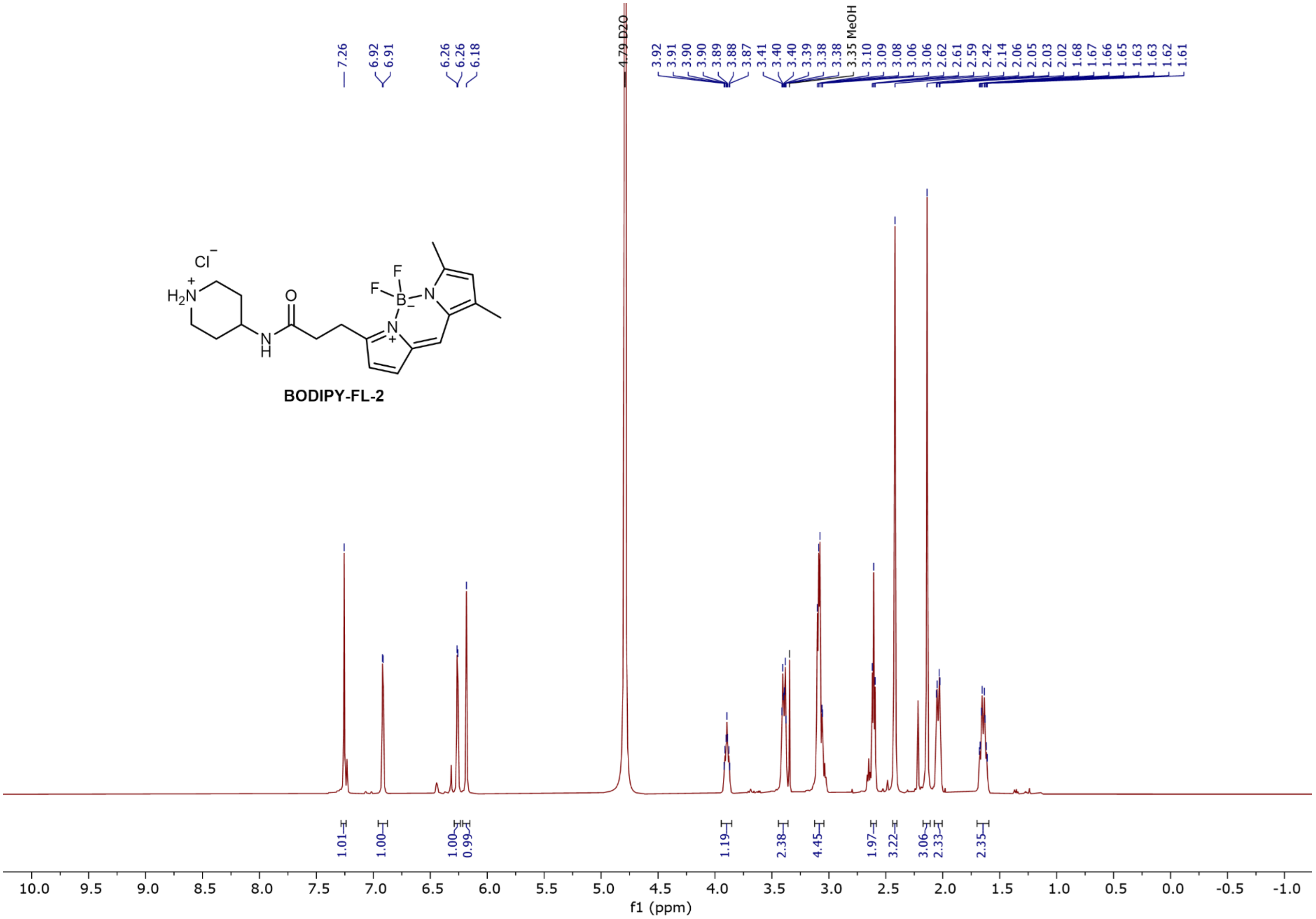

Figure S35. ^{1}H NMR spectrum of **BODIPY-FL-2** (600MHz, D_2O).

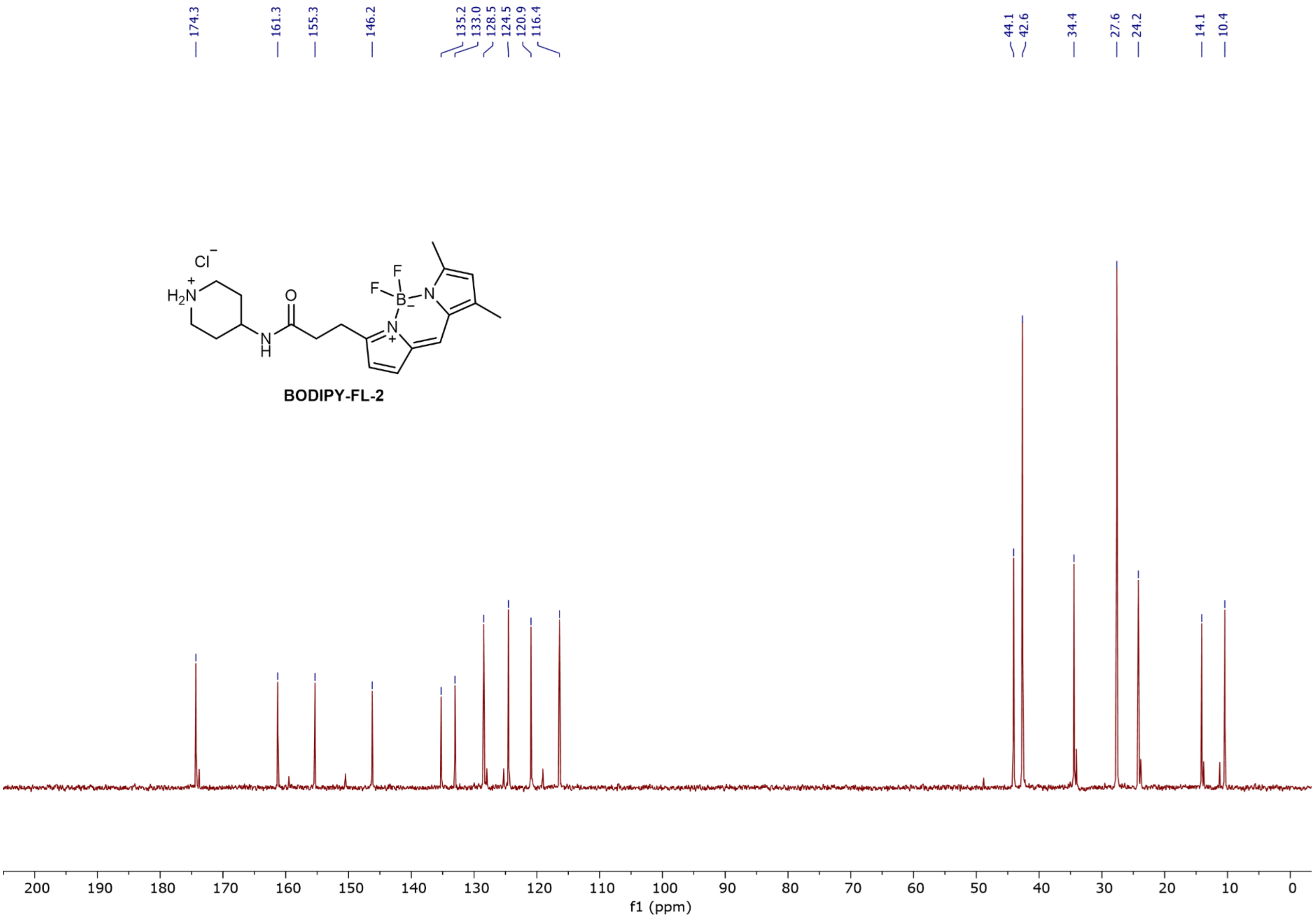

Figure S36. $^{13}C\{^{1}H\}$ NMR spectrum of **BODIPY-FL-2** (151 MHz, D_2O).

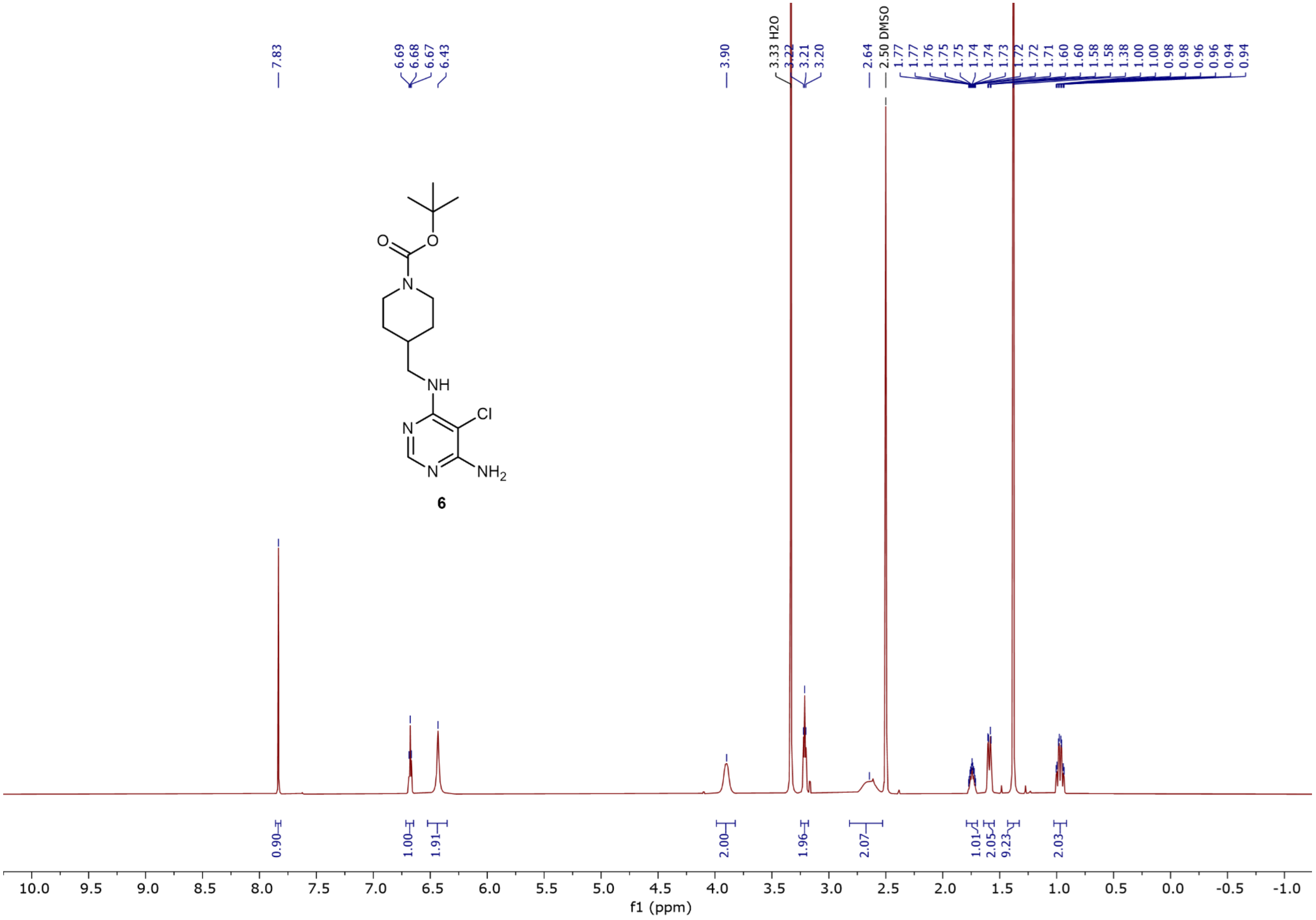

Figure S37. ^{1}H NMR spectrum of compound **6** (600MHz, $(CD_3)_2SO$).

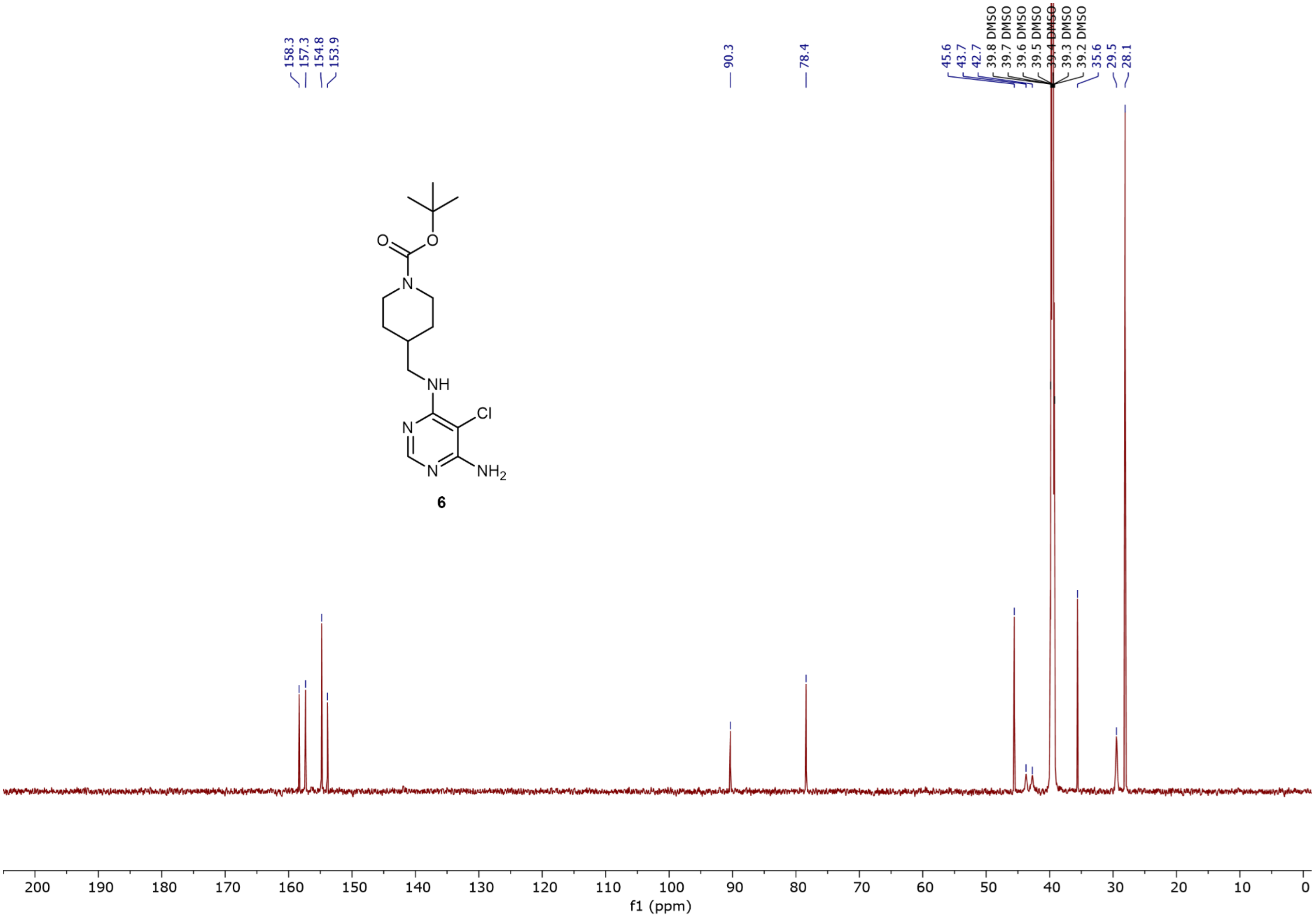

Figure S38. $^{13}C\{^{1}H\}$ NMR spectrum of compound **6** (201 MHz, $(CD_3)_2SO$).

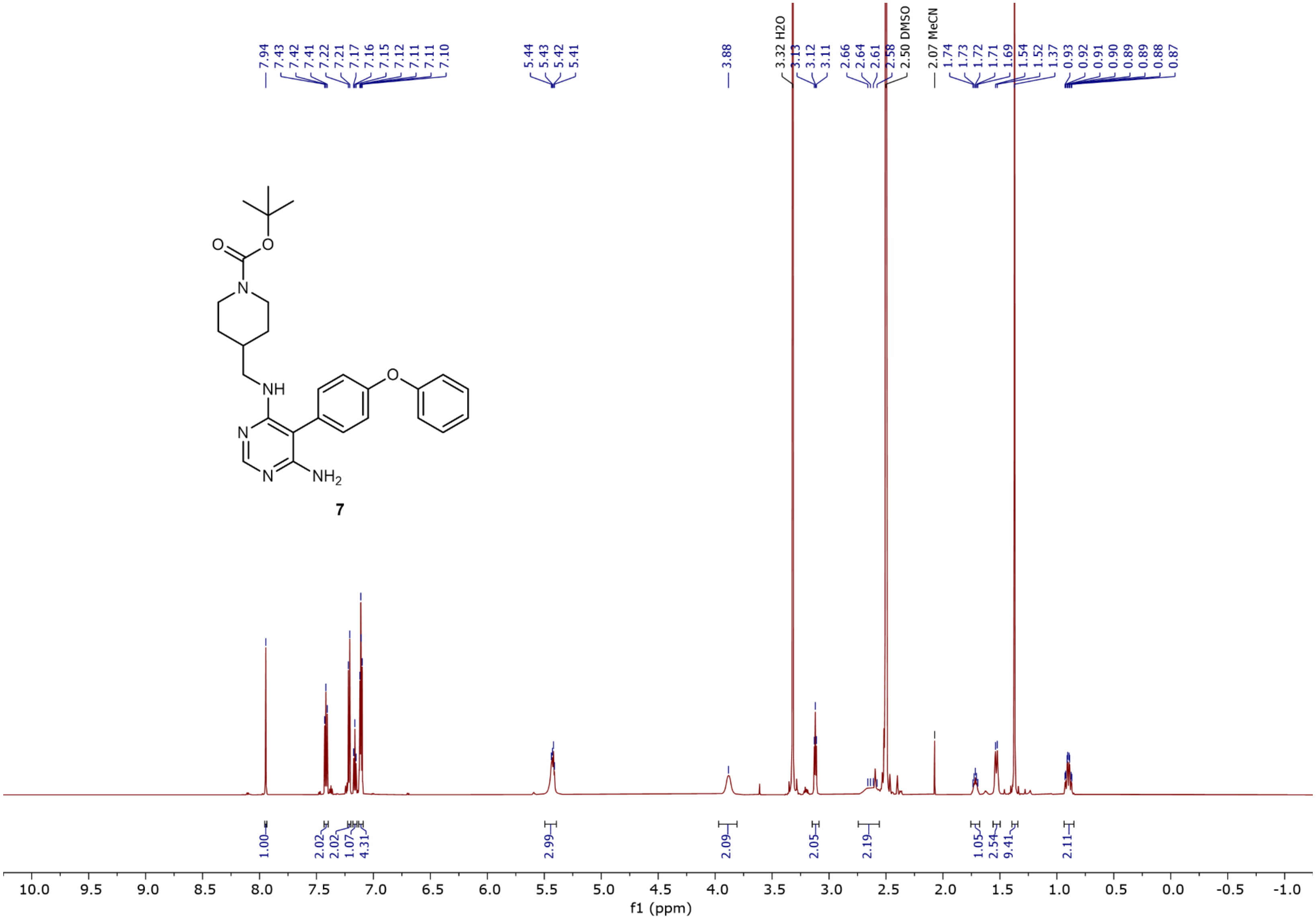

Figure S39. ^{1}H NMR spectrum of compound **7** (700MHz, $(CD_3)_2SO$).

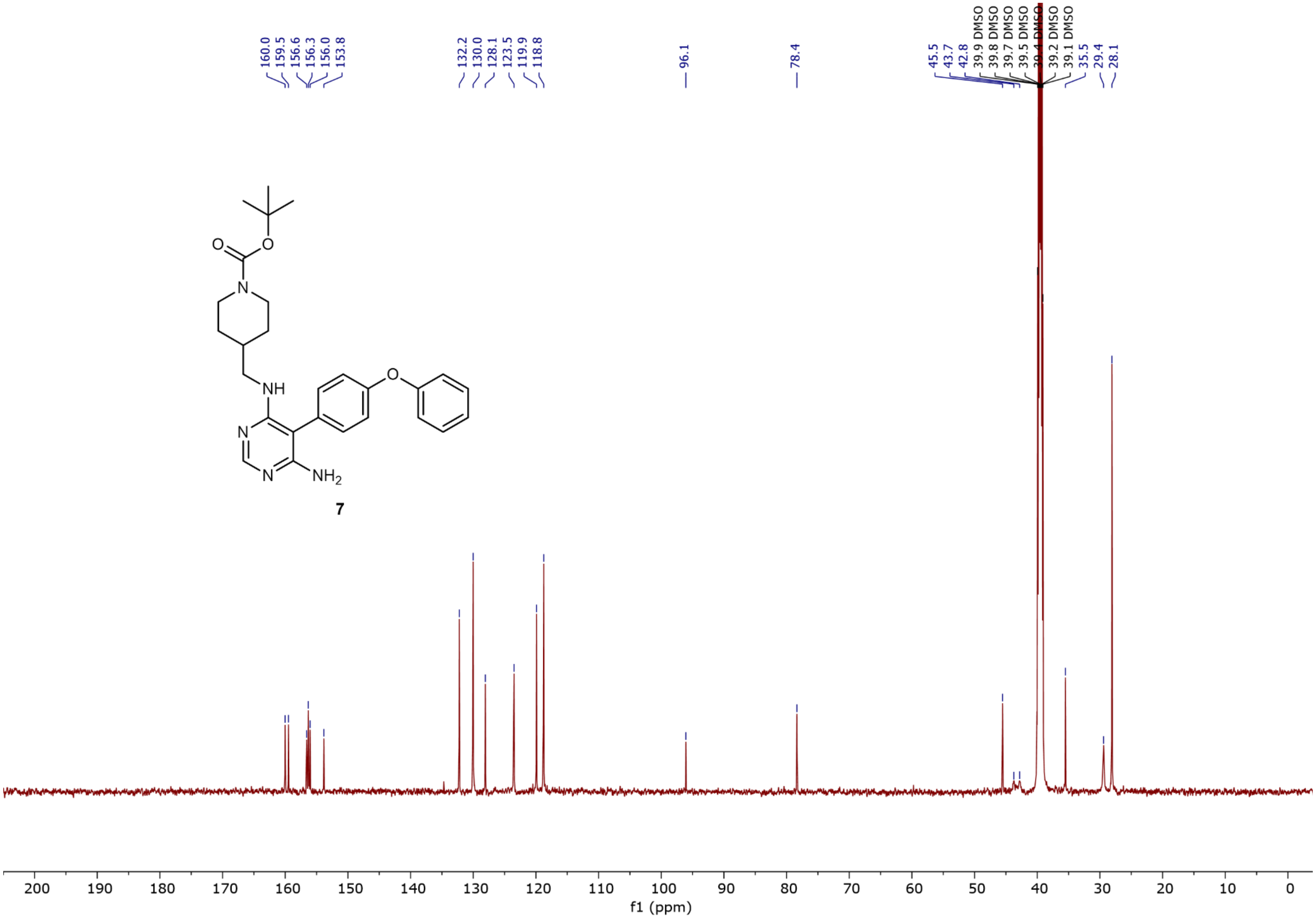

Figure S40. $^{13}C\{^1H\}$ NMR spectrum of compound **7** (151 MHz, $(CD_3)_2SO$).

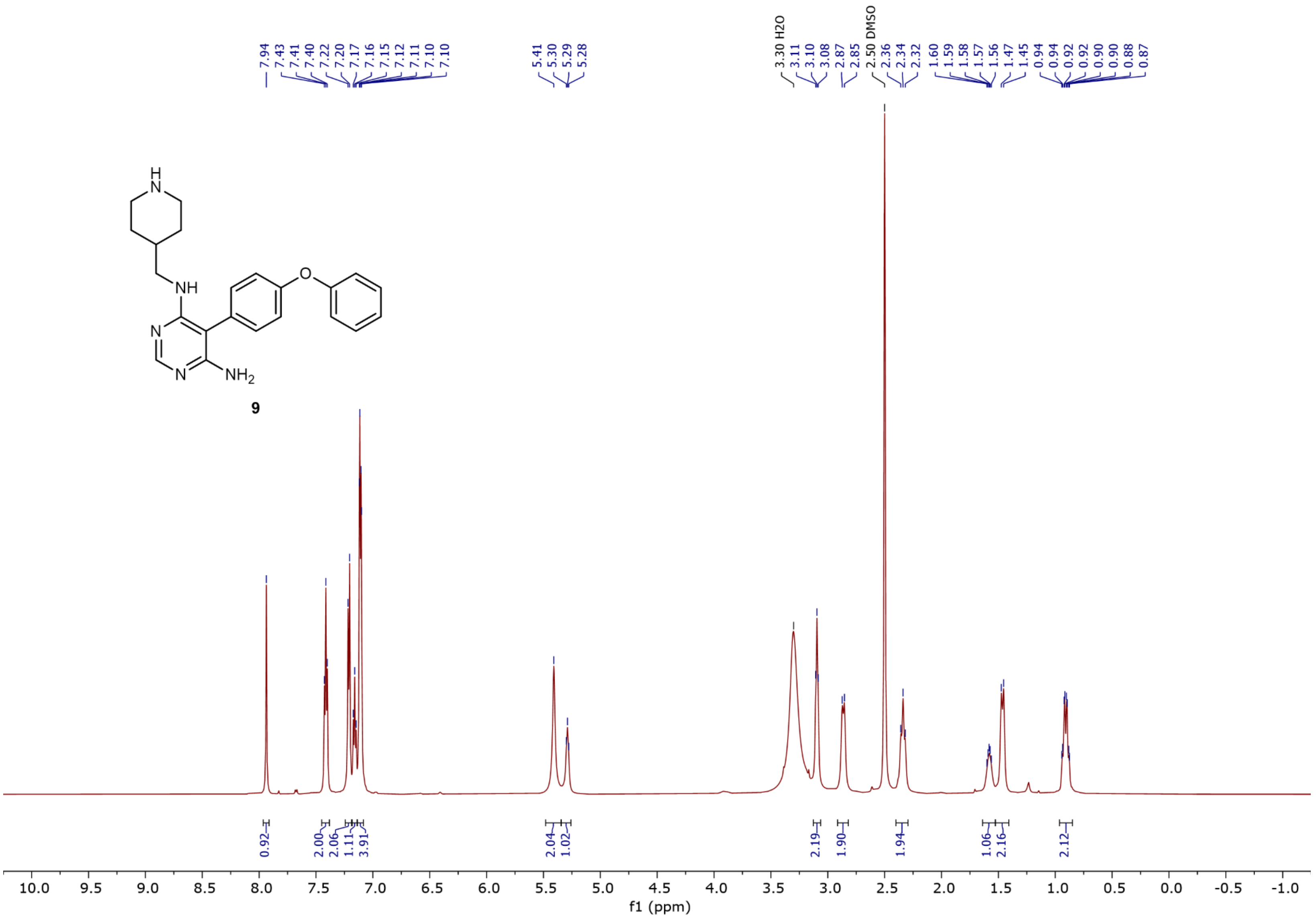

Figure S41. ^{1}H NMR spectrum of compound **9** (600MHz, $(CD_3)_2SO$).

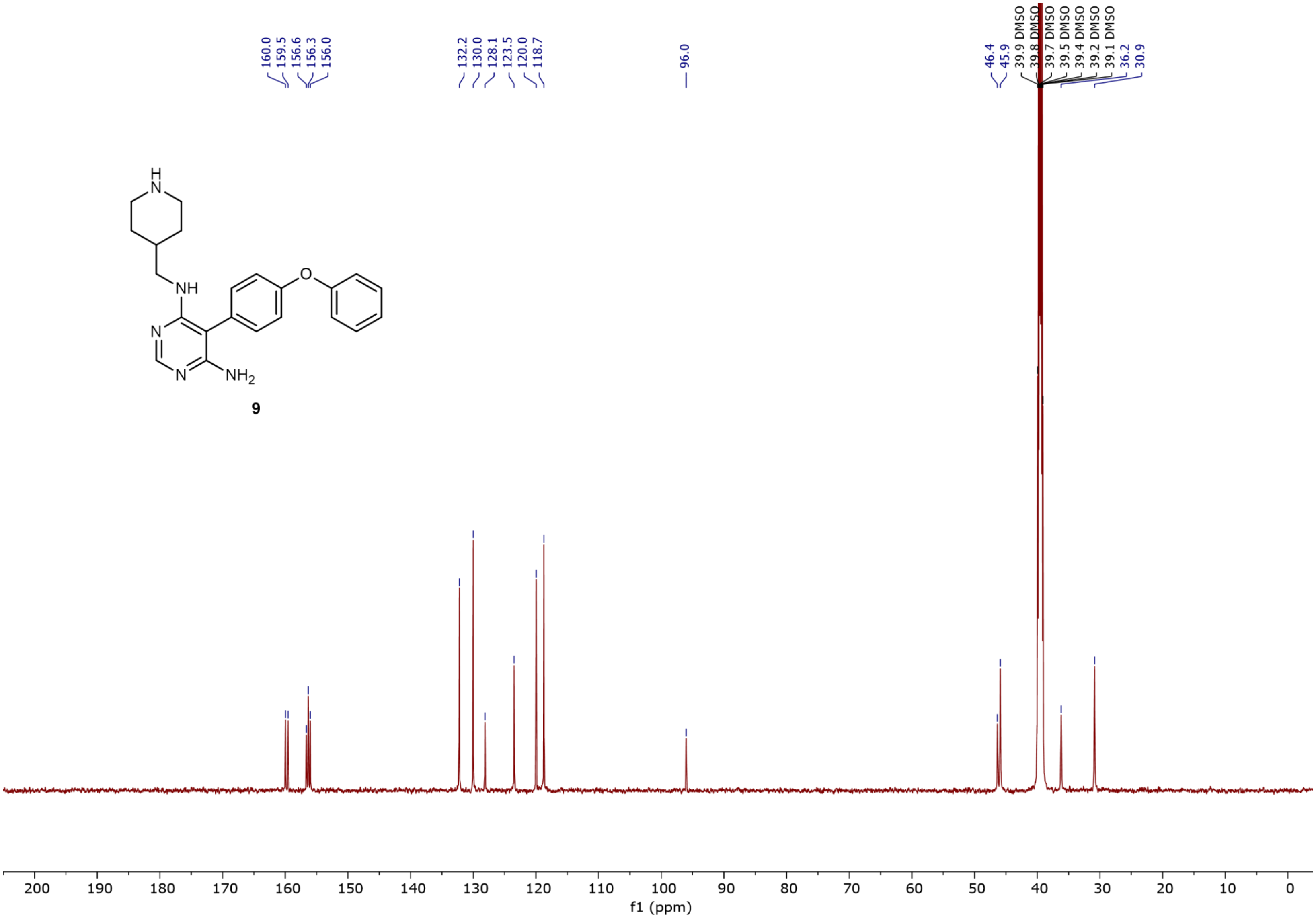

Figure S42. $^{13}C\{^{1}H\}$ NMR spectrum of compound **9** (151 MHz, $(CD_3)_2SO$).

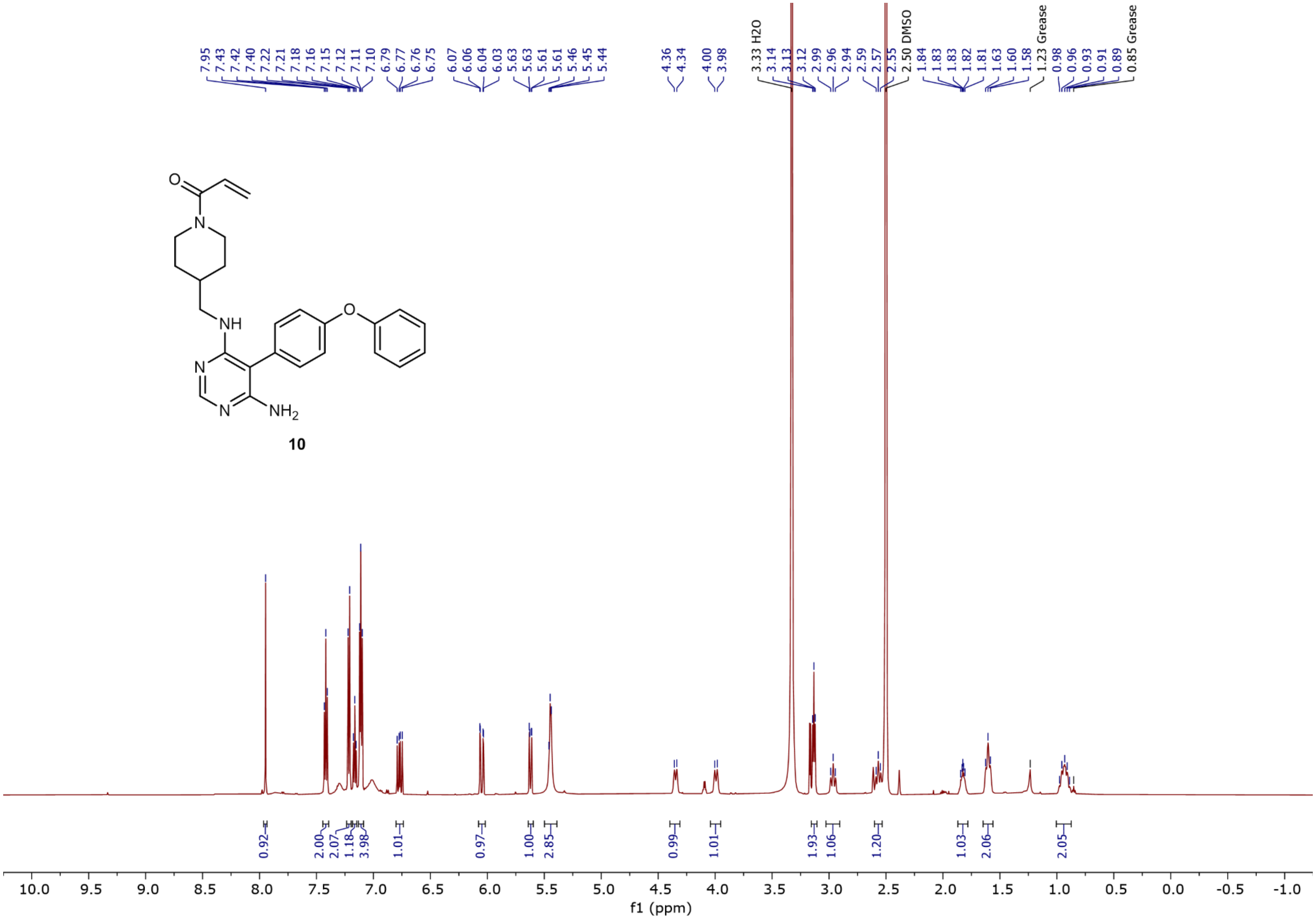

Figure S43. ^{1}H NMR spectrum of compound **10** (600MHz, $(CD_3)_2SO$).

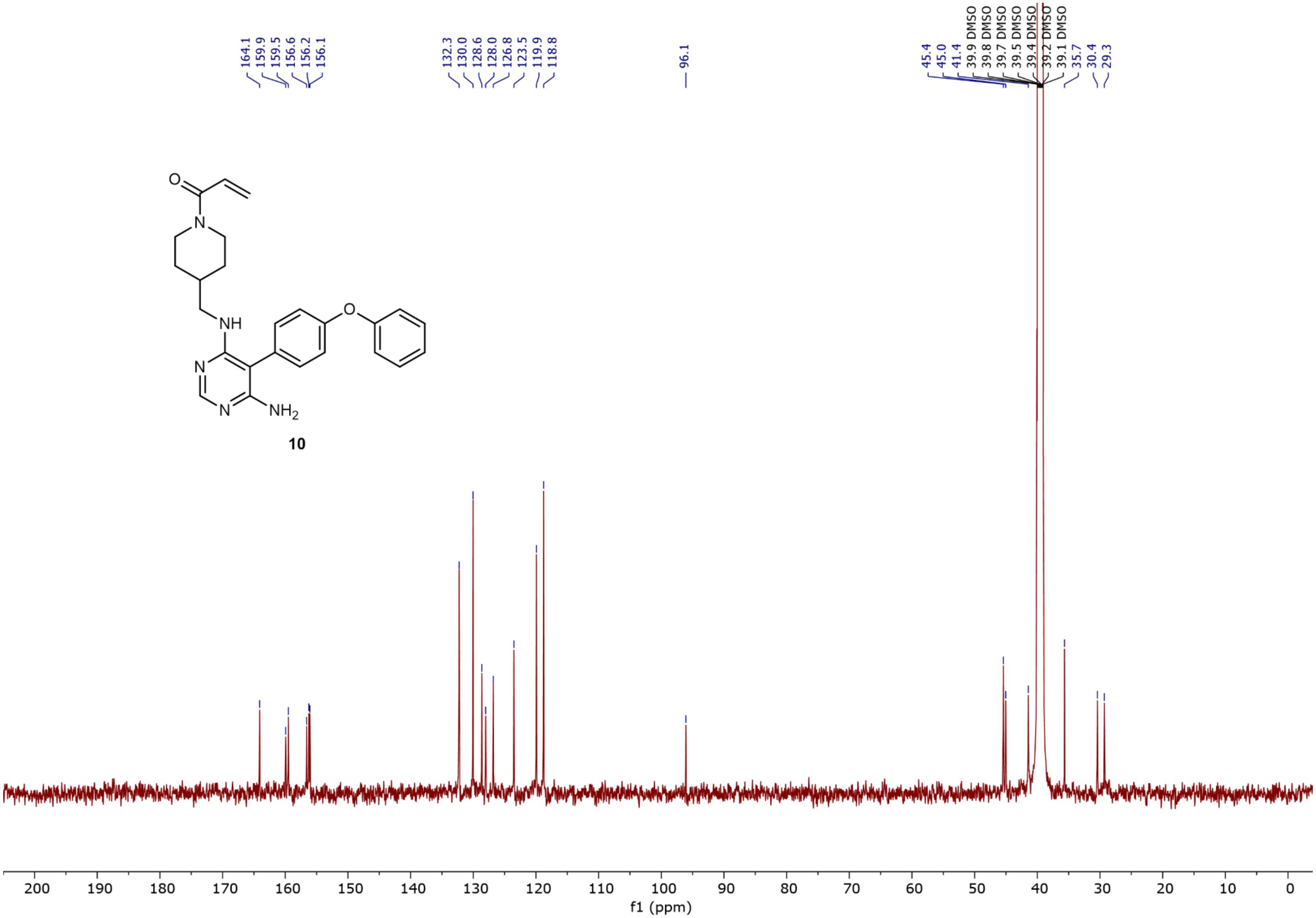

Figure S44. $^{13}C\{^{1}H\}$ NMR spectrum of compound **10** (151 MHz, $(CD_3)_2SO$).

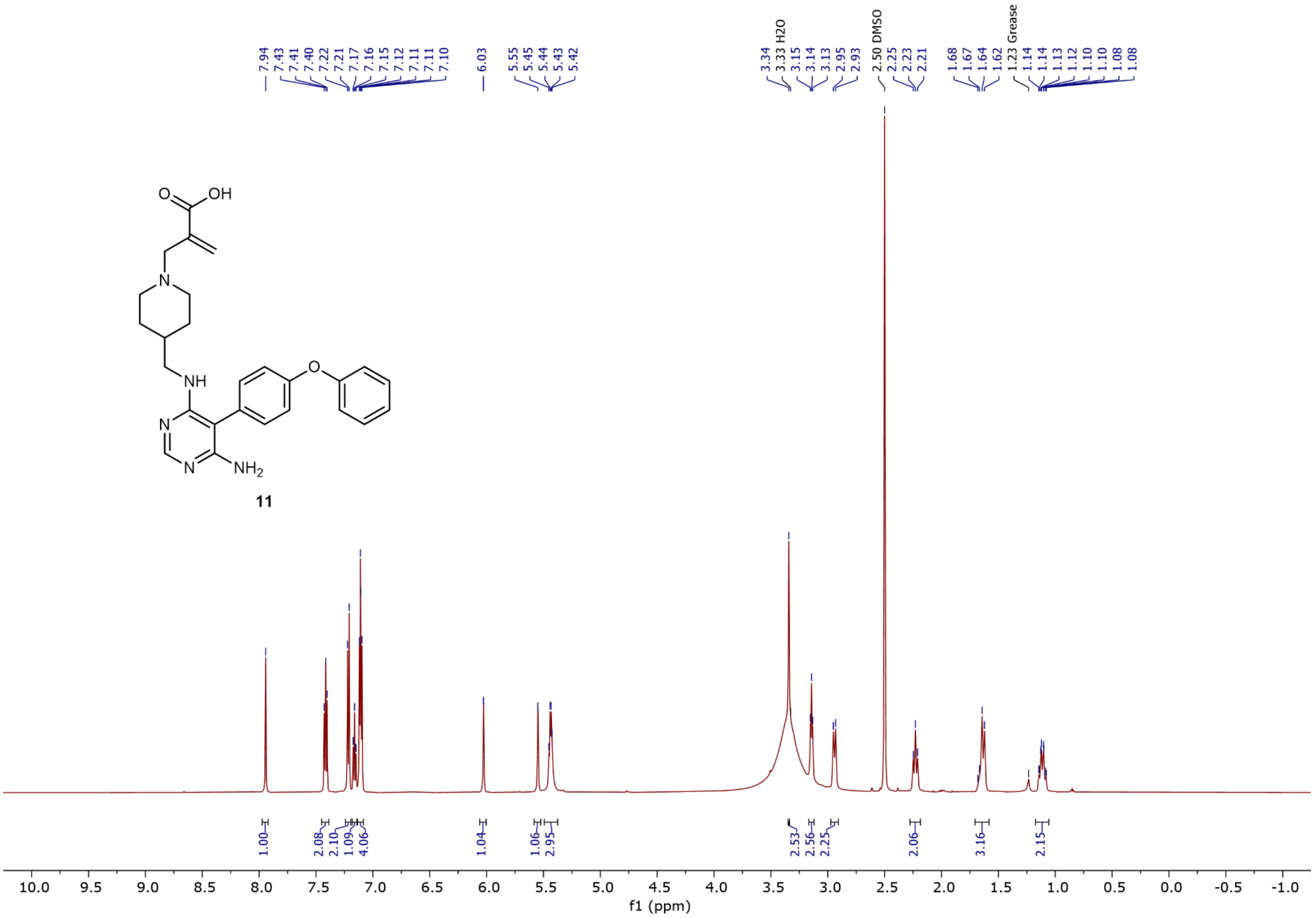

Figure S45. ^{1}H NMR spectrum of compound **11** (600MHz, $(CD_3)_2SO$).

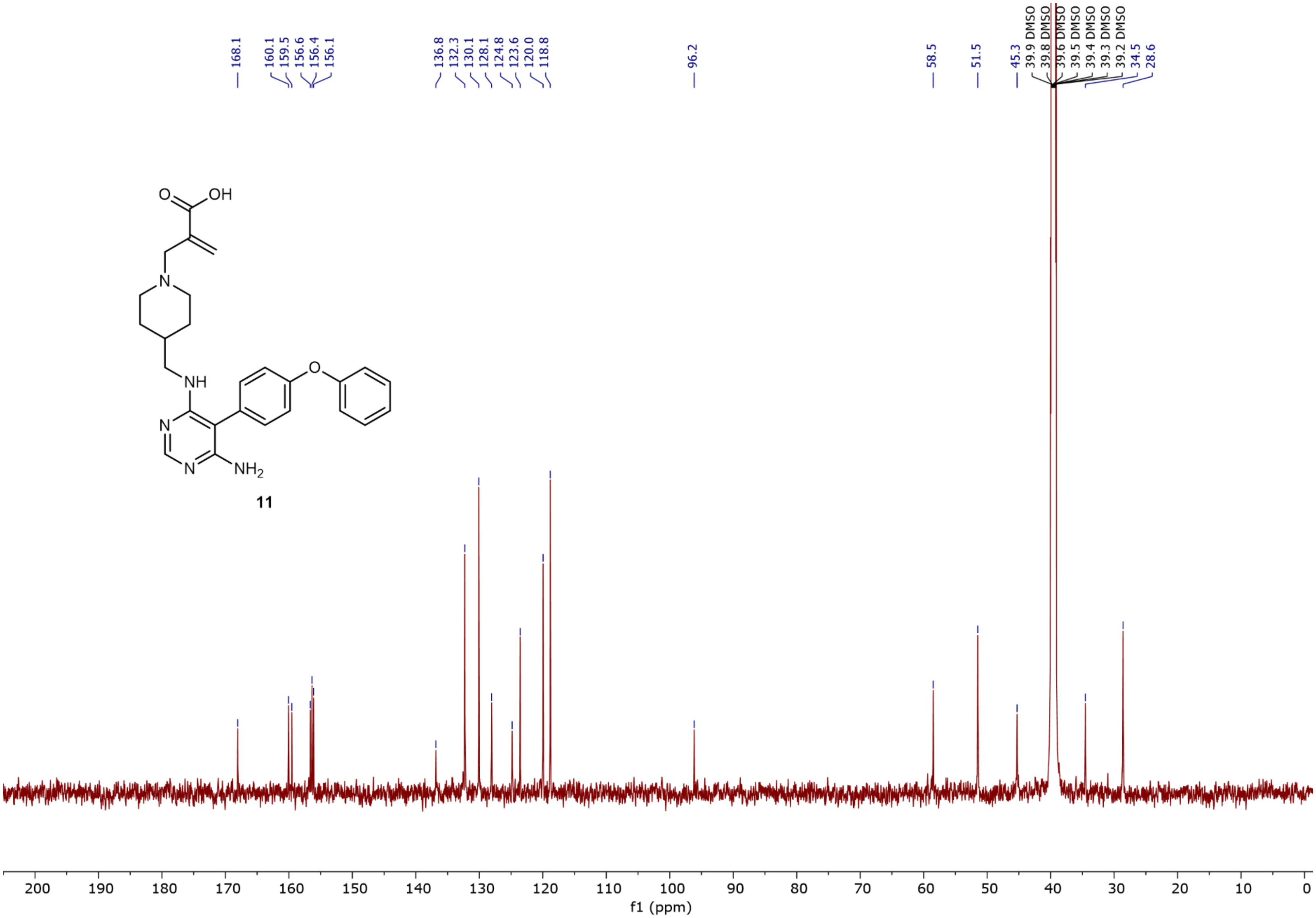

Figure S46. $^{13}C\{^1H\}$ NMR spectrum of compound **11** (176 MHz, $(CD_3)_2SO$).

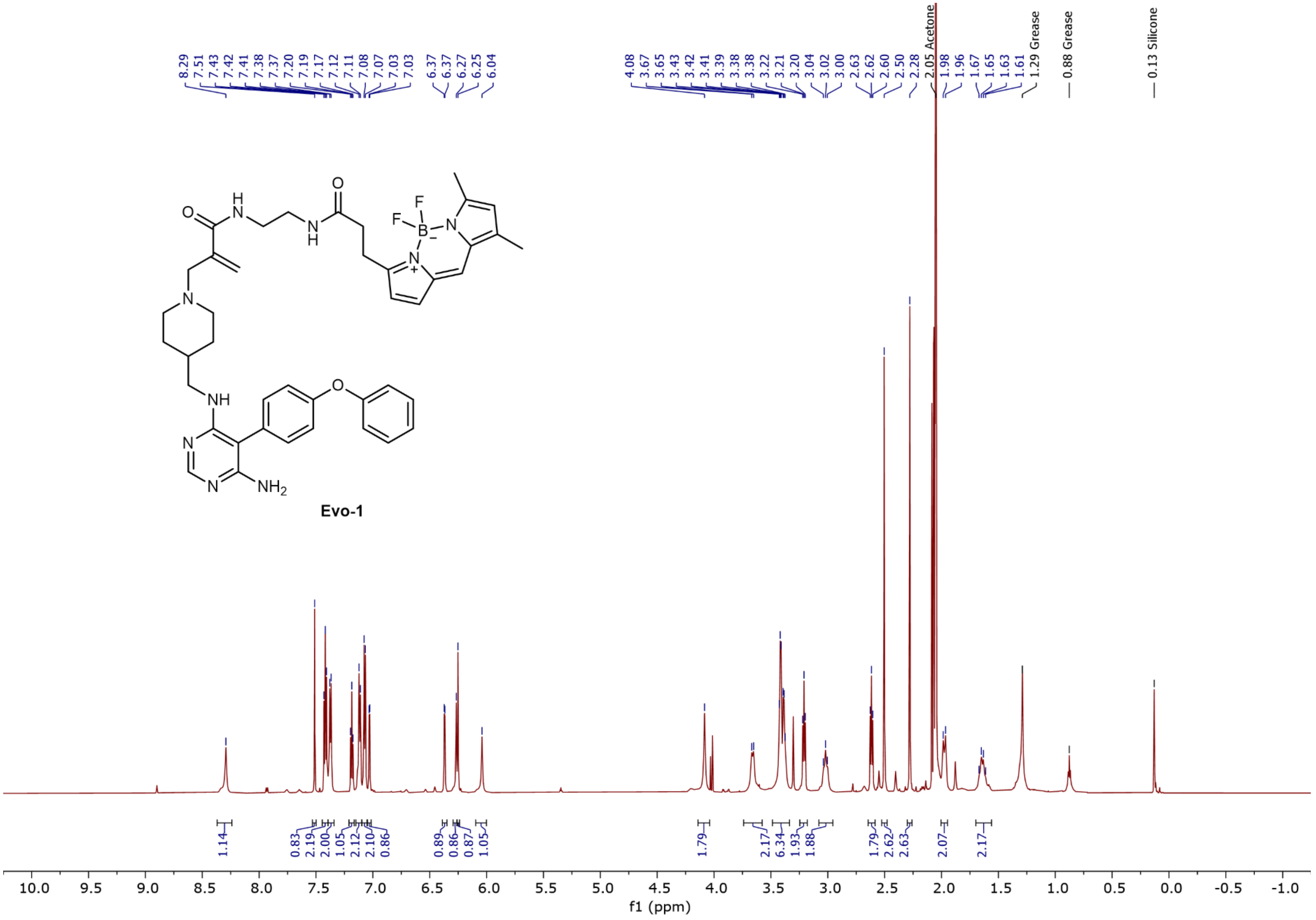

Figure S47. ^{1}H NMR spectrum of **Evo-1** (700MHz, $(CD_3)_2CO$).

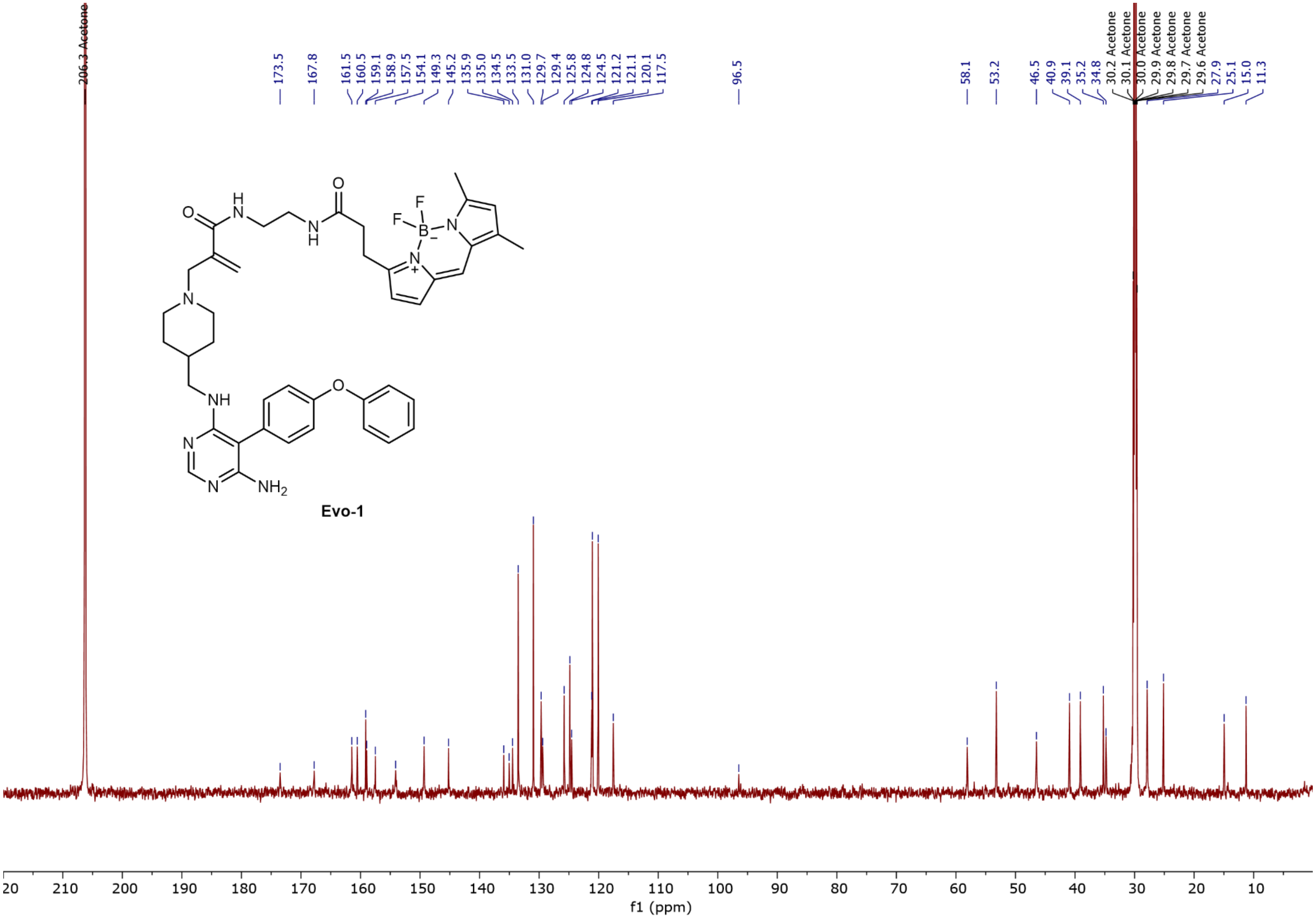

Figure S48. $^{13}C\{^{1}H\}$ NMR spectrum of **Evo-1** (176 MHz, $(CD_3)_2CO$).

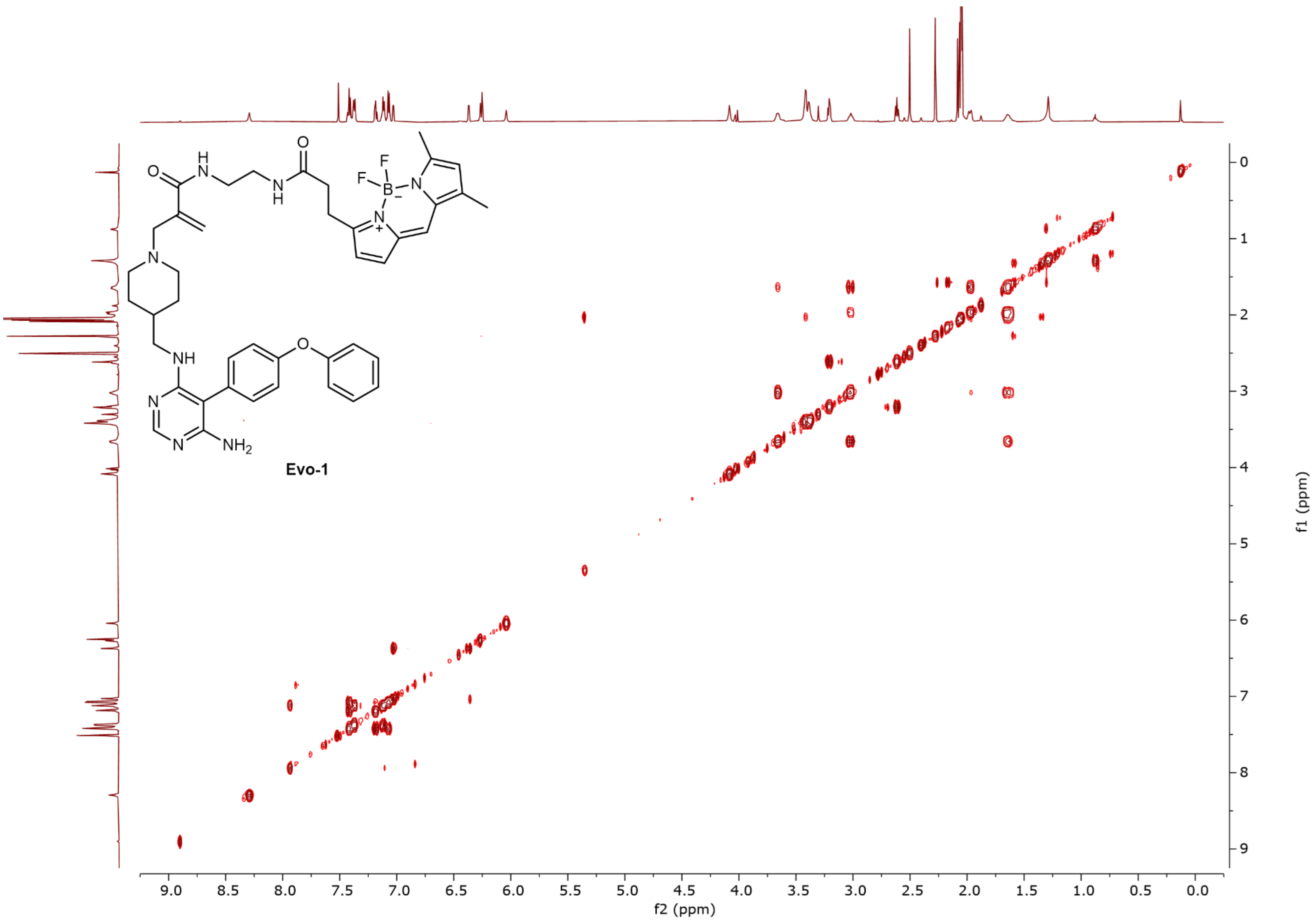

Figure S49. ^{1}H-^{1}H COSY spectrum of **Evo-1** [$(CD_3)_2CO$].

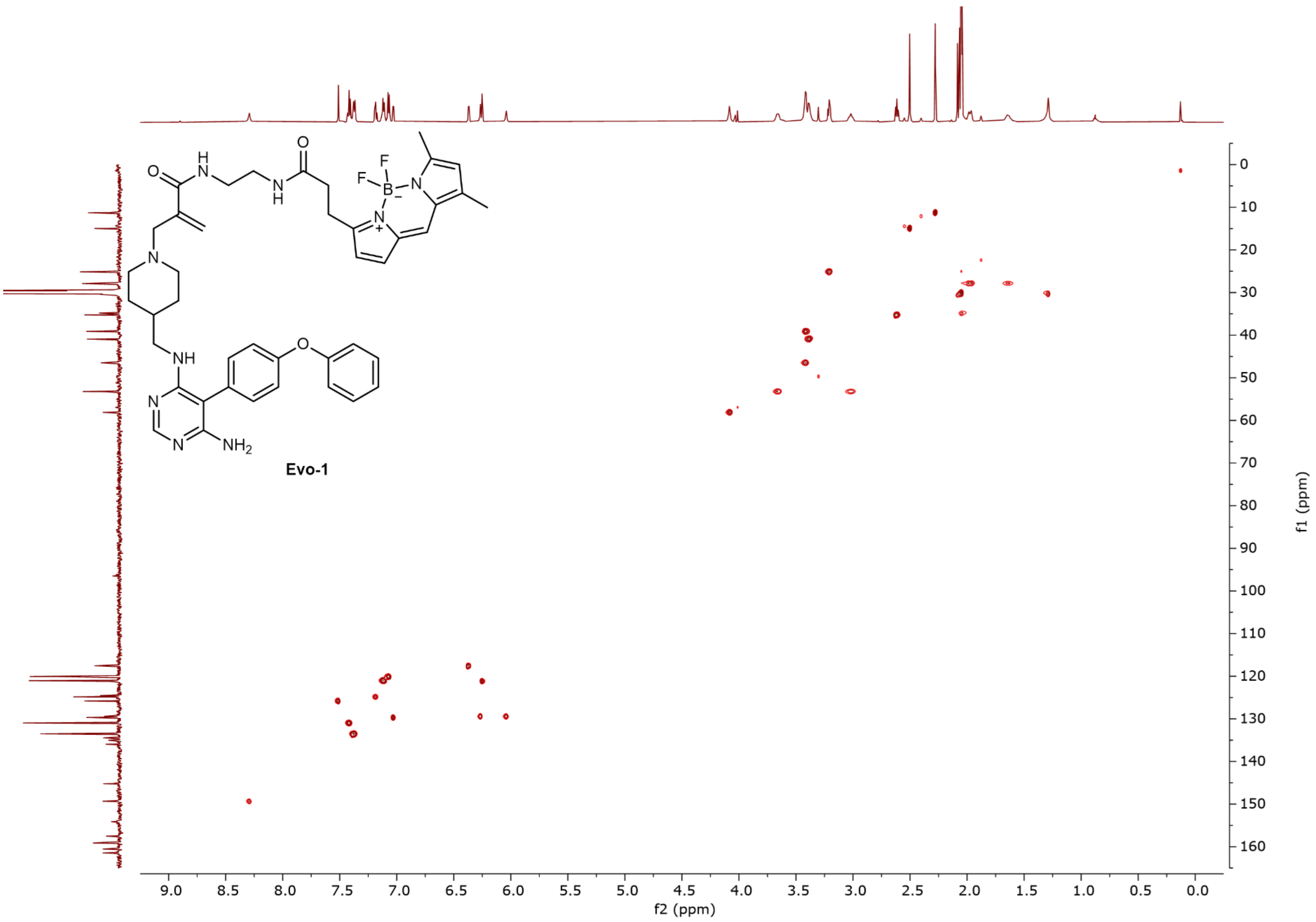

Figure S50. ^{1}H-^{13}C HSQC spectrum of **Evo-1** [$(CD_3)_2CO$].

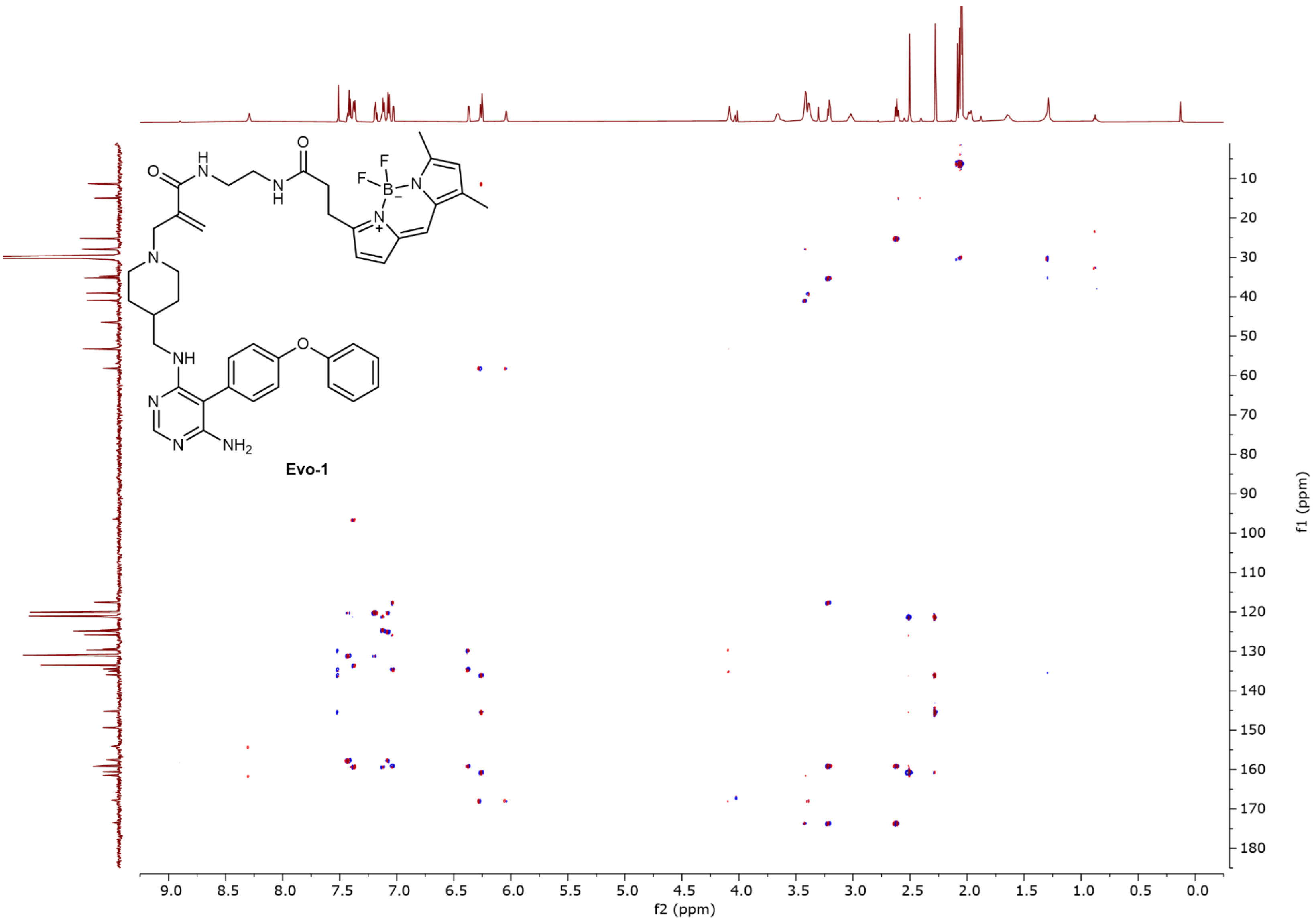

Figure S51. ^{1}H-^{13}C HMBC spectrum of **Evo-1** [$(CD_3)_2CO$].

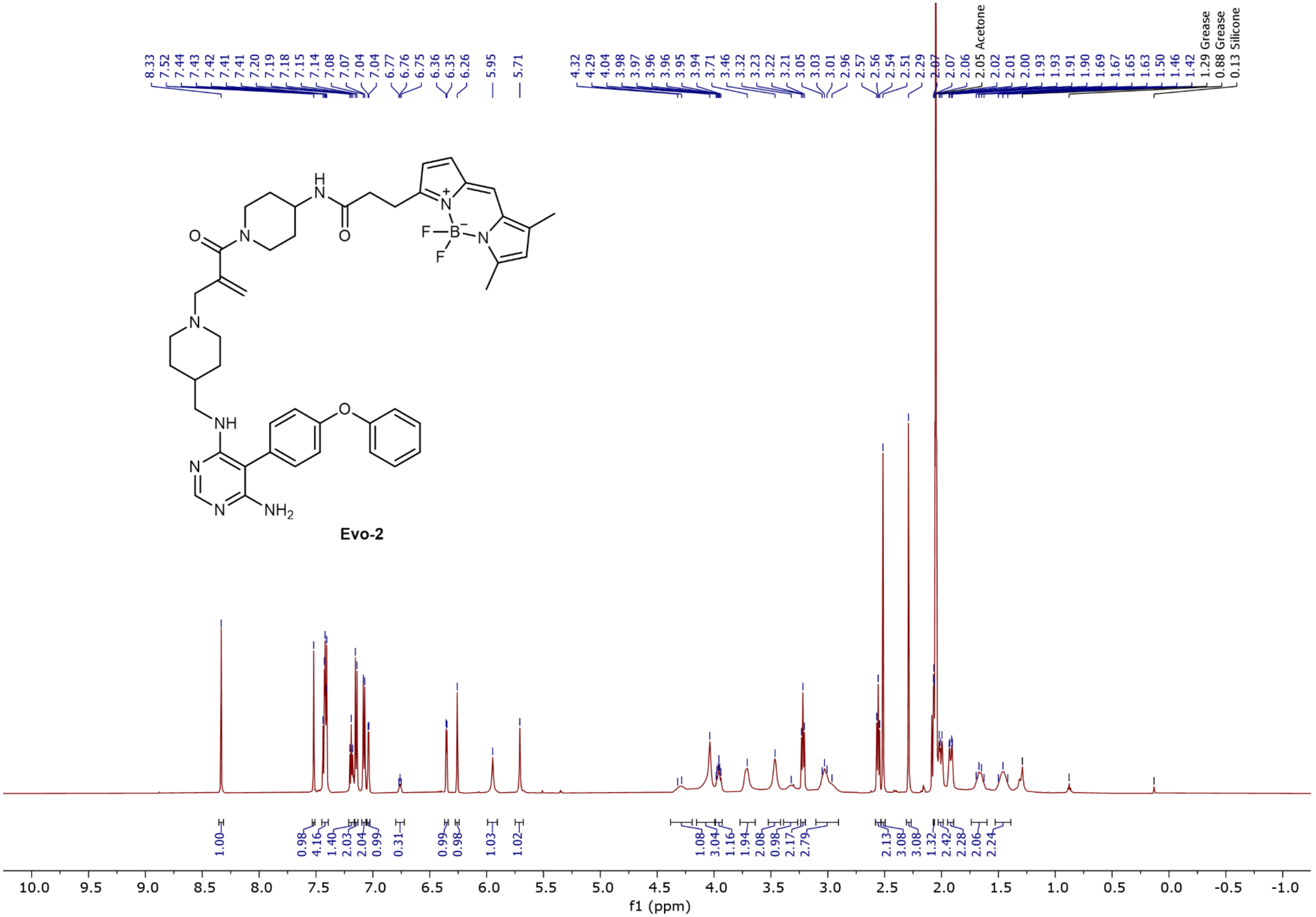

Figure S52. ¹H NMR spectrum of **Evo-2** (600MHz, $(CD_3)_2CO$).

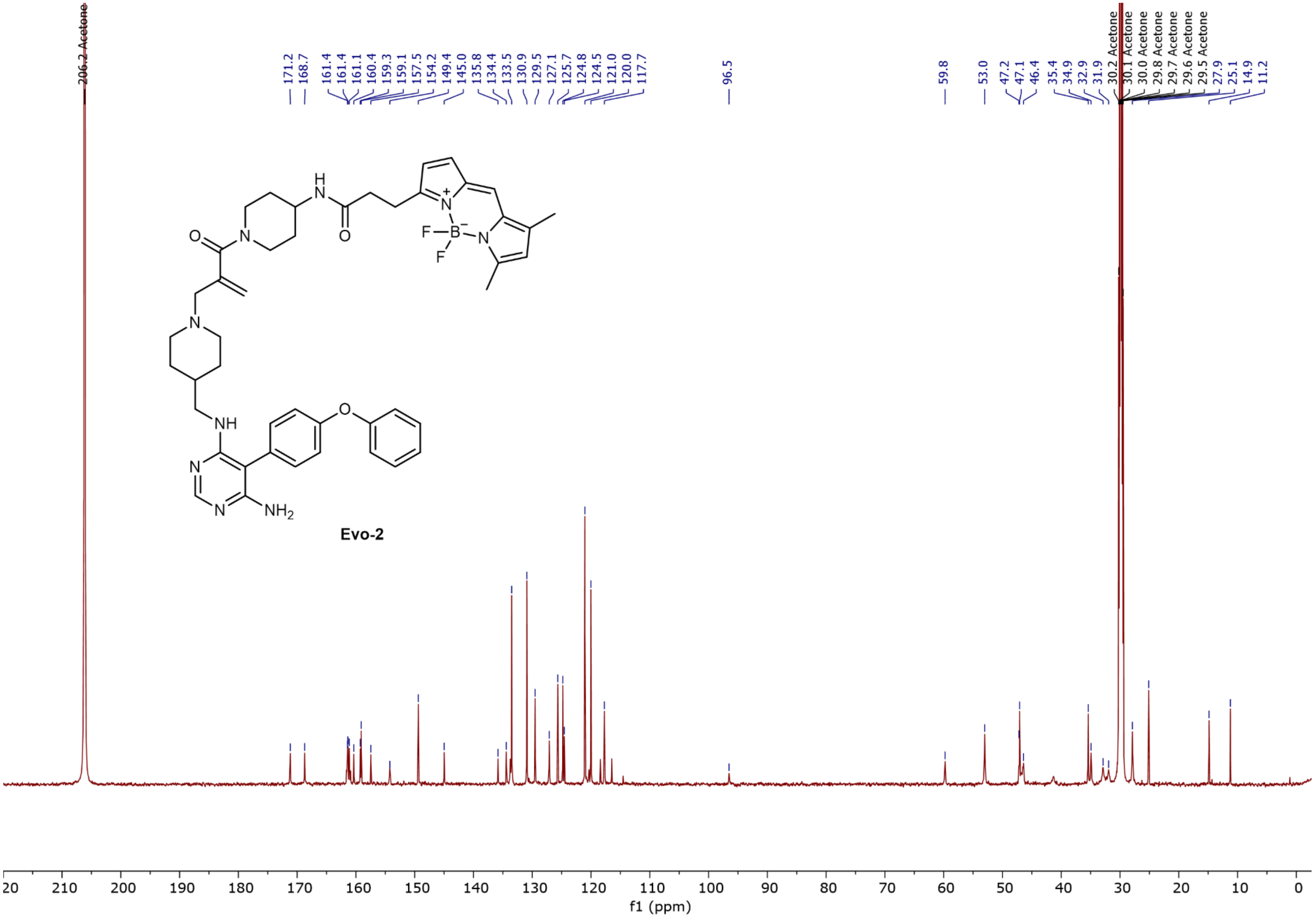

Figure S53. $^{13}C\{^{1}H\}$ NMR spectrum of **Evo-2** (151 MHz, $(CD_3)_2CO$).

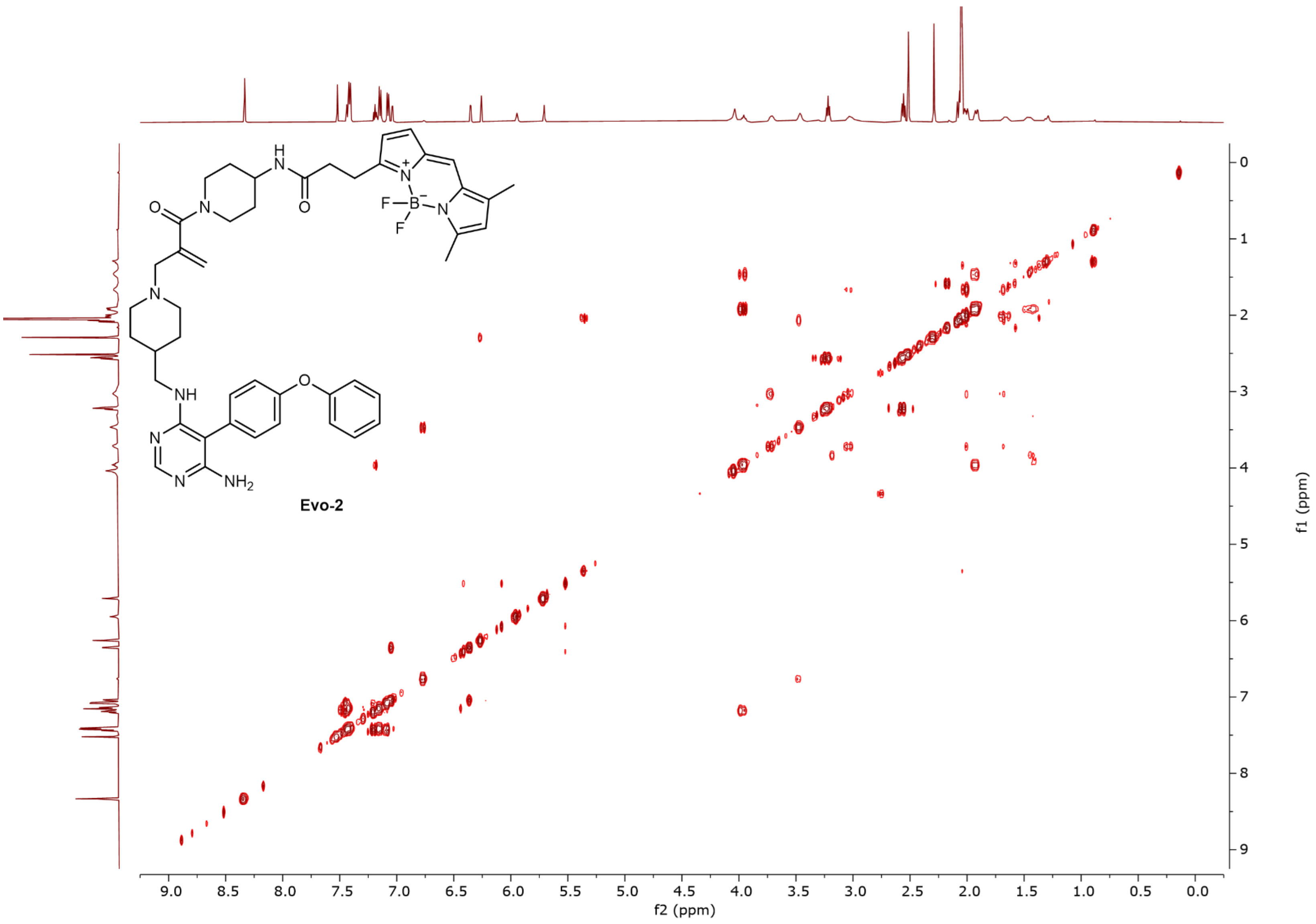

Figure S54. ^{1}H-^{1}H COSY spectrum of **Evo-2** [$(CD_3)_2CO$].

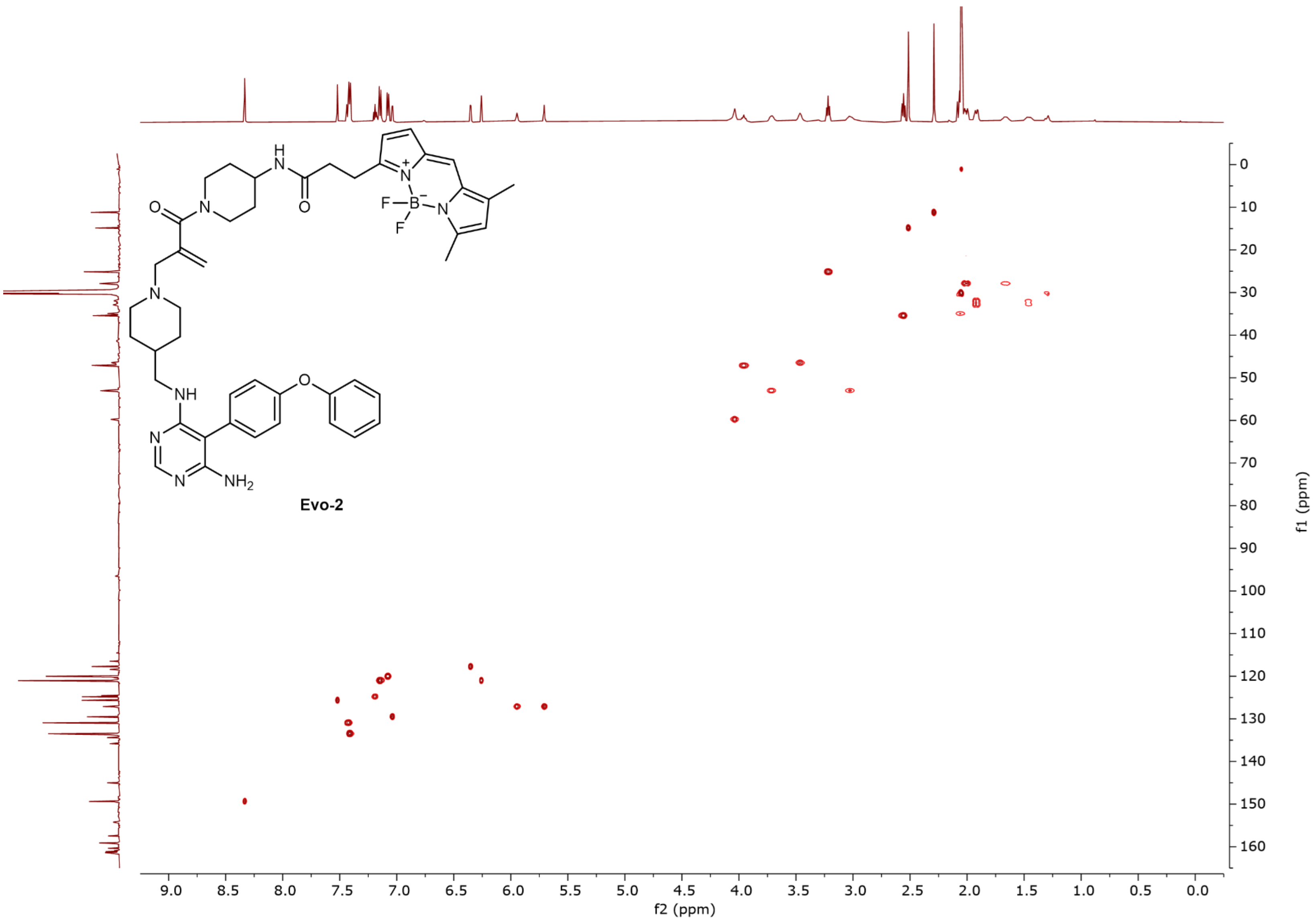

Figure S55. ^{1}H-^{13}C HSQC spectrum of **Evo-2** [$(CD_3)_2CO$].

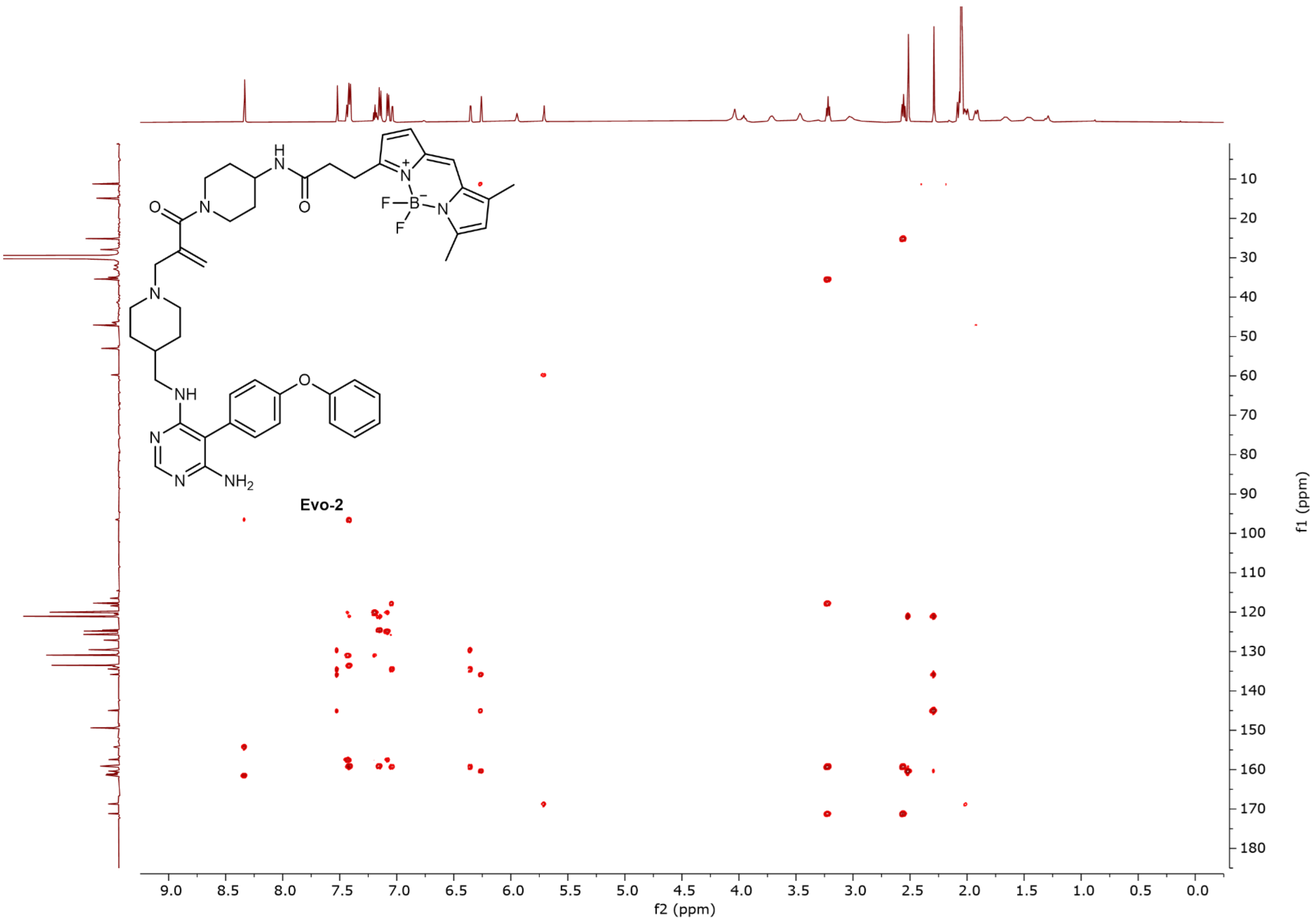

Figure S56. ^{1}H-^{13}C HMBC spectrum of **Evo-2** [$(CD_3)_2CO$].

HRMS Spectra

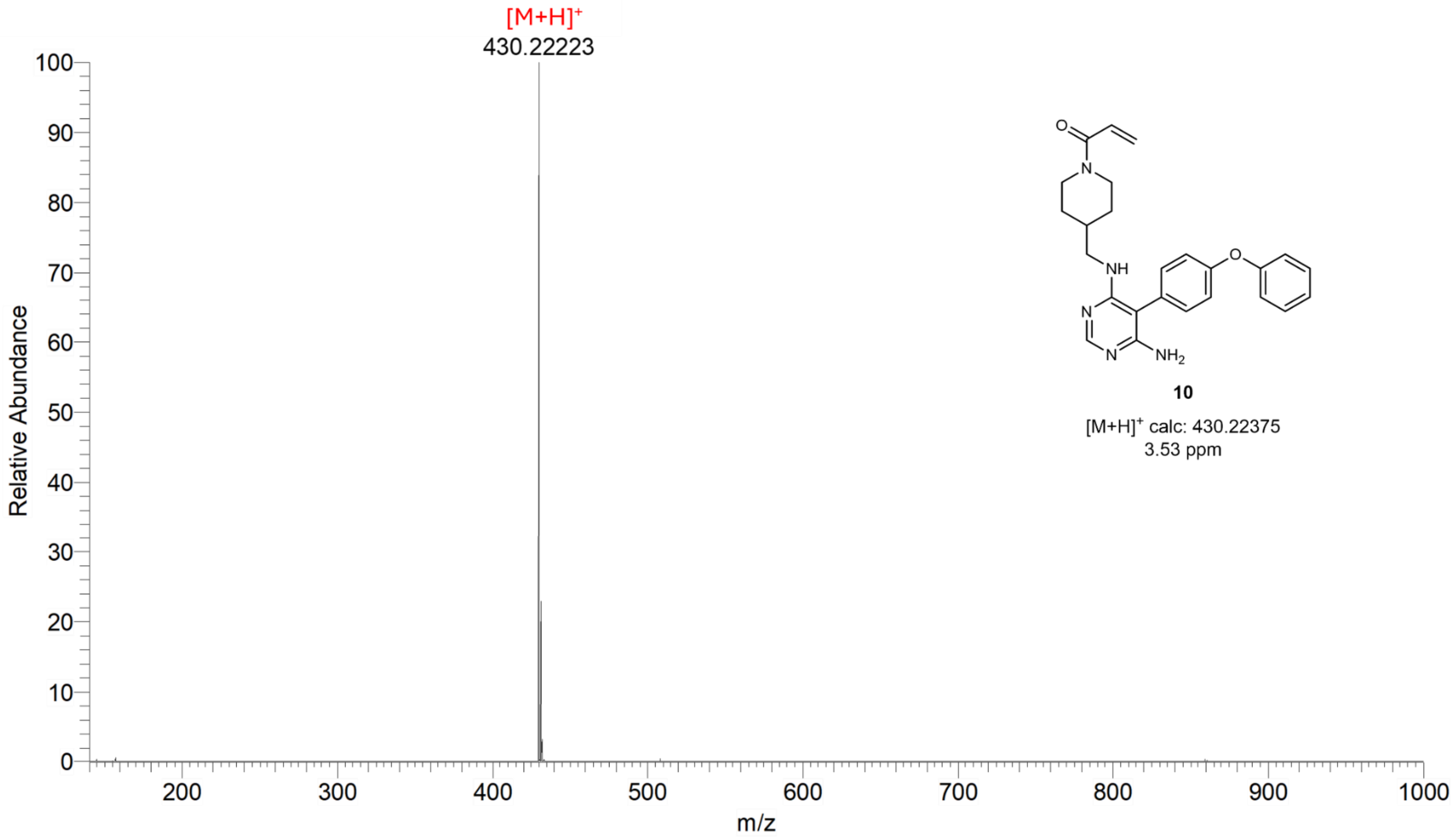

Figure

Figure S57. HRMS spectrum of compound **10**.

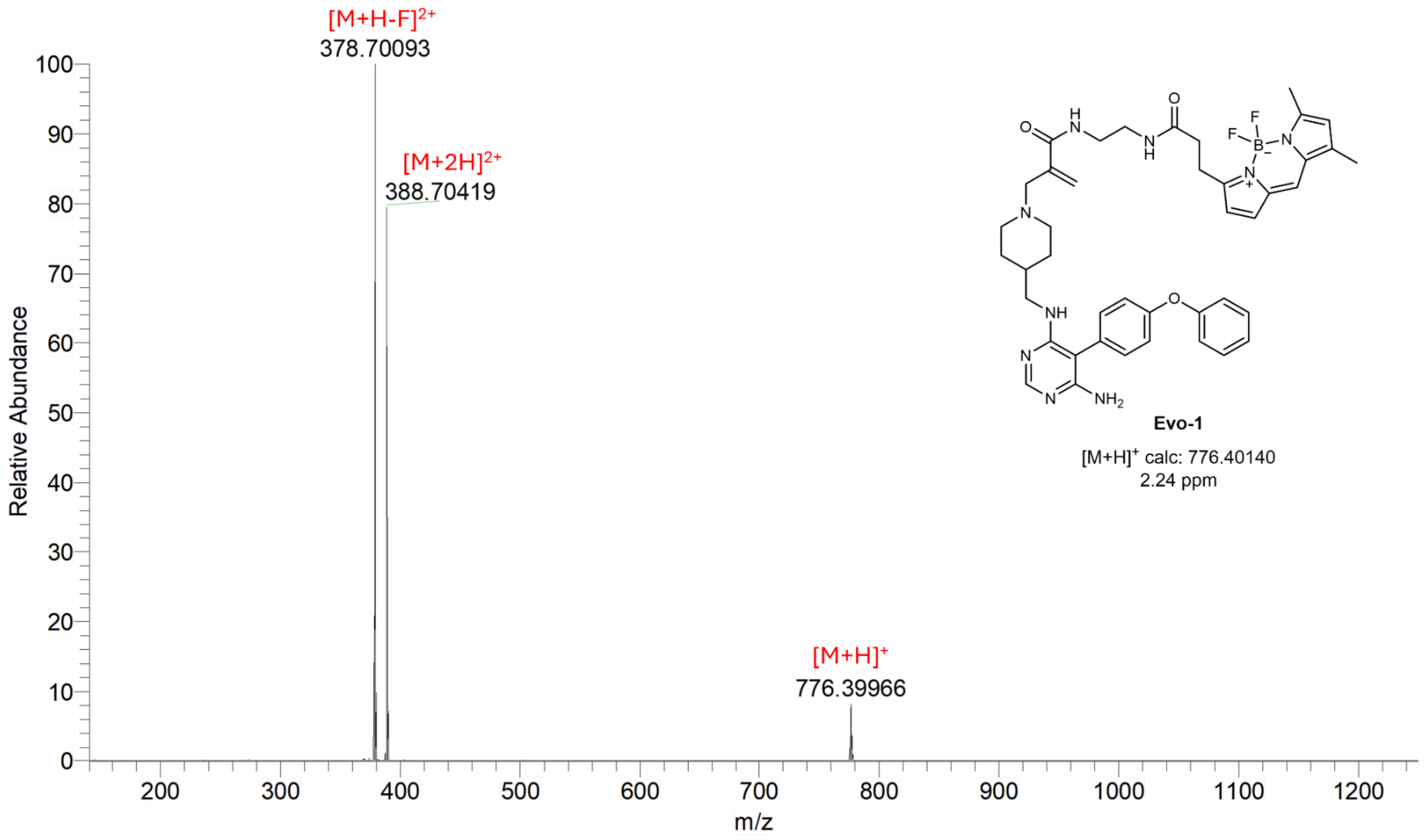

Figure S58. HRMS spectrum of **Evo-1**.

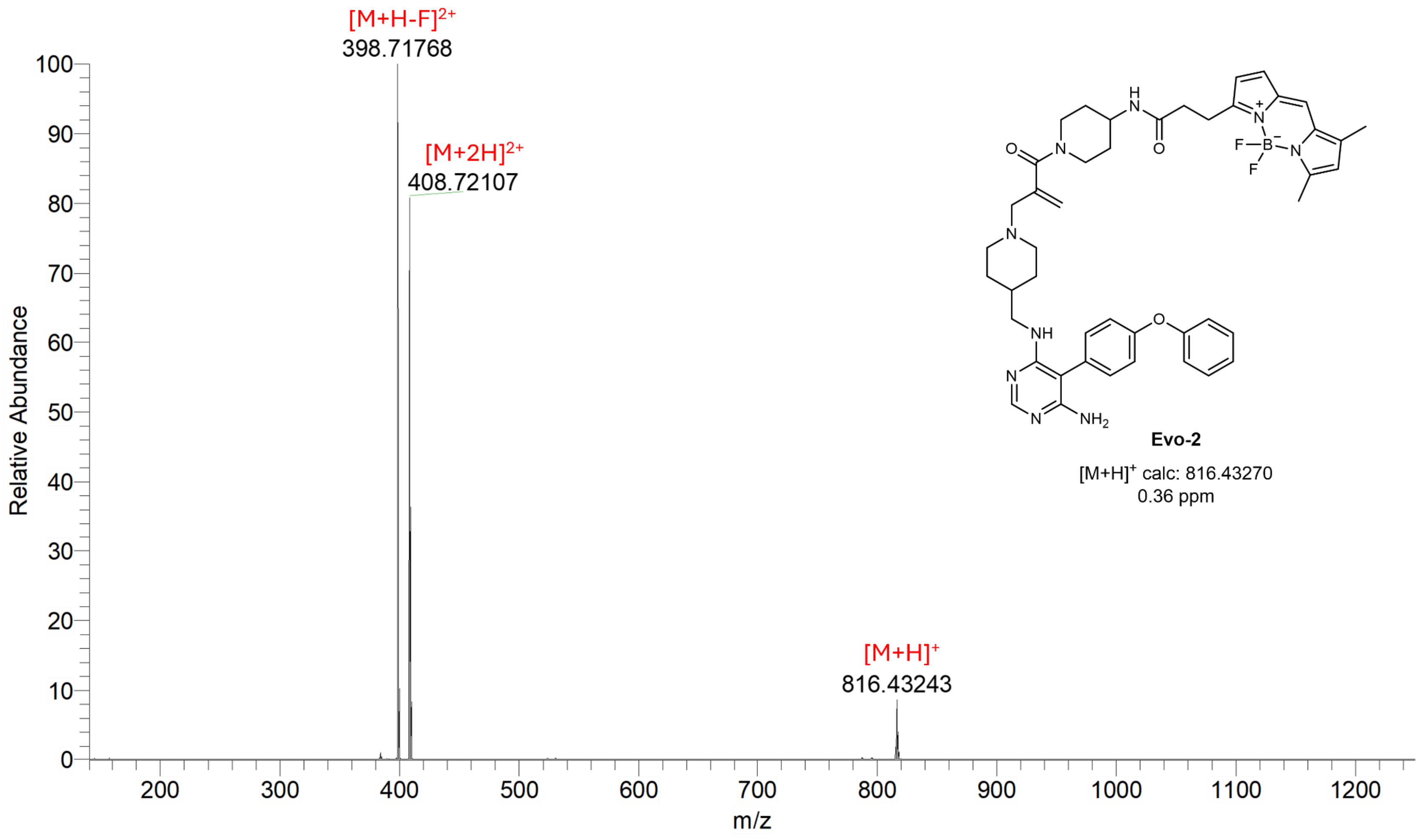

Figure S59. HRMS spectrum of **Evo-2**.

HPLC Chromatograms

2

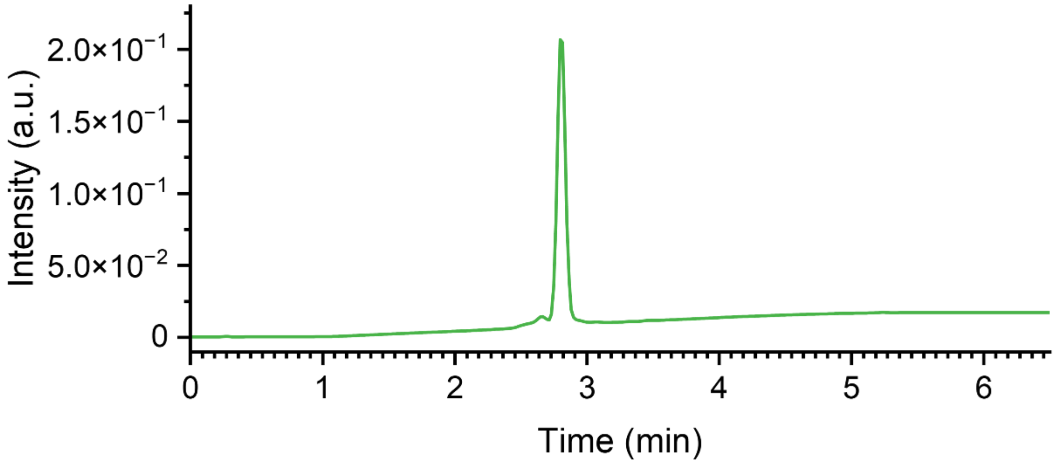

Figure S60. UV spectrum (sum of absorption at 254 nm and 360 nm) of the LC-MS analysis of compound **2**. a.u. = absorbance units.

9

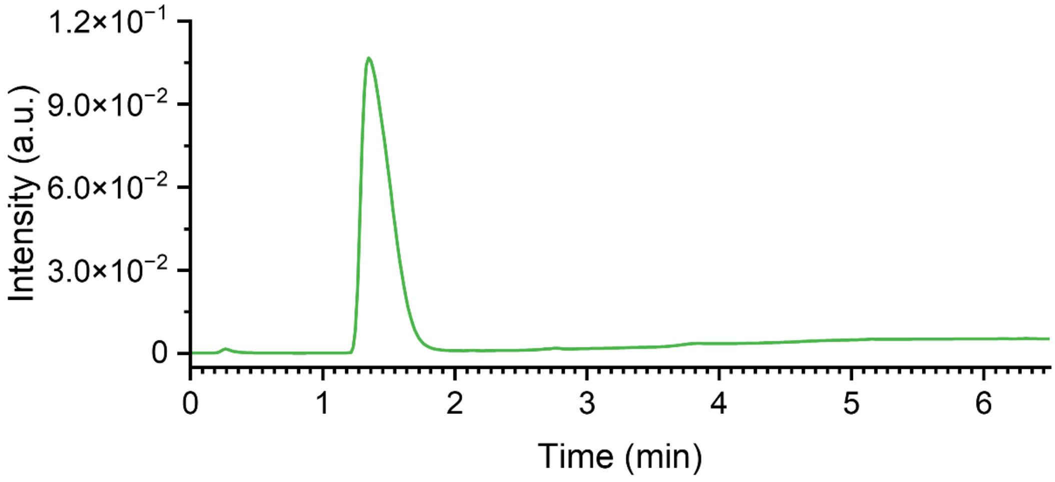

Figure S61. UV spectrum (sum of absorption at 230 nm and 254 nm) of the LC-MS analysis of compound **9**. a.u. = absorbance units.

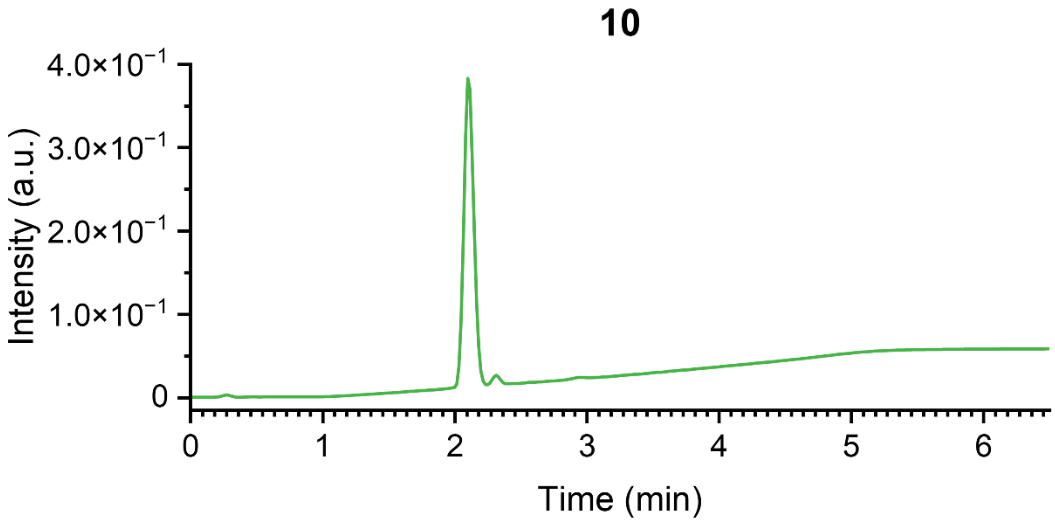

Figure S62. UV spectrum (sum of absorption at 230 nm and 254 nm) of the LC-MS analysis of compound **10**. a.u. = absorbance units.

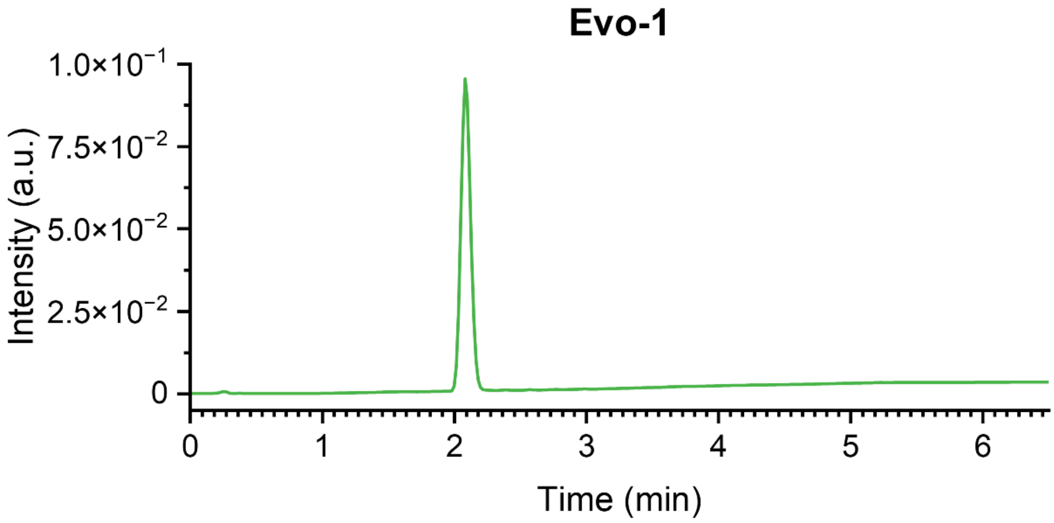

Figure S63. UV spectrum (sum of absorption at 254 nm and 360 nm) of the LC-MS analysis of **Evo-1**. a.u. = absorbance units.

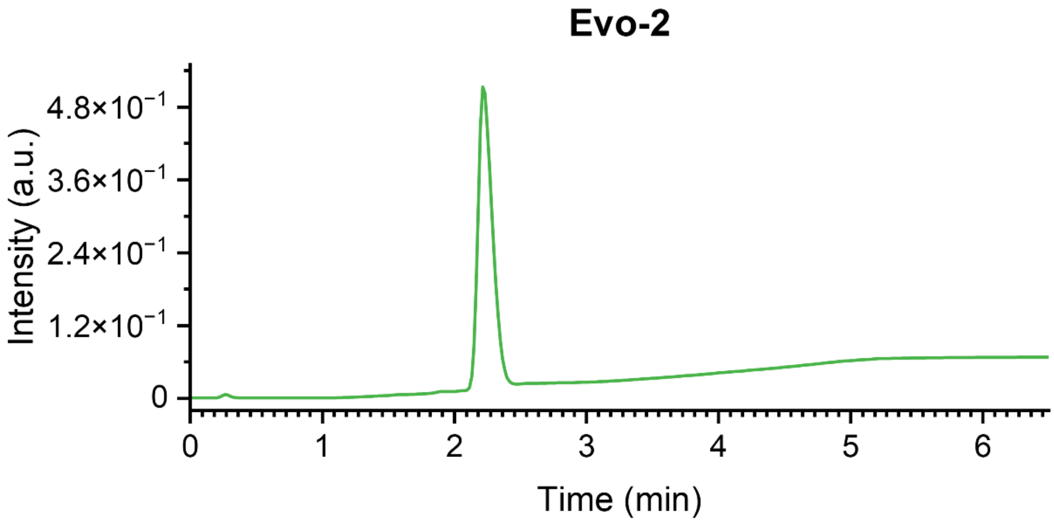

Figure S64. UV spectrum (sum of absorption at 230 nm and 254 nm) of the LC-MS analysis of **Evo-2**. a.u. = absorbance units.

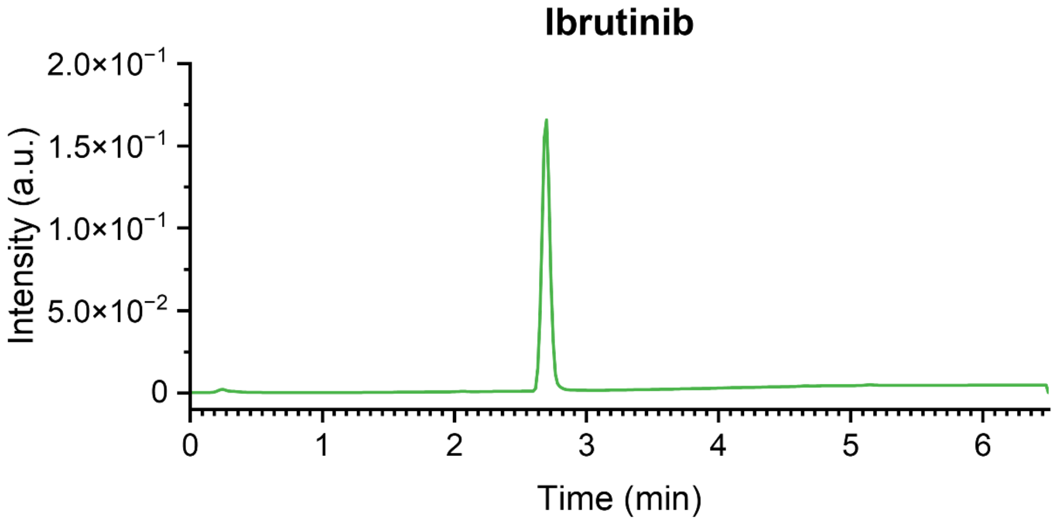

Figure S65. UV spectrum (sum of absorption at 230 nm and 254 nm) of the LC-MS analysis of the purchased ibrutinib. a.u. = absorbance units.

References

1 S. Krajcovicova, J. Stankova, P. Dzubak, M. Hajduch, M. Soural and M. Urban, *Chemistry – A European Journal*, 2018, **24**, 4957–4966.

2 R. D. Caldwell, H. Qiu, B. C. Askew, A. T. Bender, N. Brugger, M. Camps, M. Dhanabal, V. Dutt, T. Eichhorn, A. S. Gardberg, A. Goutopoulos, R. Grenningloh, J. Head, B. Healey, B. L. Hodous, B. R. Huck, T. L. Johnson, C. Jones, R. C. Jones, I. Mochalkin, F. Morandi, N. Nguyen, M. Meyring, J. R. Potnick, D. C. Santos, R. Schmidt, B. Sherer, A. Shutes, K. Urbahns, A. V. Follis, A. A. Wegener, S. C. Zimmerli and L. Liu-Bujalski, *J. Med. Chem.*, 2019, **62**, 7643–7655.

3 R. N. Reddi, A. Rogel, E. Resnick, R. Gabizon, P. K. Prasad, N. Gurwicz, H. Barr, Z. Shulman and N. London, *J. Am. Chem. Soc.*, 2021, **143**, 20095–20108.
4 C. S. Hughes, S. Foehr, D. A. Garfield, E. E. Furlong, L. M. Steinmetz and J. Krijgsveld, *Molecular Systems Biology*, 2014, **10**, 757.
5 C. A. Schneider, W. S. Rasband and K. W. Eliceiri, *Nat Methods*, 2012, **9**, 671–675.
6 S. Bolte and F. P. Cordelières, *Journal of Microscopy*, 2006, **224**, 213–232.
